# Thermally activated delayed fluorescent phenothiazine–dibenzo[*a*,*j*]phenazine–phenothiazine triads exhibiting tricolor-changing mechanochromic luminescence†
†Electronic supplementary information (ESI) available: Synthetic procedures, spectroscopic data, copies of NMR charts, physicochemical properties, and device fabrication and performances. CCDC 1452024. For ESI and crystallographic data in CIF or other electronic format see DOI: 10.1039/c6sc04863c



**DOI:** 10.1039/c6sc04863c

**Published:** 2017-01-11

**Authors:** Masato Okazaki, Youhei Takeda, Przemyslaw Data, Piotr Pander, Heather Higginbotham, Andrew P. Monkman, Satoshi Minakata

**Affiliations:** a Department of Applied Chemistry , Graduate School of Engineering , Osaka University , Yamadaoka 2-1, Suita , Osaka 565-0871 , Japan . Email: takeda@chem.eng.osaka-u.ac.jp ; Email: minakata@chem.eng.osaka-u.ac.jp; b Physics Department , Durham University , South Road , Durham DH1 3LE , UK . Email: przemyslaw.data@durham.ac.uk; c Faculty of Chemistry , Silesian University of Technology , M. Stzody 9 , 44-100 Gliwice , Poland

## Abstract

Novel U-shaped donor–acceptor–donor (D–A–D) π-conjugated multi-functional molecules comprising dibenzo[*a*,*j*]phenazine (DBPHZ) as an acceptor and phenothiazines (PTZ) as donors have been developed. Most importantly, the D–A–D compounds exhibit not only distinct tricolor-changeable mechanochromic luminescence (MCL) properties but also efficient thermally activated delayed fluorescence (TADF). Quantum chemical calculations, X-ray diffraction analysis, and systematic studies on the photophysical properties indicated that the “two-conformation-switchable” PTZ units play a highly important role in achieving multi-color-changing MCL. Time-resolved photophysical measurements revealed that the developed D–A–D compounds also exhibit efficient orange-TADF. Furthermore, organic light-emitting diode (OLED) devices fabricated with the new TADF emitters have achieved high external quantum efficiencies (EQEs) up to 16.8%, which significantly exceeds the theoretical maximum (∼5%) of conventional fluorescent emitters.

## Abstract 2

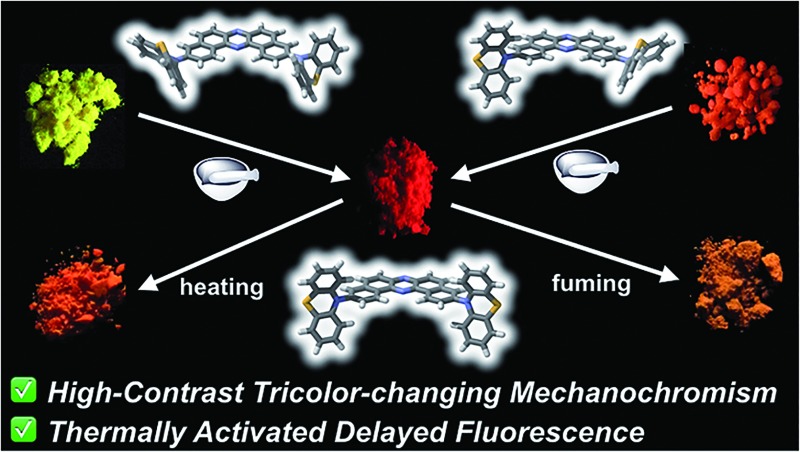
Incorporation of switchable donors into an acceptor core led to tricolor luminescence and thermally activated delayed fluorescence.

## Introduction

Mechanochromic luminescent (MCL) materials,^1^ which exhibit reversible distinct luminescence color changes in response to external stimuli such as mechanical forces (*e.g.*, grinding, pressing, rubbing, and shearing), temperature, vapor, and electric field, have found many applications in optoelectronic devices, sensors, probes, optical data storage devices, and security inks.^2^ Most of the MCL materials developed so far involve organic crystalline compounds,^3^ organometallic crystalline compounds,^4^ and liquid-crystalline compounds,^5^ and show reversible two-color switching MCL properties: an emission color (denoted “state A”) is typically changed by mechanical forces to a different color (denoted “state B”), which is again reverted back to the original color (“state A”) by thermal treatment and/or solvent exposure. From a mechanistic point of view, such two-color-changing MCL behavior is caused by reversible changes in chemical structures (*i.e.* the breaking and reforming of chemical bonds) or in physical structures (*e.g.* polymorphs and packing structures) between thermodynamically metastable and stable states.

In this context, distinct multi-color-changing (more than 3 colors) MCL compounds would be more promising materials for sensitive sensing of their environments (*e.g.* pressure, temperature, and pH). Therefore, the last 5 years have witnessed the emergence of multi-color-changing MCL materials.^6–8^ In 2011, Kato pioneered distinct tricolor-switching MCL systems (green-yellow-red) comprising a single luminophore liquid-crystal by controlling complex self-assembled structures.^7*a*^ Zou and Tian found that bis(pyridylvinyl)anthracene (BP2VA) significantly exhibits pressure-dependent luminochromism ranging from green to red.^7*c*^ Saito and Yamaguchi nicely demonstrated the distinct difference in the luminochromic behavior of tetrathiazolylthiophene in response to anisotropic grinding and isotropic compression.^7*e*^ The integration of two different fluorophores into one molecule has also been proven to be an effective strategy for tricolored MCL materials by Jian and co-workers.^7*f*^ Yagai and Ito devised an amphiphilic dipolar π-conjugated molecule exhibiting variable emission colors depending on changes in the (liquid-)crystalline phases.^7*g*^ Uekusa and Ito developed a tetracolored fluorochromic system based on crystal-to-crystal-to-amorphous phase transitions.^7*h*^ More recently, Zhang, Zou and Ma reported that a donor–acceptor (D–A) type fluorescent molecule with intramolecular charge transfer (ICT) character shows multi-color MCL behavior caused by the change in twisting angles between the D and A units.^7*i*^ In addition to these single molecule systems, the utilization of exciplex formation between D and A components has been emerging as another solution in order to create multi-color-switching MCL materials.^8^ Despite these excellent studies, the development of such distinct multi-color-changing and value-added MCL materials is still challenging partly due to the lack of general rational design principles thereof.

**Fig. 1:**
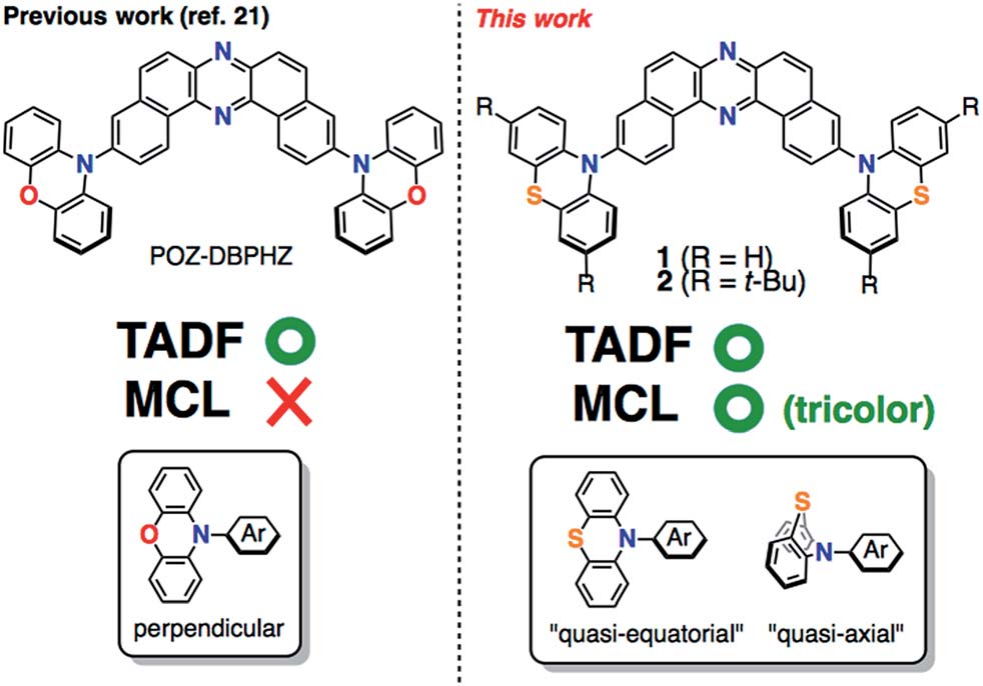
Structures of DBPHZ-cored D–A–D molecules.

A new subset of optically functional materials is thermally activated (or “E-type”)^9^ delayed fluorescence (TADF) materials,^10^ which have found a wide range of applications such as emitters^11^ and host materials^12^ in organic light emitting diodes (OLEDs),^11^ chemiluminescence emitters,^13^ and bioimaging probes.^14^ Specifically, TADF emitters can achieve an internal quantum efficiency in OLEDs close to 100% by up-conversion of triplet excited states into the singlet state.^15^ Such a phenomenon occurs when the energy difference, Δ*E*_ST_, between the excited singlet states (S_1_) and triplet states (T_1_) of the molecule is small, typically <0.3 eV,^11,15^ and the local D (or A) triplet (^3^LE) couples to the triplet CT excited (^3^CT) state vibronically to mediate second-order spin-orbit coupling.^16^ To reduce Δ*E*_ST_, twisted D–A structures with an effective HOMO/LUMO spatial separation are appropriate. Due to the molecular design, TADF emitters can be composed of non-metal elements contrary to conventional phosphorescent materials including expensive rare metals such as Pt and Ir. Therefore, they have been intensively studied as next-generation OLED emitting materials.^11,15–19^ With this in mind, we have recently developed novel TADF molecules that comprise U-shaped dibenzo[*a*,*j*]phenazine (DBPHZ)^20^ as an acceptor and diarylamines as donors.^21^ Especially, the phenoxazine–DBPHZ–phenoxazine triad POZ–DBPHZ (Fig. 1, previous work) was found to be an excellent orange-TADF emitter for organic light-emitting devices (OLEDs), achieving an external quantum efficiency (EQE) up to 16%. The effective HOMO/LUMO separation in the almost perpendicularly twisted D–A–D triad allowed efficient intramolecular charge-transfer (ICT) and very small singlet-triplet energy splitting (Δ*E*_ST_ ∼ 20 meV), which resulted in efficient reverse intersystem crossing (rISC) to yield efficient TADF emission.

We envisaged that the merging of MCL and TADF functions would be a significantly powerful strategy for creating multi-functional organic materials. By taking advantage of both phenomena, more complex sensing can be feasible with such materials in principle, where emission colors and/or intensities vary with an increase (or decrease) in surrounding pressure and/or temperature. For instance, they could find applications in stress-, pressure-, and thermo-indicators for high-tech industries like submarines and aerospace in the future. Although a few TADF emitting compounds that exhibit two-color-changing MCL properties have been reported,^22^ to the best of our knowledge, TADF-active multi-color-changing (more than 3) MCL molecules have never been reported. Herein, we present novel TADF-active and distinct tricolor-changing MCL molecules **1** and **2** (Fig. 1) comprising DBPHZ as an acceptor and phenothiazines as donors. Importantly, phenothiazine (PTZ) units play a fundamentally important role in generating multiple thermodynamically (meta)stable states through conformational changes. Furthermore, these unprecedented multi-functional emitting materials have been applied to OLEDs to achieve EQEs as high as 16.8%.

## Results and discussion

### Molecular design

To develop TADF-active multi-color-changing materials, we designed a phenothiazine–DBPHZ–phenothiazine triad **1**, because the bowl-shaped structure of the phenothiazine (PTZ) unit would allow PTZ-substituted molecules to exist as two distinct conformers: one is “quasi-equatorial” and the other is “quasi-axial” (Fig. 1, this work).^23^ Recently, Adachi has reported that a D–A molecule containing a PTZ group as a donor and 2,4,6-triphenyl-1,3,5-triazine as an acceptor exhibited dual-emission with TADF characteristics.^24^ They concluded that two emissive ICT excited states are correlated with two different conformers of the PTZ-substituted molecules. Likewise, more recently, Zhang and Chi have developed an asymmetric D–A–D′ molecule (D = carbazolyl-, D′ = PTZ, and A = benzophenone) which shows TADF dual-emission (white light) in the solid state.^22*a*^ We envisaged that the attachment of more than one PTZ unit to our acceptor-core (DBPHZ) would be a promising molecular design strategy in order to achieve both multi-color-changing MCL and TADF properties with a single molecule.

**Fig. 2:**
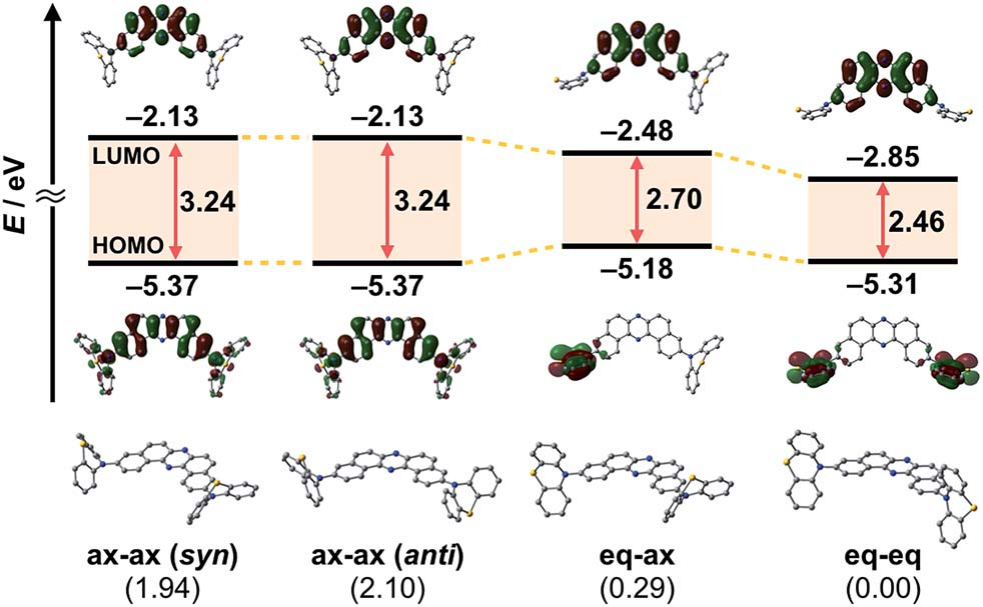
Energy diagram and molecular orbitals of **1** calculated by the DFT method at the B3LYP/6-31+G(d,p) level of theory (values in parentheses shown under the molecules indicate the relative energies of the conformers in kcal mol^–1^).

To evaluate the viability of this molecular design, we initially conducted theoretical calculations using the DFT method at the B3LYP/6-31+G(d,p) level (see the ESI†). An energy diagram and the frontier orbitals of four possible conformers of **1**, which could be generated from the variation of the PTZ conformation, are illustrated in Fig. 2. The HOMO–LUMO band gaps decrease in the order of quasi-axial/quasi-axial (ax–ax), quasi-equatorial/quasi-axial (eq–ax), and quasi-equatorial/quasi-equatorial (eq–eq) conformers (Fig. 2). Focusing on the HOMO and LUMO of ax–ax, the molecular orbitals are delocalized throughout the whole DBPHZ core in both cases, indicating that the π–π* transition would exclusively dominate in these conformers. In sharp contrast, in eq–ax, the HOMO is localized on one of the PTZ units, while the LUMO is delocalized on the acceptor. Likewise, in eq–eq, the HOMO and LUMO are completely separated. These distributions of the frontier orbitals in the eq–ax and eq–eq conformers would result in effective ICT as previously reported with POZ–DBPHZ.^21^ Although ax–ax conformers are less stable (*ca.* 2.0 kcal mol^–1^) than eq–ax and eq–eq conformers, such small energy differences suggest that all four conformers can be thermodynamically interconvertible and thereby multiple emissive excited states correlated with those conformers can exist.

### Synthesis of the materials

**Fig. 3:**
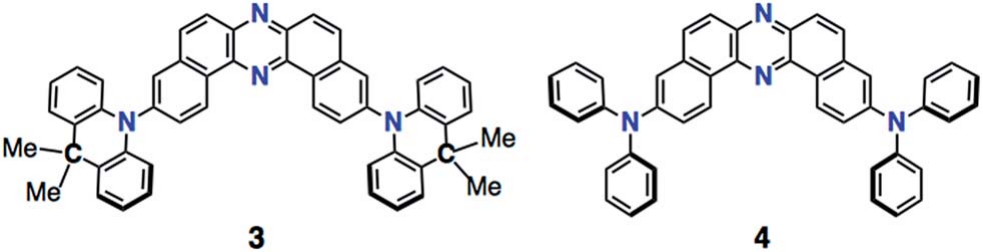
Molecular structures of **3** and **4**.

PTZ-disubstituted DBPHZ **1** and its derivative **2** were successfully synthesized through a Pd-catalyzed Hartwig–Buchwald amination of 3,11-dibromo[*a*,*j*]phenazine^20^ with the corresponding phenothiazines in high yields (see the ESI†). To evaluate the efficacy of the sulfur-bridging in the donor unit, a carbon-bridging analogue **3** (78%) and a non-bridging analogue **4** (67%) were also synthesized in a similar manner (Fig. 3, see the ESI†).

### MCL properties

**Fig. 4:**
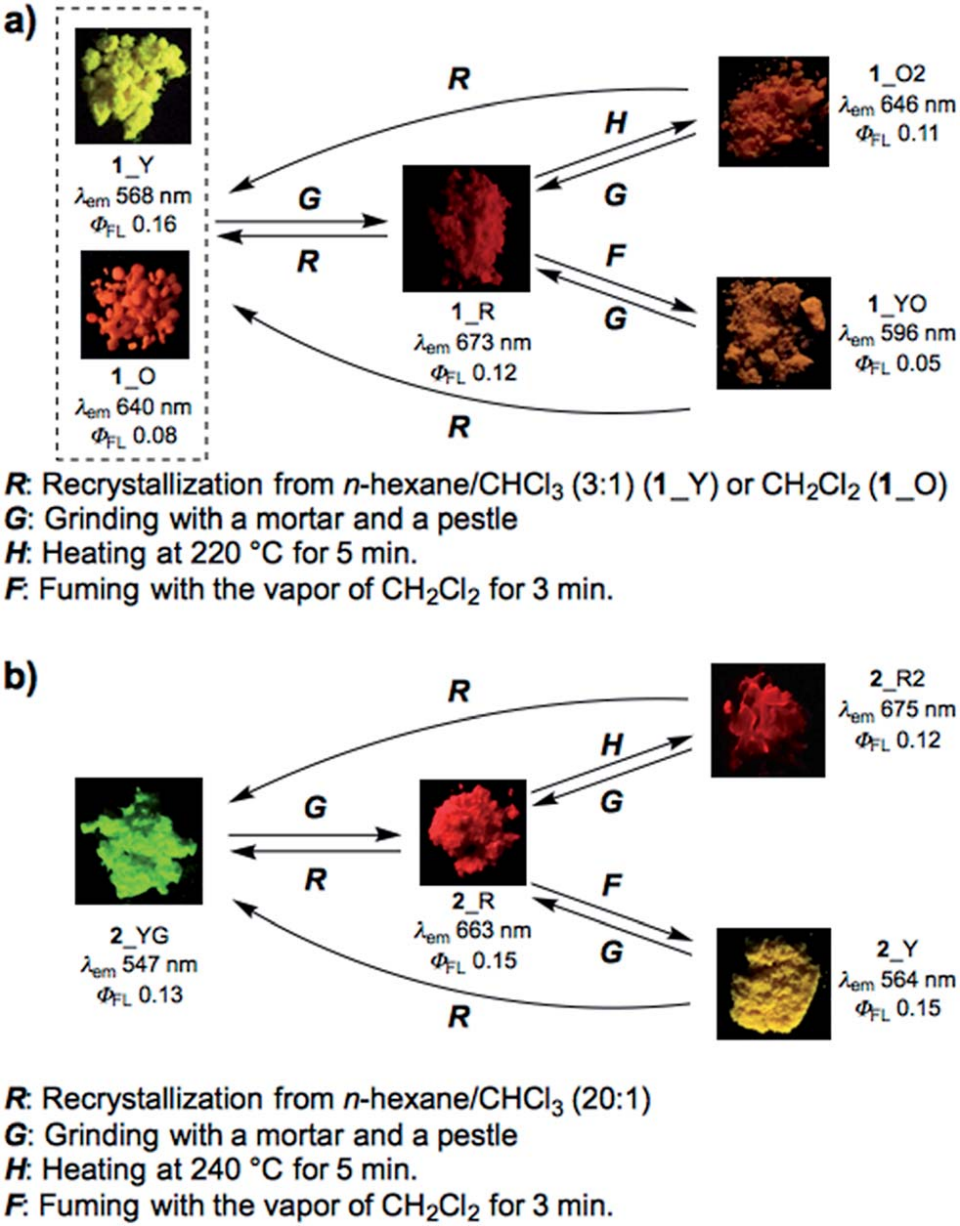
Schematic diagrams of the multi-color-changing MCL behavior of (a) **1** and (b) **2**. Photographs were taken under irradiation with UV light (365 nm).

Most importantly, solid samples of **1** and **2** clearly showed distinct multi-color-changing MCL behavior in response to a variety of external stimuli (*e.g.* recrystallization, grinding, solvent exposure, and heating) (Fig. 4). Two polymorphs of **1** were obtained through recrystallization (**1**_Y and **1**_O, Fig. 4a): needle-like crystals **1**_Y grown from a two-phase solvent of *n*-hexane/CHCl_3_ (3 : 1) exhibited bright yellow emission (*λ*_em_ 568 nm) under UV irradiation, while block-like crystals **1**_O were obtained through slow evaporation of a CH_2_Cl_2_ solution and showed orange emission (*λ*_em_ 640 nm). Notably, upon grinding of **1**_Y and **1**_O with a mortar and pestle, the solid color drastically changed to red in both cases, and the resulting red powder **1**_R emitted deep-red/near IR (NIR) light (*λ*_em_ 673 nm) with a moderate quantum yield (*Φ*_em_ 0.12) for a NIR-emissive organic solid.^25^ Thermal annealing of the red solid **1**_R (220 °C for 5 min) yielded an orange-emissive solid (**1**_O2, *λ*_em_ 646 nm). In contrast, the exposure of **1**_R to CH_2_Cl_2_ vapor caused a greater blue-shift of the emission color (**1**_YO, *λ*_em_ 596 nm). Both of the **1**_O2 and **1**_YO samples reverted back to the ground sample **1**_R by grinding, with high reproducibility. Recrystallization of any of the samples gave **1**_Y or **1**_O, depending on the solvent system used.

Likewise, PTZ-compound **2** also exhibited distinct MCL behavior in response to various external stimuli (Fig. 4b). The needle-like crystals **2**_YG grown from a two-phase solvent of *n*-hexane/CHCl_3_ (20 : 1) showed yellow-green emission (*λ*_em_ 547 nm), and the emission color changed to red through grinding (**2**_R, *λ*_em_ 663 nm) with a large emission energy shift (Δ*ν̃* 3199 cm^–1^). With respect to **2**, other polymorphs were not observed even though various solvent systems were tested in recrystallization. Heating the red solid **2**_R (240 °C for 5 min) caused a slight red-shift of the emission (**2**_R2), which is the opposite behavior when compared to **1**_R. Exposure of **2**_R to CH_2_Cl_2_ vapor clearly changed the emission color to yellow (**2**_Y). Any solid samples reverted back to **2**_YG through the recrystallization process from *n*-hexane/CHCl_3_. As clearly seen from Fig. 4, PTZ-substituted DBPHZs **1** and **2** exhibited distinct tricolor-changing MCL properties.

In sharp contrast, sulfur-free analogues **3** and **4** did not show significant MCL behavior (see Fig. S1c and d in the ESI†). Furthermore, the oxygen-bridged analogue POZ–DBPHZ^21^ (Fig. 1) also did not show significant MCL behavior (Fig. S1e†). These results explicitly indicate that sulfur-bridging of the *N*,*N*-diphenyl unit plays an important role in realizing multi-color-changing MCL characteristics based on DBPHZ-cored D–A–D scaffolds.

In addition to MCL behavior, all the D–A–D molecules **1–4** exhibit acid-induced emission quenching in the solid state (Fig. S3†).^26^ Upon exposure to trifluoroacetic acid (TFA) vapor for 10 s, the ground red samples turned black and luminescence was completely quenched (“turn-off” state), probably due to protonation of the pyrazine unit. In reverse, exposure of these “turn-off state” samples to Et_3_N vapor for 3 h “turned-on” emission in slightly different colors compared to the original emission. These results suggest that the D–A–D molecules are promising for application as acid/base-responsive on–off chemosensors.

### PXRD and DSC measurements

**Fig. 5:**
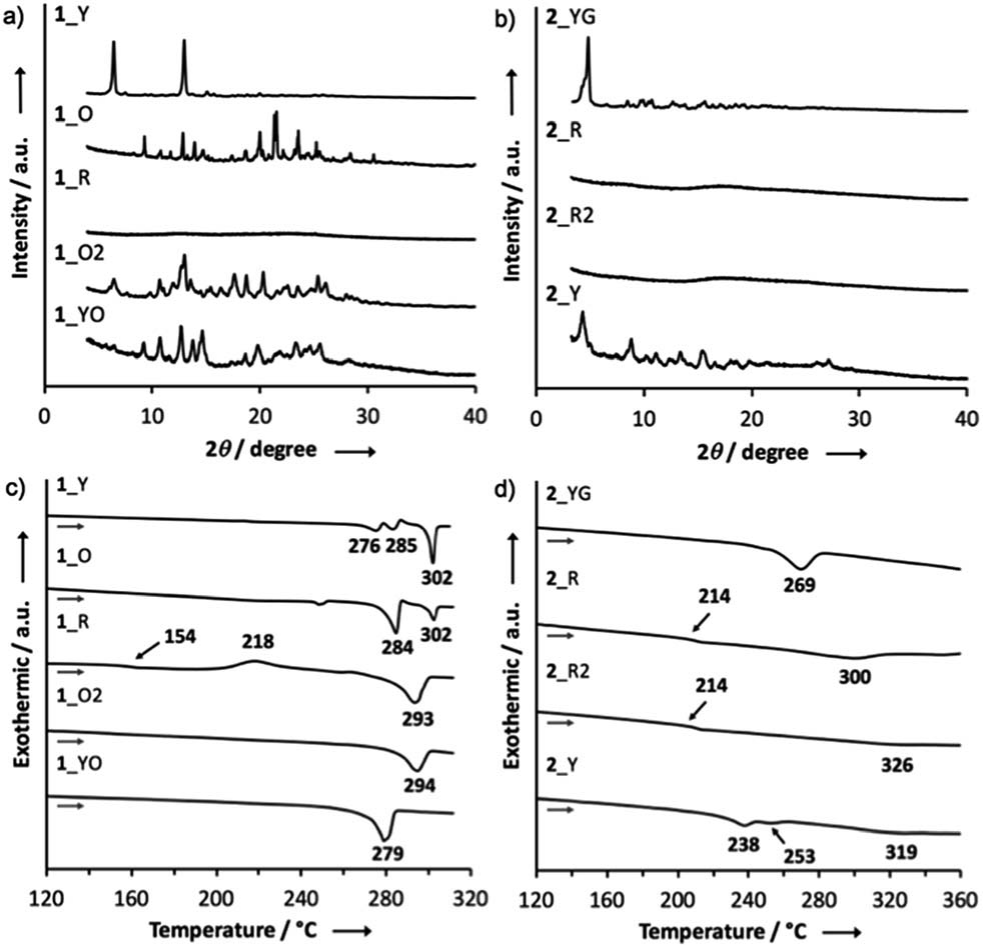
PXRD patterns of (a) **1** and (b) **2**; DSC curves of (c) **1** and (d) **2**.

Powder X-ray diffraction (PXRD) analysis of **1** indicated that the ground solid **1**_R had an amorphous structure, and that the other solids were composed of different crystalline polymorphs (Fig. 5a). With regard to **2**, the PXRD pattern of the crystal **2**_YG was similar to that of **2**_Y, suggesting that solvent vapor can allow the ground solid **2**_R to partly turn into the crystalline phase **2**_YG (Fig. 5b). Remarkably, the ground and heated solids **2**_R and **2**_R2 were amorphous, indicating that the sterically demanding *tert*-butyl groups suppress thermal crystallization processes. The ground solids of **3** and **4** were also amorphous, and the other solids were almost the same crystalline phases (Fig. S5†).

To further investigate the MCL properties of the D–A–D molecules, differential scanning calorimetry (DSC) measurements were performed. Among the solid samples of **1**, only the ground solid **1**_R displayed a glass transition point at 154 °C and a crystallization point at 218 °C, prior to an endothermic process at 293 °C (Fig. 5c). This result suggests that **1**_R is a metastable state and therefore transformed into a more thermodynamically stable state **1**_O2 by heating through the glass transition and crystallization processes. The ground solids of **3** and **4** also showed glass transition and crystallization points, indicating that these solids are also metastable states (Fig. S6†). Regarding the DSC curve of the ground solid **2**_R, a crystallization point was not observed, and only a glass transition point was found at 214 °C, prior to a melting point at 300 °C, indicating that **2**_R does not undergo crystallization through thermal annealing, which is consistent with the amorphous character of solid **2**_R2 (Fig. 5b and d).

### Single crystal X-ray analysis of **1**_O

**Fig. 6:**
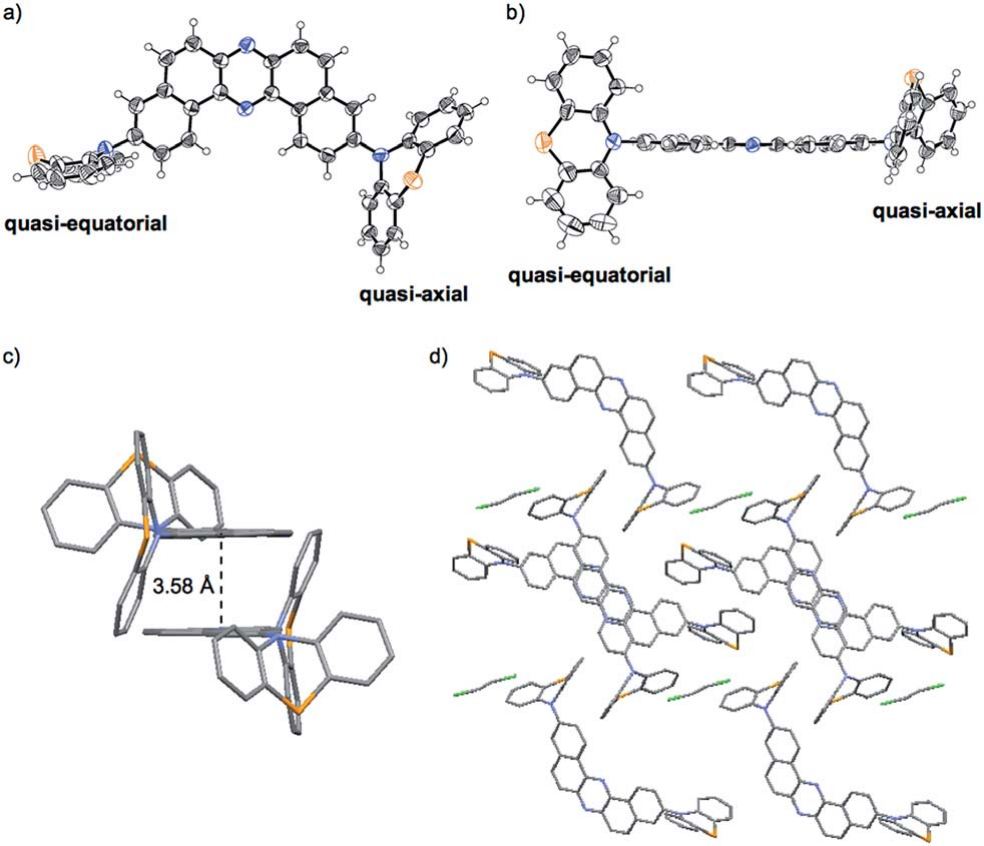
ORTEP drawings of the single crystal of **1**_O: (a) top view and (b) side view (thermal ellipsoids are set at the 50% probability level). (c) A pair of two molecules and (d) the packing structure seen along the *b* axis.

Importantly, X-ray analysis of a single crystal of **1**_O revealed the conformation in the crystal (Fig. 6).^27^ As mentioned above, a PTZ moiety can adopt either “quasi-equatorial” or “quasi-axial” conformations.^22a,23,24^ Regarding the PTZ units in **1**_O, one adopts a “quasi-equatorial” and the other a “quasi-axial” conformation against the DBPHZ core (Fig. 6a and b). In the crystal structure, two molecules make a pair in which two phenazine cores form a slipped stack (π–π interplane distance 3.58 Å), with the PTZ moieties pointing to the opposite sides of each other to cancel out the dipole moment (Fig. 6c). The molecular pairs are packed through close CH···π, CH···N, and CH···S contacts, and the vacant spaces are filled with disordered CH_2_Cl_2_ molecules (Fig. 6d). Thermogravimetric analysis (TGA) also indicated the inclusion of some CH_2_Cl_2_ molecules in the crystal structure of **1**_O (Fig. S18b,† *vide infra*). The solvent exposed samples **1**_YO and **2**_Y also contained CH_2_Cl_2_ molecules (Fig. S18e and i†).

### Steady-state photophysical properties of the solutions

**Fig. 7:**
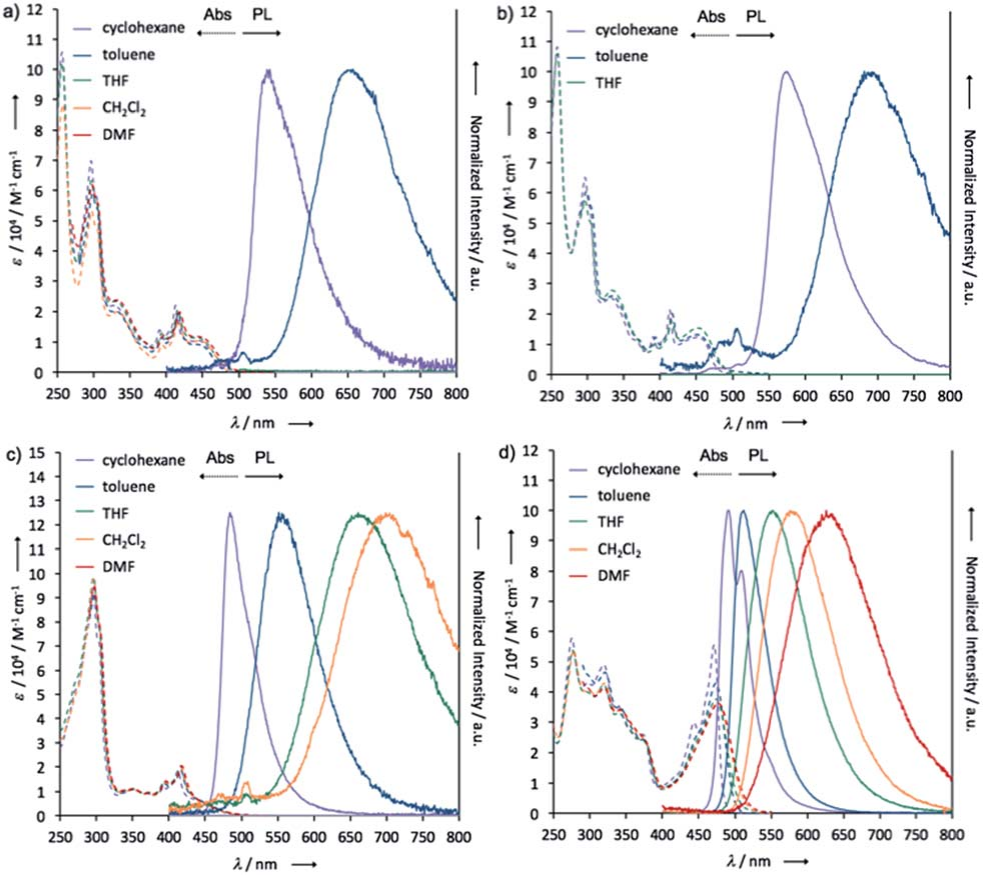
UV-vis absorption and steady-state photoluminescence spectra of dilute solutions of (a) **1**, (b) **2**, (c) **3**, and (d) **4** (concentration: 10^–5^ M).

To investigate the ICT nature of **1–4**, UV-vis absorption and steady-state photoluminescence spectra of their dilute solutions, which were prepared from various solvents, were measured (Fig. 7), and the properties are summarized in Table 1. The maximum absorption wavelengths (*λ*_abs_) and molar absorption coefficients (*ε*) of the solutions of **1** almost did not change in any of the solvents tested (Fig. 7a). The cyclohexane solution of **1** showed green emission from a locally excited (^1^LE) state (*λ*_em_ 543 nm, *Φ*_FL_ 0.06), while the toluene solution emitted red light (*λ*_em_ 657 nm, *Φ*_FL_ 0.07) from ^1^CT, which are very similar to those of POZ–DBPHZ.^21^ In the case of more polar solvents (*e.g.* THF, CH_2_Cl_2_, and DMF), no emission was observed, indicating strong ICT character. It should be noted that the emission profiles (*λ*_em_ and spectra shapes) of crystal **1**_Y (*λ*_em_ 568 nm) and the ground sample **1**_R (*λ*_em_ 673 nm) were almost the same as those of the ^1^LE (*λ*_em_ 543 nm) and ICT (*λ*_em_ 657 nm) states, respectively (Fig. S2†). This would imply that the drastic color-changing MCL in the solid samples of **1** and **2** (*e.g.* from **1**_Y to **1**_R) could be ascribed to the change in emissive states between ^1^LE and ^1^CT states. Likewise, the solution of **2** also exhibited ICT behavior (Fig. 7b). Notably, both *λ*_em_ of **2** were red-shifted, compared with those of **1**, probably due to the electron-donating effect of the *tert*-butyl groups. The carbon-bridged analogue **3** also showed strong ICT character (Fig. 7c), but the degree of red-shift was decreased, compared with **1**, probably due to the less electron-donating nature of the carbon bridging atoms. In polar solvents, a weak emission was observed at around 500 nm for **1**, **2** and **3** (Fig. 7a–c), ascribed to the remnants of emission from the ^1^LE state. The non-bridged D–A–D compound **4** also showed distinct solvatochromism in emission (Fig. 7d). The quantum yields of the solutions of **4** (*Φ*_FL_ 0.34–0.56) were higher than those of the other investigated D–A–D molecules, suggesting that the propeller structures of the diphenyl amino groups suppress molecular motions that would lead to non-radiative decay. A very small red shift (Δ*λ*_em_ ∼ 2 nm) of the emission spectra of **2** in a higher concentration solution (10^–3^ M) (Fig. S9†)^28^ would imply that excimer formation does not significantly contribute to drastic color changes in the solid state such as those observed from **2**_YG to **2**_R (*vide infra*).

**Table 1:** Summary of the photophysical properties of dilute solutions of **1–4** (10^–5^ M)

Compound	Solvent	*λ*_abs_ (nm)	*ε* (M^–1^ cm^–1^)	*λ*_em_ (nm)	*Φ*_FL_ ^*a*^
**1**	*c*-hex	413	22 000	543	0.06
**1**	Toluene	416	19 300	657	0.07
**1**	THF	416	20 500	ND	<0.01
**1**	CH_2_Cl_2_	417	16 800	ND	<0.01
**1**	DMF	418	19 800	ND	<0.01
**2**	*c*-hex	451	12 400	575	0.11
**2**	Toluene	451	13 300	682	0.04
**2**	THF	452	15 300	ND	<0.01
**3**	*c*-hex	413	18 600	484	0.16
**3**	Toluene	416	19 000	552	0.11
**3**	THF	416	20 400	661	0.07
**3**	CH_2_Cl_2_	416	19 800	699	0.05
**3**	DMF	418	20 500	ND	<0.01
**4**	*c*-hex	471	55 500	492	0.56
**4**	Toluene	473	42 900	512	0.44
**4**	THF	474	37 700	552	0.59
**4**	CH_2_Cl_2_	476	36 400	576	0.64
**4**	DMF	477	36 200	626	0.34

^*a*^Determined with an integrating sphere.

To obtain further information about the PTZ–DBPH–PTZ triads, diffuse reflection spectra of solid samples of **1** were measured (Fig. S10†). Notably, each spectrum was different from one another, and the onset wavelengths (*λ*_onset_) were red-shifted in the order of **1**_Y < **1**_YO < **1**_O < **1**_O2 < **1**_R ranging from 516 to 587 nm, which is also consistent with the order of *λ*_em_ (Fig. 4a). These results would indicate that the bandgaps of the solid samples are significantly varied through variation of the PTZ conformations.

### Correlation of MCL behavior with conformers of **1**

**Fig. 8:**
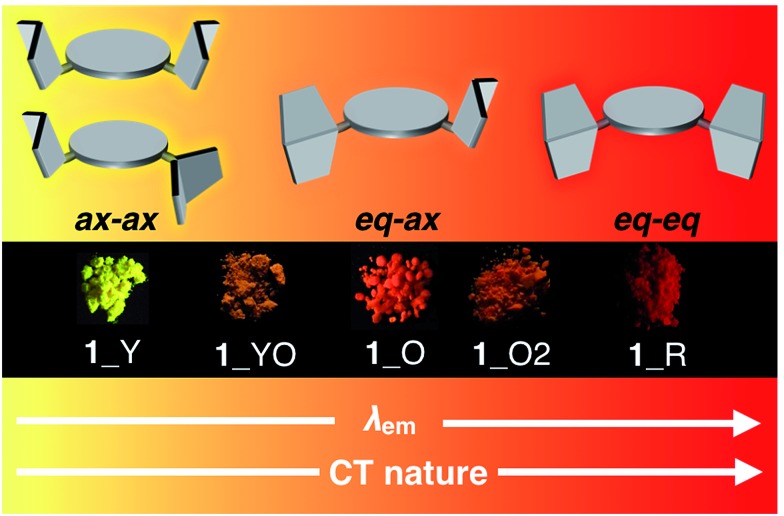
Schematic correlation between emission color and the molecular conformation of **1**.

Taken together, a correlation diagram between emission color and the molecular conformation of **1** is illustrated in Fig. 8. Since only sulfur-bridged D–A–D compounds (**1** and **2**) showed drastic color changes in response to external stimuli, conformational flexibility of the PTZ units should induce distinct MCL character. Based on our previous report, perpendicularly twisted POZ–DBPHZ exhibits red-TADF from an emissive ICT state.^21^ Therefore, red emission from the solid **1**_R should come from the highly twisted eq–eq conformer. The orange emission of **1**_O would be attributed to emission from a weaker ICT excited state, which would be generated from the eq–ax conformer as clearly shown from the crystallographic analysis (Fig. 6a and b). Furthermore, the most high-energy emission of **1**_Y would derive from a radiative process from a ^1^LE state associated with the ax–ax conformer. The bottom line is that the presence of two “conformer-switchable” PTZ units allows for production of various metastable states and thereby realization of multi-color changing MCL properties in the solid state. Also, the inclusion of solvent molecules (*e.g.* from **1**_R to **1**_YO) can cause a change in their packing modes by which the population of a conformer also varies through solvation/de-solvation cycles. This might result in the mixing of different conformers, leading to moderate color changes derived from a mixture of different excited states.

### Investigation of the TADF properties

**Fig. 9:**
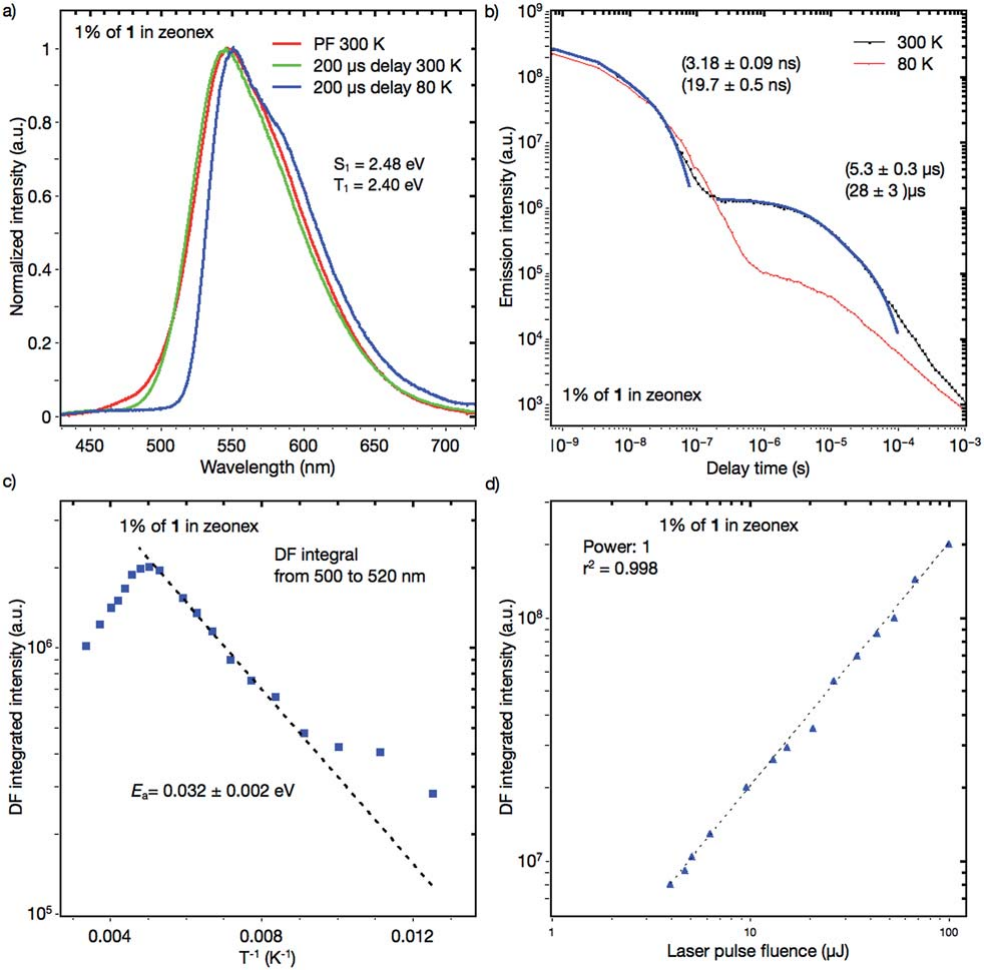
(a) PF, DF and PH spectra of a **1**:Zeonex® blended film; (b) transient decay of a 1 wt% **1**:Zeonex® blended film at 300 K and 80 K; (c) temperature dependence of the delayed fluorescence of a 1 wt% **1**:Zeonex® blended film; and (d) power dependence of delayed fluorescence of a 1 wt% **1**:Zeonex® blended film at 300 K.

**Fig. 10:**
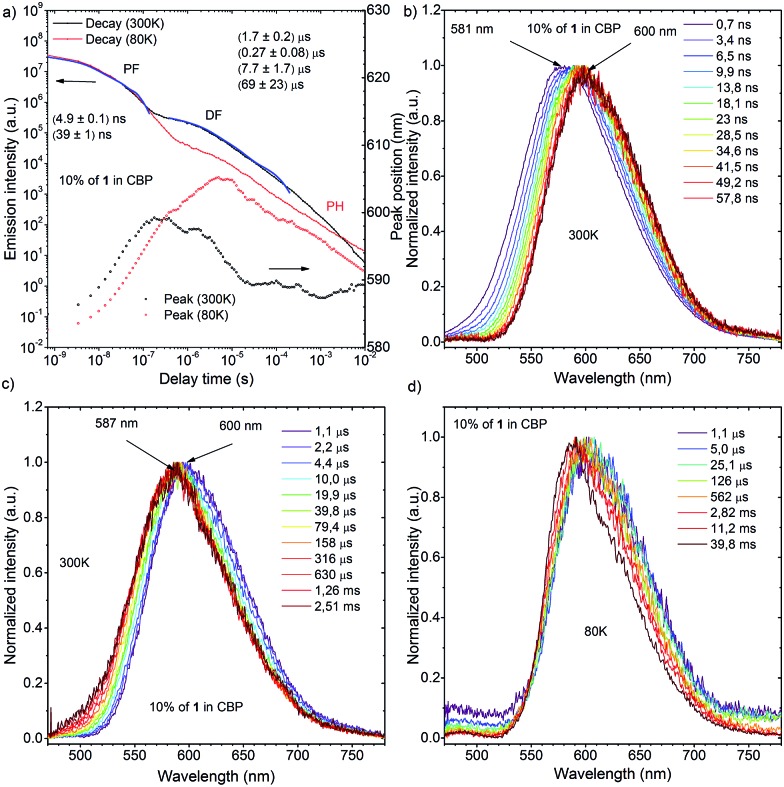
(a) Transient decay and movement of the emission maximum of a 10 wt% **1**:CBP blended film at 300 K and 80 K; time dependence spectra of a 10 wt% **1**:CBP blended film in different time ranges: (b) and (c) are spectra measured at 300 K, while (d) was measured at 80 K.

Dynamic photophysical measurements revealed that all molecules exhibited TADF properties but that the most promising was derivative **1** (Fig. 9, 10, and S11–S17†). In all cases, time-resolved photoluminescence from a blend film (1 wt% of the compounds in Zeonex®) at 300 K showed two components in transient decay: these are attributed to prompt fluorescence (PF) and delayed fluorescence (DF), both of which have the same emission spectra (Fig. 9a and b; S12a and b; S14a and b; S16a and b†). The phosphorescence (PH) which corresponds to the PH of the acceptor unit^21^ was shifted bathochromically when *t*-butyl groups were introduced (from **1** to **2**). This result suggested that compound **2** has a more planar structure, thereby giving rise to increased conjugation. The energy levels of S_1_ and T_1_ for compound **1** were 2.48 and 2.40 eV, respectively, giving a very low Δ*E*_ST_ = 0.08 eV, indicating highly decoupled HOMO/LUMO orbitals on the donor and acceptor units. Notably, the values of S_1_ and T_1_ were the same as those of the oxygen-bridged analogue POZ–DBPHZ as previously reported (Fig. 1),^21^ suggesting that in the Zeonex® film two PTZ units adopt electronically decoupled quasi-equatorial/quasi-equatorial conformations, which are perpendicular to the DBPHZ core. The S_1_ and T_1_ levels for compound **2** were 2.30 and 2.17 eV, respectively, giving Δ*E*_ST_ = 0.13 eV (Fig. S12†) and showing the increase in the Δ*E*_ST_ gap and the bathochromic shift resulting from *tert*-butyl group incorporation onto the PTZ donor group. The S_1_ and T_1_ levels for the acridine derivative **3** were 2.67 and 2.38 eV, respectively, giving Δ*E*_ST_ = 0.29 eV (Fig. S14†). The highest Δ*E*_ST_ was observed for compound **4** as in the previously presented work^21^ (S_1_ = 2.63 eV and T_1_ = 2.33 eV, Δ*E*_ST_ = 0.30 eV). The Arrhenius plot for compound **1** (Fig. 9c) showed a very low delayed fluorescence (DF) activation energy (*E*_a_) of about 0.03 eV, close to Δ*E*_ST_ (0.08 eV), which was determined by time-resolved photoluminescence, indicating that **1** is an excellent candidate as a TADF emitter like POZ–DBPHZ.^21^ The maximum DF intensity was observed at about 200 K, and above this temperature DF intensity started to decrease (Fig. 9c). In the case of **1**, three important processes of relaxation of the excited state can be proposed along with prompt ^1^CT (S_1_) decay: (1) a radiative transition T_1_ → S_0_ (phosphorescence); (2) the T_1_ → S_1_ transition (rISC) and then the radiative transition S_1_ → S_0_ (TADF); (3) the non-radiative relaxation T_1_ → S_0_. When the temperature is increased, the rate constant of process 2 would increase to give strong TADF emission. However, over 200 K, process 3 starts to dominate and the DF intensity decreases. At 80 K, DF can still be observed and pure PH (process 1) was observed at very late delay times (Fig. 9b). This feature would indicate that there was still enough vibrational energy for some molecules to overcome the S_1_–T_1_ energy gap at this temperature in order to form the mixed ^3^LE–^3^CT state required for spin orbit coupling (SOC) to occur.^16^ A linear power dependence of the delayed emission intensity with laser pulse fluence for **1** doped (1 wt%) in Zeonex® at 300 K confirms that the observed DF component is TADF (Fig. 9d).

Time-resolved photoluminescence of a 10 wt% **1**:CBP blend film indicated a far more complex situation due to the inhomogeneous environment of **1** when evaporated in the host material (Fig. 10a). Analysis of the spectra at 300 K showed contributions of several excited states in the total photoluminescence. At 0.7 ns, PF from the CBP blend film had the same onset as that from the Zeonex® blend film at 500 nm (Fig. 10b). However, the emission peak *λ*_em_ from the CBP blend film was red-shifted (581 nm) compared to that observed in Zeonex® (546 nm), and the spectrum was definitely structureless (Fig. 10b). When the delay time increased, the emission spectrum monotonically red-shifted, and the largest *λ*_em_ (600 nm) was observed at 57.8 ns (Fig. 10b). From 57.8 ns to 1.1 μs, *λ*_em_ plateaued at about 600 nm, but then from 1.1 μs to 2.5 ms, *λ*_em_ slowly blue-shifted back to 587 nm and stayed at this value (Fig. 10c). It is concluded that PF at very early delay times includes a small contribution from ^1^LE emission of **1**, although ^1^CT emission dominates. The monotonic red-shift over the first 60 ns is indicative of an inhomogeneous system where the bluest ^1^CT states decay first and the redder states decay at longer times, to give the impression of time dependent relaxation.^15*c*^ The emission observed from 57.8 ns to 1.1 μs at 600 nm could indicate emission from an excimer species of **1** (Fig. 10c). Formation of excimers is normal in highly concentrated samples. After 1.1 μs, ^1^CT emission from isolated **1** molecules starts to dominate (*λ*_em_ = 587 nm) and shows strong temperature dependence in line with TADF. The small difference in *λ*_em_ between the ^1^CT emission and the excimer emission (Δ*λ*_em_ 13 nm) suggests that the drastic change in the emission color from yellow (568 nm) to red (673 nm) (Δ*λ*_em_ 105 nm) in the fluorochromism would be derived from switching of the ^1^LE and ICT excited states, not from excimer formation. Fitting of the DF transient decay measured at 300 K gave multiexponential decay components with more than four time constants, therefore, only a part of the whole decay was fitted using four exponents. Such a multiexponential decay transient is indicative of an inhomogeneous system, along with mixing of ^1^CT and excimer delayed emission. Behavior of the **1**-doped (10 wt%) CBP blend film at 80 K was also complicated (Fig. 10d). It seems that ^1^CT emission decreased significantly and became negligible, therefore the excimer emission spectrum appears to red-shift further to *λ*_em_ = 605 nm at 6 ms delay. After 6 ms, another peak component appears and *λ*_em_ gradually blue-shifts. The spectrum recorded 60 ms after excitation clearly showed vibrational structure and the triplet energy level was 2.29 eV. Isolated **1** molecules are expected to have the same triplet energy as in Zeonex® (T_1_ = 2.40 eV). Therefore, the observed DF at 60 ms at 80 K is ascribed to ^3^LE phosphorescence of the **1** excimer (Fig. S11†). Linear laser power dependence of the DF from a 10 wt% **1**:CBP blend film at 300 K indicated clear TADF character (Fig. S11†).

**Fig. 11:**
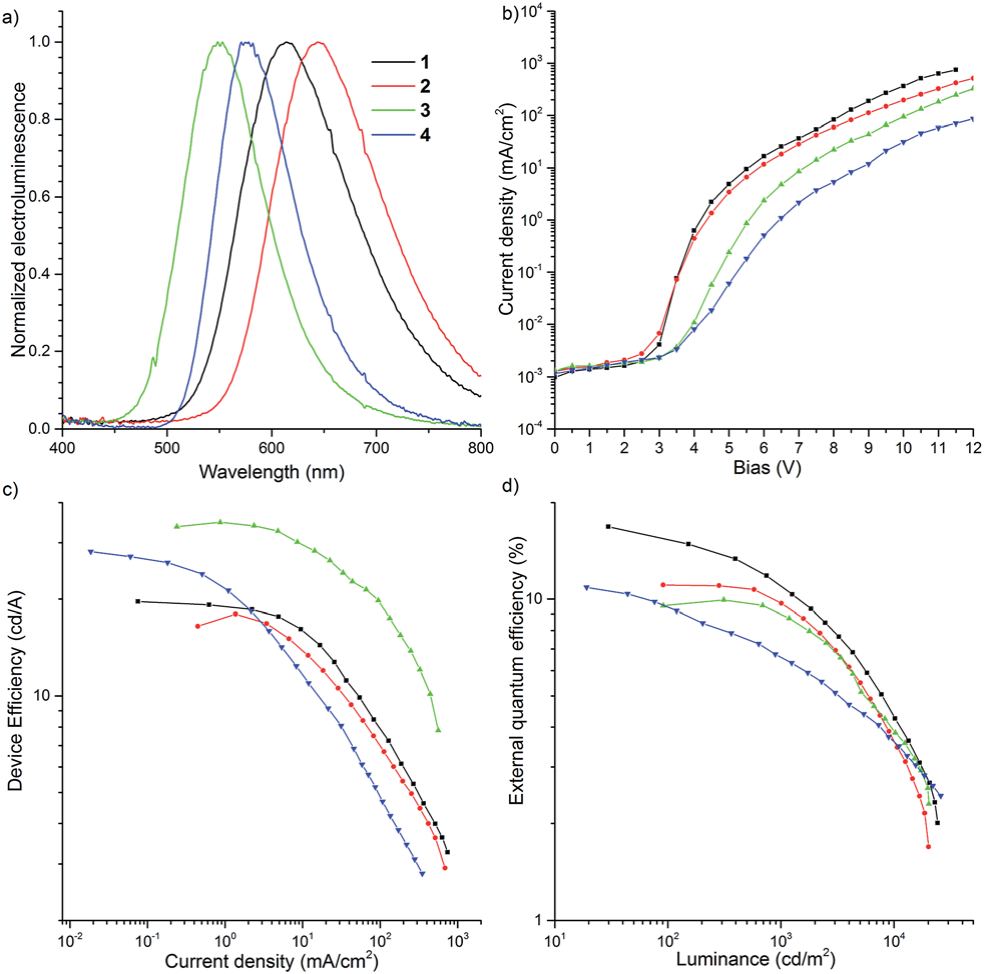
Characterization of OLED devices.

All four materials were analyzed for TADF emission, and derivative **2** showed similar results to the unsubstituted derivative **1**, but the emission was shifted by *ca.* 30 nm to the red region, and the overall DF contribution was lower (Fig. S12 and S13†), resulting in lower device efficiency (Fig. 11, *vide infra*). Acridine derivative **3** exhibited some TADF behavior, but the Δ*E*_ST_ was much larger than those seen for the other derivatives which resulted in a small TADF contribution (Fig. S14 and S15†) and the lowest device efficiency in this novel series of compounds (Fig. 11d, *vide infra*).

### Fabrication of TADF-based OLED devices

To investigate the possibility of applying the D–A–D compounds **1–4** as OLED emitters, thermogravimetric analysis (TGA) of the recrystallized, ground, heated and solvent treated samples was performed (Fig. S18†). High thermal decomposition temperatures [*T*_d_ (5 wt% loss) 439–450 °C for **1**, 455–459 °C for **2**, 428–434 °C for **3**, and 403–424 °C for **4**] indicated that these molecules would be applicable to vacuum thermal deposition for purification and fabrication of OLEDs. Cyclic voltammetry (CV) showed that the D–A–D molecules **1** and **2** exhibited reversible redox curves, indicating their high electrochemical stability (Fig. S19†). Regarding the molecules **3** and **4**, when a negative voltage was applied, a reversible redox couple was clearly observed, however it was quasi-reversible in the presence of a positive voltage (Fig. S19c and d†). The CV curves of **1–4** in the positive and negative side are attributed to the redox processes of the donors and acceptor, respectively. The IP/EA energy levels were determined by CV experiments: –5.33/–3.38 eV (**1**); –5.20/–3.35 eV (**2**); –5.55/–3.40 eV (**3**); –5.59/–3.29 eV (**4**). The energy levels of DBPHZ **1** are almost the same as POZ–DBPHZ (–5.36/–3.38 eV).^21^ The HOMO levels of these D–A–D compounds decreased in descending order of the electron-donating nature of the donors (**2** > **1** > POZ–DBPHZ > **3** > **4**). However, the LUMO levels were different. The energy level of **4** was the highest and those of the other molecules decreased in the same order as the HOMO levels (**4** > **2** > **1** = POZ–DBPHZ > **3**), suggesting that the HOMOs and LUMOs of DBPHZs **1–3** and POZ–DBPHZ were completely separated due to their perpendicular structures, although some HOMO/LUMO interactions would exist for DBPHZ **4**, because of the propeller structures of the diphenyl amino groups.

OLED devices were fabricated using the co-evaporation technique (Fig. 11). The device structure used was as follows: ITO/NPB (40 nm)/10 wt% D–A–D compound (**1–4**) in CBP (30 nm)/TPBi (50 nm)/LiF (1 nm)/Al (100 nm). The device based on **1** exhibited orange emission at 613 nm with up to 16.8% EQE, which greatly surpasses the theoretical maximum (5%) of conventional fluorescent materials, and 19.6 cd A^–1^ maximum efficiency (Fig. 11a and c). The roll-off process was observed above 7 V and the device was characterized by high luminance (>25 000 cd) (Fig. 11b and d).

## Conclusions

We have succeeded in developing novel D–A–D molecules **1** and **2** that exhibit both distinct multi-color-changing MCL and efficient TADF properties through the combination of an electron deficient DBPHZ acceptor and “two-conformation-switchable” and electron-donating phenothiazine donors. The quantum chemical calculations using the DFT method indicated the existence of four possible thermodynamically interconvertible conformers of **1** derived from conformational alteration of the phenothiazine units. Compared with DBPHZ derivatives substituted with the other donor units, the necessity of the sulfur-bridge in the donor unit for drastic multi-color-changing luminochromism was demonstrated. Notably, single crystal X-ray analysis showed the asymmetric molecular structure of **1**_O having two conformational types of phenothiazines: one is “quasi-equatorial” and the other “quasi-axial”. Most importantly, we propose that the high-contrast luminochromic behavior would result in switching of the LE and ICT excited states caused by conformational change of the phenothiazine units. Furthermore, **1** had a very small Δ*E*_ST_ induced by efficient HOMO/LUMO decoupling and exhibited strong TADF. Based on **1**, TADF OLED devices were fabricated and gave orange emission with high light emitting efficiency, 16.8% EQE. The findings reported here will further guide us to a novel design principle for the construction of multi-functional materials in the future. However, the effects of MCL characteristics on their TADF properties (as pure states and as dispersed forms in device structures), and *vice versa*, have remained a riddle. The research to clarify the relationship between both properties is currently being investigated in our group and will be reported in due course.
