# Supplementary material for: Thermally activated delayed fluorescent phenothiazine–dibenzo[a,j]phenazine–phenothiazine triads exhibiting tricolor-changing mechanochromic luminescence
Source: Chem Sci. 2017 Jan 11;8(4):2677–86. doi: 10.1039/c6sc04863c (PMC5433494; doi:10.1039/c6sc04863c)
Supplement: Supplementary file 1 [file SC-008-C6SC04863C-s001.pdf]

Electronic Supplementary Material (ESI) for Chemical Science.
This journal is © The Royal Society of Chemistry 2017

Electronic Supplementary Information (ESI) for

Thermally Activated Delayed Fluorescent Phenothiazine-Dibenzo[*a*,*j*]phenazine-Phenothiazine Triads Exhibiting Tricolor-Changing Mechanochromic Luminescence

Masato Okazaki,[†] Youhei Takeda,*[,†] Przemyslaw Data,*[,‡,§] Piotr Pander,[‡,§] Heather Higginbotham,[‡] Andrew P. Monkman,[‡] and Satoshi Minakata*[,†]

[†]*Department of Applied Chemistry, Graduate School of Engineering, Osaka University, Yamadaoka 2-1, Suita, Osaka 565-0871, Japan*
[‡]*Physics Department, Durham University, South Road, Durham DH1 3LE, United Kingdom*
[§]*Faculty of Chemistry, Silesian University of Technology, M. Strzody 9, 44-100 Gliwice, Poland*

E-mail: takeda@chem.eng.osaka-u.ac.jp; przemyslaw.data@durham.ac.uk; minakata@chem.eng.osaka-u.ac.jp

Table of Contents

General Remarks	
Materials	S2
Synthetic Procedures and Spectroscopic Data of **1**–**4**	S2–S3
Mechanochromic Luminescence Properties	S3–S6
Acid-induced Emission Quenching	S7–S8
Powder X-Ray Diffraction (PXRD) Analysis	S9
Differential Scanning Calorimetry (DSC) Analysis	S10
Single Crystal X-ray Crystallographic Data	S10–S11
UV-vis Absorption and Emission Spectra	S12–S13
Concentration Effect on Emission Properties	S14
Diffuse Reflection Spectra	S15
Theoretical Calculations	S15
Time-resolved Photoluminescence Properties	S16–S32
Thermogravimetric Analysis (TGA)	S33–39
Cyclic Voltammograms (CV)	S40–S42
NMR Charts	S43
References	S44–S47
	S48

General Remarks. All reactions were carried out under an atmosphere of nitrogen unless otherwise noted. Melting points were determined on a Stanford Research Systems MPA100 OptiMelt Automated Melting Point System. ^{1}H and ^{13}C NMR spectra were recorded on a JEOL JMTC-400/54/SS spectrometer (^{1}H NMR, 400 MHz; ^{13}C NMR, 100 MHz) using tetramethylsilane as an internal standard. Infrared spectra were acquired on a SHIMADZU IRAffinity-1 FT-IR Spectrometer. Mass spectra were obtained on a JEOL JMS-DX303HF mass spectrometer. High-resolution mass spectra (HRMS) were obtained on a JEOL JMS-DX303HF mass spectrometer. UV-vis spectra were recorded on a Shimadzu UV-2550 spectrophotometer. Emission spectra were recorded on a HAMAMATSU C11347-01 spectrometer with an integrating sphere. Differential scanning calorimetry (DSC) measurements were carried out on a DSC 6220 (SII) system at a scanning rate of 10 °C min^{-1} under a N_2 atmosphere. Powder X-ray diffraction (XRD) patterns were recorded by Rigaku SmartLab X-ray diffractometer with CuKα radiation (λ = 1.5418 Å). Diffuse reflection spectra were collected by a JASCO V-670 UV-Vis-NIR Spectrophotometer. Cyclic voltammetry (CV) was performed with ALS-600 (BAS Inc.) system. Thermogravimetric analysis (TGA) was performed with TG/DTA-7200 (SII) system. Products were purified by chromatography on silica gel BW-300 and Chromatorex NH (Fuji Silysia Chemical Ltd.). Analytical thin-layer chromatography (TLC) was performed on pre-coated silica gel glass plates (Merck silica gel 60 F254 and Fuji Silysia Chromatorex NH, 0.25 mm thickness). Compounds were visualized with UV lamp. Small molecules and cathode layers were thermally evaporated using Kurt J. Lesker Spectros II Deposition at 10^{-6} mbar. All organic materials and aluminum were deposited at a rate of 1 $Ås^{-1}$, and the LiF layer was deposited at 0.1 $Ås^{-1}$. Investigation of the characteristic of OLED devices was conducted in 10 inches integrating sphere (Labsphere) connected to a SourceMeter unit. Phosphorescence (PH), prompt fluorescence (PF), and delayed fluorescence (DF) spectra and decays were recorded using nanosecond gated luminescence and lifetime measurements (from 400 ps to 1 s) using either a high energy pulsed Nd:YAG laser emitting at 355 nm (EKSPLA) or a N_2 laser emitting at 337 nm. Emission was focused onto a spectrograph and detected on a sensitive gated iCCD camera (Stanford Computer Optics) having sub-nanosecond resolution. PF/DF time resolved measurements were performed by exponentially increasing gate and delay times.

Materials. 6,6'-Dibromo-1,1'-binaphthalene-2,2'-diamine (**S1**) [CAS No. 861890-12-2] was prepared from 6-Bromo-2-naphthol [CAS No. 15231-91-1] according to the procedures in literature.[S1] 6-Bromo-2-naphthol was prepared according to the procedures in literature.[S2] 3,7-di-*tert*-butyl-10*H*-phenothiazine [CAS No. 27075-55-4][S3] and 9,9-dimethyl-9,10-dihydroacridine [CAS No. 6267-02-3][S4] were prepared according to the procedures in literature. Tetrabutylammonium hexafluorophosphate for electrochemical measurement was purchased from TCI and used after repeated (3 times) recrystallization from EtOH. Commercial reagents were purchased from Sigma-Aldrich, TCI, or Wako Pure Chemical Industries, Ltd. and used as received. MeOH was dried over activated molecular sieves 3Å. Toluene was purchased as

dehydrated grade and purified by passing through a solvent purification system (KOREA KIYON Co., Ltd.). Solvents of fluorescence spectroscopic grade were purchased from Nacalai Tesque Inc. and Kanto Chemical Co., Inc. for measurement of UV-vis and emission spectra. All organic evaporated compounds were purified by Creaphys organic sublimation system, and the following compounds were purchased from companies: CBP [4,4′-bis(*N*-carbazolyl)-1,1′-biphenyl] (Sigma Aldrich), NPB [*N,N′*-Di-1-naphthyl-*N,N′*-diphenylbenzidine] (TCI-Europe), TPBi [2,2',2"-(1,3,5-Benzinetriyl)-tris(1-phenyl-1-H-benzimidazole)] (LUMTEC), LiF (99.995%, Sigma Aldrich), and Aluminium wire (99.9995%, Alfa Aesar). OLED devices were fabricated using pre-cleaned indium-tin-oxide (ITO) coated glass substrates purchased from Ossila with a sheet resistance of 20 Ω/cm^2 and ITO thickness of 100 nm. The formed OLED devices had a pixel size of 2 mm by 1.5 mm.

Synthetic Procedures and Spectroscopic Data of 1–4.

Preparation of 3,11-Dibromodibenzo[a,j]phenazine.

3,11-Dibromodibenzo[*a,j*]phenazine (**S2**) was prepared through a slightly modified method developed by our group[S5] as follows (Eq S1): To a two-necked round-bottomed flask (300 mL) equipped with a three-way stopcock and a magnetic stir bar, was added 6,6’-dibromo-1,1’-binaphthalene-2,2’-diamine (**S1**) (442.1 mg, 1.0 mmol) under the air. The vessel was capped with a rubber septum, evacuated, and refilled with N_2 gas for 3 times, and then MeOH (100 mL) was added through the septum. To this stirred mixture, was added 1,3-diiodo-5,5-dimethylhydantoin (DIH) (3.04 g, 8.0 mmol) under a stream of N_2 gas at room temperature. The resulting solution was stirred for 3 h before quenched with aqueous $Na_2S_2O_3$ (1.0 M, 100 mL), and the resulting mixture was extracted with CH_2Cl_2 (100 mL × 3). The combined organic extracts were dried over Na_2SO_4 and concentrated under vacuum to give the crude product, which was purified by flash column chromatography (eluent: hexane/EtOAc 95:5–9:1) on silica gel to give product **S2** (219.0 mg, 50%). Further purification was carried out by recrystallization from $CHCl_3$. The spectroscopic data were identical with those reported in literature.[S5]

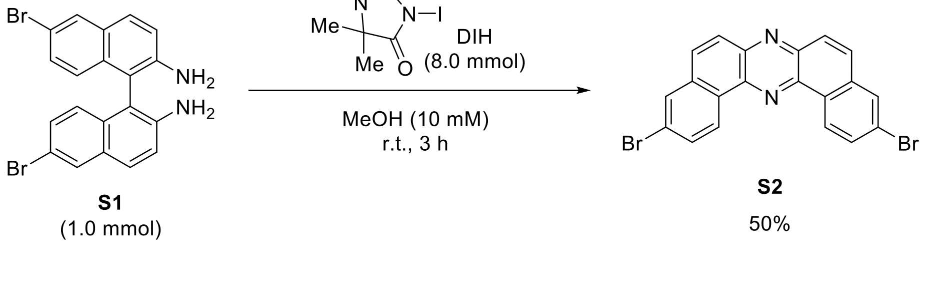

(S1)

Typical Procedures for the Synthesis of 3,11-Diaminodibenzo[a,j]phenazines

3,11-Diaminodibenzo[*a,j*]phenazines **1**–**4** were prepared through a slightly modified Pd-catalyzed amination reaction[S6] of **S2** with aromatic amines as follows (*Methods A, B or B'*).

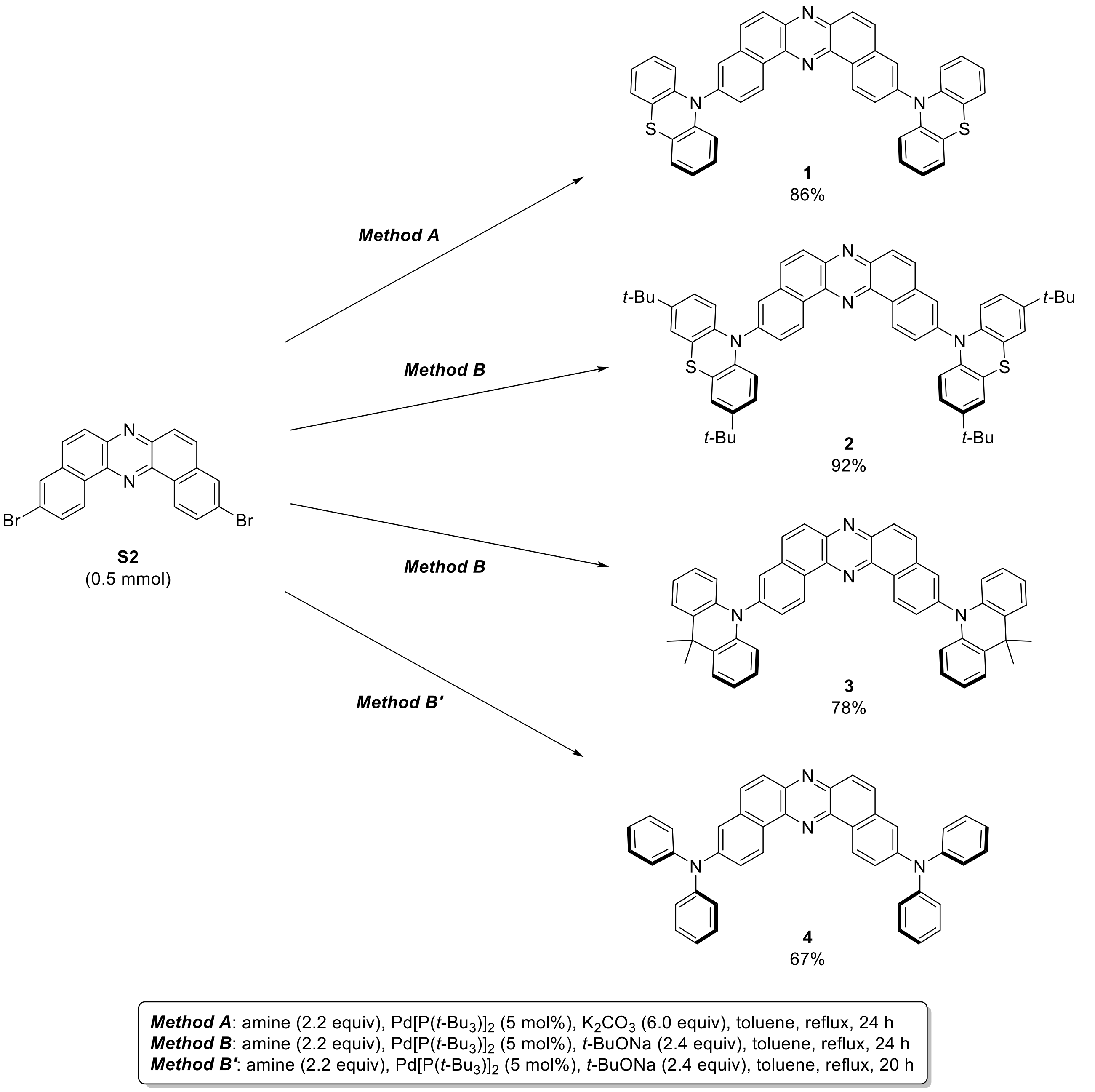

Method A: Toluene was degassed through freeze-pump-thaw cycling for 3 times before used. In a glovebox, to a two-necked reaction tube (10 mL) equipped with a three-way stopcock and a magnetic stir bar, was added Pd[P(*t*-Bu)$_3$]$_2$ (12.8 mg, 5 mol%), and the tube was closed with a rubber septum. Outside the glovebox, dibromophenazine **S2** (219.0 mg, 0.50 mmol), 10*H*-phenothiazine (219.1 mg, 1.10 mmol), K_2CO_3 (414.5 mg, 3.0 mmol), and toluene (5 mL) were added under a stream of N_2 gas at room temperature, and the resulting mixture was stirred under reflux for 24 h. Water (5 mL) was

added to the reaction mixture, and the organic layer was extracted with CH_2Cl_2 (20 mL × 3). The combined organic extracts were dried over Na_2SO_4, and the solvent was evaporated in vacuo to give the crude product, which was purified by flash column chromatography (for the detailed purification methods, see the prodcut data).

3,11-Di(10*H*-phenothiazin-10-yl)dibenzo[*a*,*j*]phenazine (1)

Purified by flash column chromatography on NH silica gel (eluent: *n*-hexane/CH_2Cl_2 95:5–8:2) followed by recrystallization from a two-phase solvent of *n*-hexane/$CHCl_3$ (3:1); Yellow solid (290.2 mg, 86%); mp 300 °C (dec.); R_f 0.18 (*n*-hexane/CH_2Cl_2 8:2, NH silica); ^{1}H NMR (400 MHz, $CDCl_3$): δ 6.56 (dd, J = 1.6, 7.6 Hz, 4H), 6.92–7.00 (m, 8H), 7.18 (dd, J = 2.0, 7.2 Hz, 4H), 7.85 (dd, J = 2.0, 8.4 Hz, 2H), 7.97 (d, J = 2.0 Hz, 2H), 8.09 (d, J = 9.2 Hz, 2H), 8.14 (d, J = 9.2 Hz, 2H), 9.77 (d, J = 8.4 Hz, 2H); ^{13}C NMR (100 MHz, $CDCl_3$): δ 118.3, 123.1, 123.4, 126.5, 127.0, 127.3, 127.7, 127.8, 129.7, 132.1, 135.3, 140.5, 142.8, 143.0, 143.7 (One carbon was not detected, probably due to the overlap of the signal with other aromatic peaks); IR (ATR): ν 3039, 1583, 1462, 1352, 1300, 1257, 1145, 850, 754, 740 cm^{-1}; MS (FAB): *m/z* (relative intensity, %) 675 ($[M + H]^+$, 6), 674 (M^+, 7); HRMS (FAB): *m/z* calcd for $C_{44}H_{26}N_4S_2$ (M^+) 674.1599, found 674.1595.

Method B and B': Toluene was degassed through freeze-pump-thaw cycling for 3 times before used. In a glovebox, to a two-necked reaction tube (10 mL) equipped with a three-way stopcock and a magnetic stir bar, were added Pd[P(*t*-Bu)$_3$]$_2$ (12.8 mg, 5 mol%) and sodium *tert*-butoxide (115.3 mg, 1.20 mmol), and the tube was closed with a rubber septum. Outside the glovebox, dibromophenazine **S2** (219.0 mg, 0.50 mmol), amine [3,7-di-*tert*-butyl-10*H*-phenothiazine (342.6 mg), 9,9-dimethyl-9,10-dihydroacridine (230.2 mg), or diphenylamine (186.1 mg)] (1.10 mmol) and toluene (5 mL) were added under a stream of N_2 gas at room temperature, and the resulting mixture was stirred under reflux for 24 h (***Method B***) or 20 h (***Method B'***). Water (5 mL) was added to the reaction mixture, and the organic layer was extracted with CH_2Cl_2 (20 mL × 3). The combined organic extracts were dried over Na_2SO_4, and the solvent was evaporated in vacuo to give the crude product, which was purified by flash column chromatography (for the detailed purification methods, see each prodcut data).

3,11-Bis(3,7-di-*tert*-butyl-10*H*-phenothiazin-10-yl)dibenzo[*a*,*j*]phenazine (2)

Purified by flash column chromatography on NH silica gel (eluent: *n*-hexane/CH_2Cl_2 95:5–9:1) followed by recrystallization from a two-phase solvent of *n*-hexane/$CHCl_3$ (20:1); Yellow solid (413.7 mg, 92%); mp 263 °C; R_f 0.20 (*n*-hexane/CH_2Cl_2 8:2, NH silica); ^{1}H NMR (400 MHz, $CDCl_3$): δ 1.28 (s, 36H), 6.61 (d, J = 8.8 Hz, 4H), 7.02 (dd, J = 2.0, 8.8 Hz, 4H), 7.24 (d, J = 2.0 Hz, 4H), 7.80 (dd, J = 2.0, 8.4 Hz, 2H), 7.89 (d, J = 2.0 Hz, 2H), 8.04 (d, J = 9.2 Hz, 2H), 8.10 (d, J = 9.2 Hz, 2H), 9.70 (d, J = 8.4 Hz, 2H); ^{13}C NMR (100 MHz, $CDCl_3$): δ 31.3, 34.2, 118.8, 123.9, 123.9, 124.5, 124.7, 126.5, 127.5, 127.7, 129.0, 132.1, 135.2, 140.6, 141.1, 142.6, 143.7, 146.8; IR (ATR):

ν 2953, 1595, 1475, 1357, 1305, 1263, 1143, 856, 808, 721 cm^{-1}; MS (FAB): *m/z* (relative intensity, %) 899 ([M + H]$^+$, 100), 898 (M$^+$, 96); HRMS (FAB): *m/z* calcd for $C_{60}H_{58}N_4S_2$ (M$^+$) 898.4103, found 898.4096.

3,11-Bis(9,9-dimethylacridin-10(9*H*)-yl)dibenzo[*a,j*]phenazine (3)

Purified by flash column chromatography on NH silica gel (eluent: *n*-hexane/CH_2Cl_2 95:5–9:1) followed by recrystallization from a two-phase solvent of *n*-hexane/$CHCl_3$ (5:1); Yellow solid (271.0 mg, 78%); mp 315 °C (dec.); R_f 0.33 (*n*-hexane/CH_2Cl_2 8:2, NH silica); ^{1}H NMR (400 MHz, $CDCl_3$): δ 1.78 (s, 12H), 6.35–6.37 (m, 4H), 6.97–6.99 (m, 8H), 7.52–7.54 (m, 4H), 7.86 (dd, J = 2.0, 8.4 Hz, 2H), 8.05 (d, J = 2.0 Hz, 2H), 8.16 (d, J = 9.6 Hz, 2H), 8.21 (d, J = 9.6 Hz, 2H), 9.92 (d, J = 8.4 Hz, 2H); ^{13}C NMR (100 MHz, $CDCl_3$): δ 31.4, 36.1, 114.2, 120.9, 125.4, 126.4, 127.8, 128.3, 130.2, 130.6, 130.9, 130.9, 132.3, 135.5, 140.5, 140.9, 142.4, 143.2; IR (ATR): ν 3035, 2958, 1591, 1473, 1444, 1330, 1271, 931, 856, 796, 740 cm^{-1}; MS (EI): *m/z* (relative intensity, %) 694 (M$^+$, 12), 679 ([M–Me]$^+$, 100), 471 ([$C_{34}H_{21}N_3$]$^+$, 44), 332 ([$C_{24}H_{16}N_2$]$^+$, 81); HRMS (EI): *m/z* calcd for $C_{50}H_{38}N_4$ (M$^+$) 694.3096, found 694.3099.

3,11-Bis(diphenylamino)dibenzo[*a,j*]phenazine (4)

Purified by flash column chromatography on NH silica gel (eluent: *n*-hexane/EtOAc 99:1–98:2) followed by recrystallization from a two-phase solvent of *n*-hexane/$CHCl_3$ (5:1); Orange solid (205.9 mg, 67%); mp 296 °C (dec.); R_f 0.25 (*n*-hexane/EtOAc 8:2, NH silica); ^{1}H NMR (400 MHz, $CDCl_3$): δ 7.13 (t, J = 7.2 Hz, 4H), 7.23 (d, J = 7.6 Hz, 8H), 7.34 (dd, J = 7.2, 7.6 Hz, 8H), 7.51 (d, J = 2.4 Hz, 2H), 7.55 (dd, J = 2.4, 8.8 Hz, 2H), 7.80 (d, J = 8.8 Hz, 2H), 7.94 (d, J = 8.8 Hz, 2H), 9.36 (d, J = 8.8 Hz, 2H); ^{13}C NMR (100 MHz, $CDCl_3$): δ 119.6, 123.2, 123.8, 125.2, 125.8, 126.2, 127.4, 129.5, 131.5, 134.6, 140.9, 141.7, 147.4, 149.0; IR (ATR): ν 3030, 1590, 1476, 1358, 1275, 1141, 870, 848, 790, 750 cm^{-1}; MS (EI): *m/z* (relative intensity, %) 614 (M$^+$, 100), 307 ([$C_{22}H_{15}N_2$]$^+$, 26); HRMS (EI): *m/z* calcd for $C_{44}H_{30}N_4$ (M$^+$) 614.2470, found 614.2469.

Mechanochromic Luminescence (MCL) Properties

Table S1. Summary of MCL properties of **1**–**4** and POZ-DBPHZ.

Sample	λ_{ex} (nm)	λ_{em} (nm)	Φ_{FL}	Sample	λ_{ex} (nm)	λ_{em} (nm)	Φ_{FL}
**1**_Y	300	568	0.16	**3**_Y1	300	592	0.17
**1**_O	400	640	0.08	**3**_Y2	300	577	0.15
**1**_R	400	673	0.12	**3**_Y3	300	562	0.10
**1**_O2	400	646	0.11	**3**_Y4	300	559	0.08
**1**_YO	400	596	0.05	**4**_O1	300	594	0.07
**2**_YG	300	547	0.13	**4**_O2	300	597	0.20
**2**_R	300	663	0.15	**4**_O3	300	600	0.20
**2**_R2	400	675	0.12	**4**_O4	300	599	0.22
**2**_Y	300	564	0.15	POZ-DBPHZ_O	400	633	0.06
				POZ-DBPHZ_R	400	648	0.08

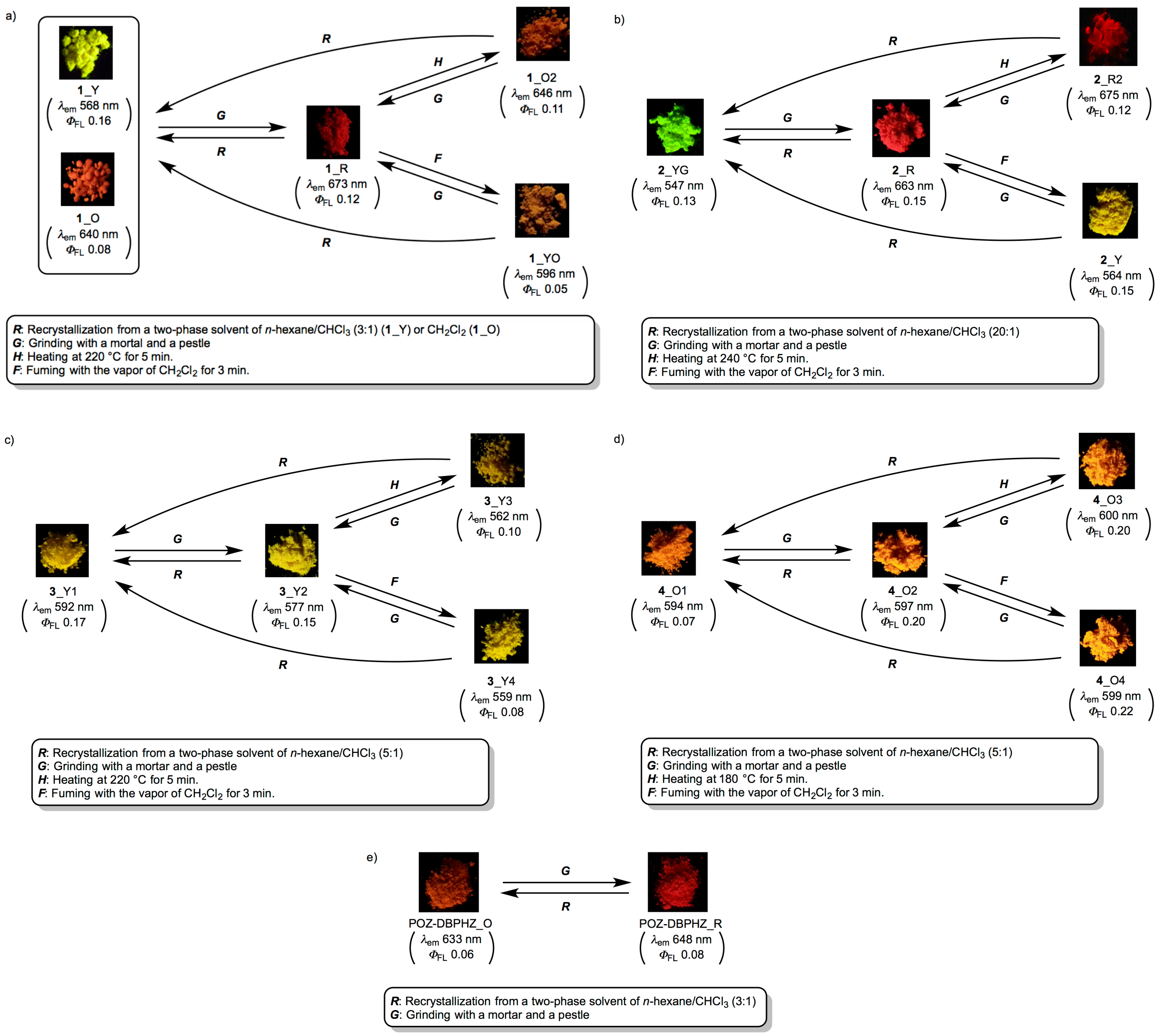

Figure S1. MCL properties of **1**–**4** and POZ-DBPHZ.

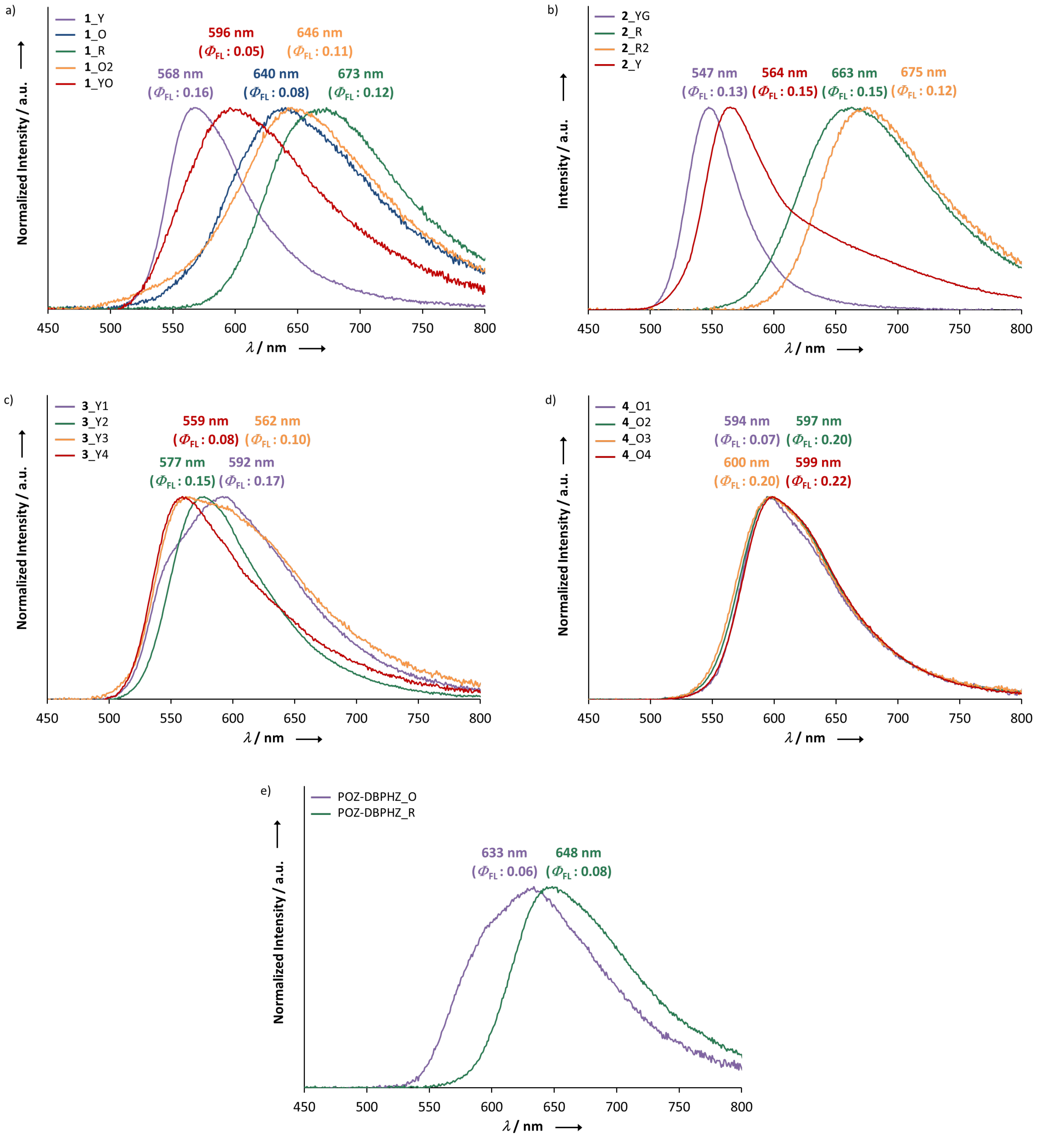

Figure S2. Emission spectra of solid samples of **1**–**4** and POZ-DBPHZ.

Acid-induced Emission Quenching

Table S2. Summary of the properties of acid/base-vapor-treated solids **1**–**4**.

Sample	λ_{ex} (nm)	λ_{em} (nm)	Φ_{FL}
**1**_R	400	673	0.12
**1**_TFA	300	ND	<0.01
**1**_TFA_TEA	300	601	0.05
**1**_TFA_TEA_TFA	300	ND	<0.01
**2**_R	300	663	0.15
**2**_TFA	300	ND	<0.01
**2**_TFA_TEA	300	562	0.06
**2**_TFA_TEA_TFA	300	ND	<0.01
**3**_Y2	300	577	0.15
**3**_TFA	300	ND	<0.01
**3**_TFA_TEA	300	596	0.17
**3**_TFA_TEA_TFA	300	ND	<0.01
**4**_O2	300	597	0.20
**4**_TFA	300	ND	<0.01
**4**_TFA_TEA	300	602	0.05
**4**_TFA_TEA_TFA	300	ND	<0.01

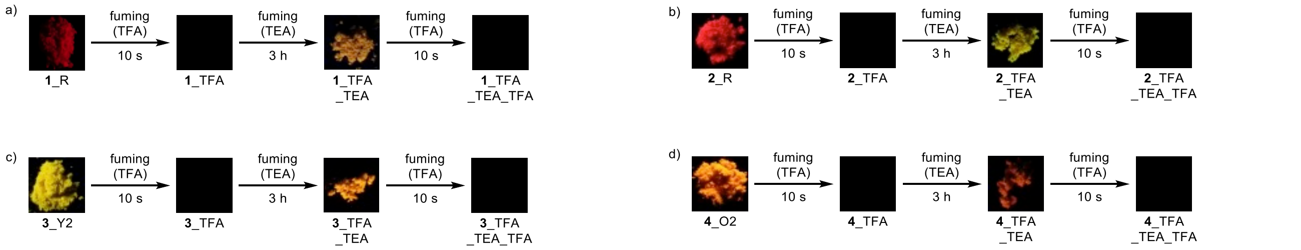

Figure S3. Emission "turn-off/on" cycles induced by the treatment of **1**–**4** with acid/based vapor.

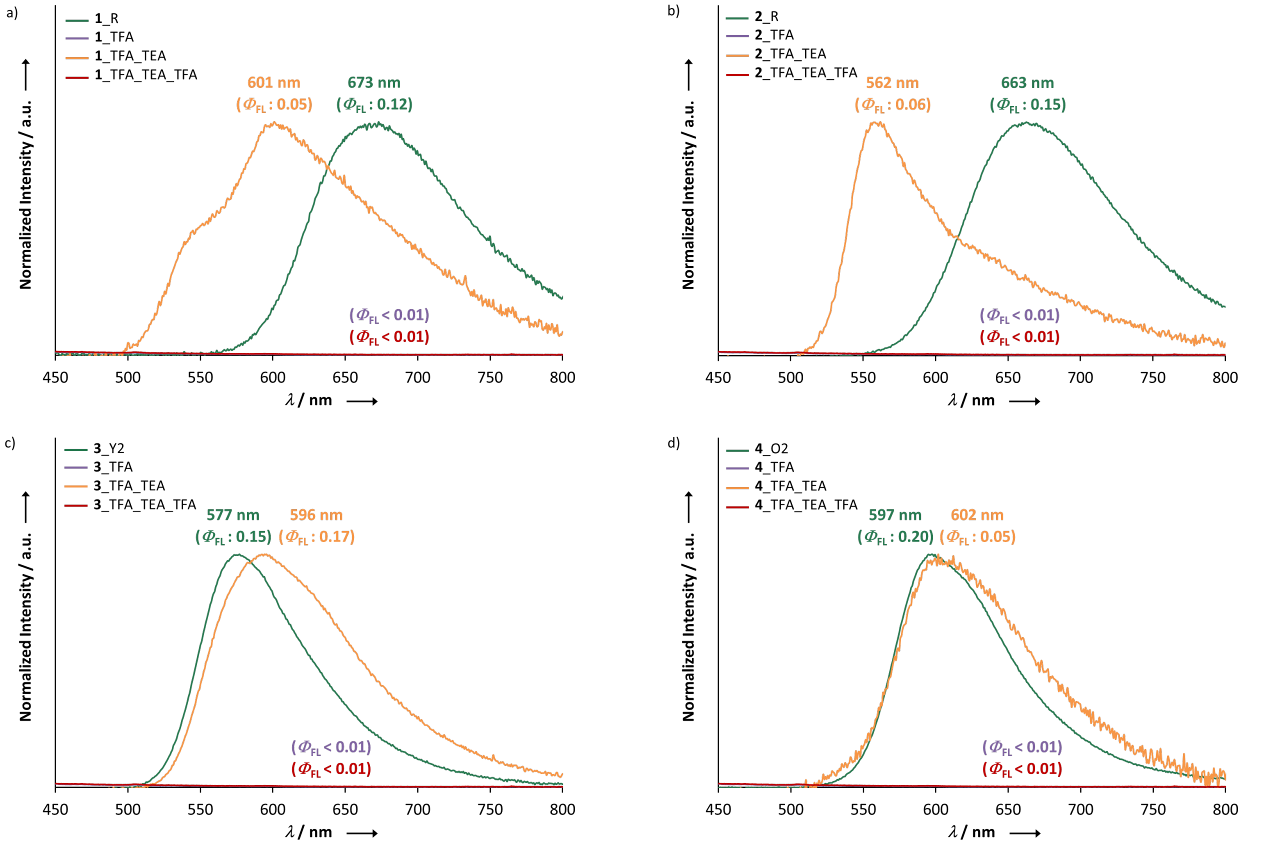

Figure S4. Emission spectra of acid/base-fumed solid samples of **1**–**4**.

Powder X-Ray Diffraction (PXRD) Analysis

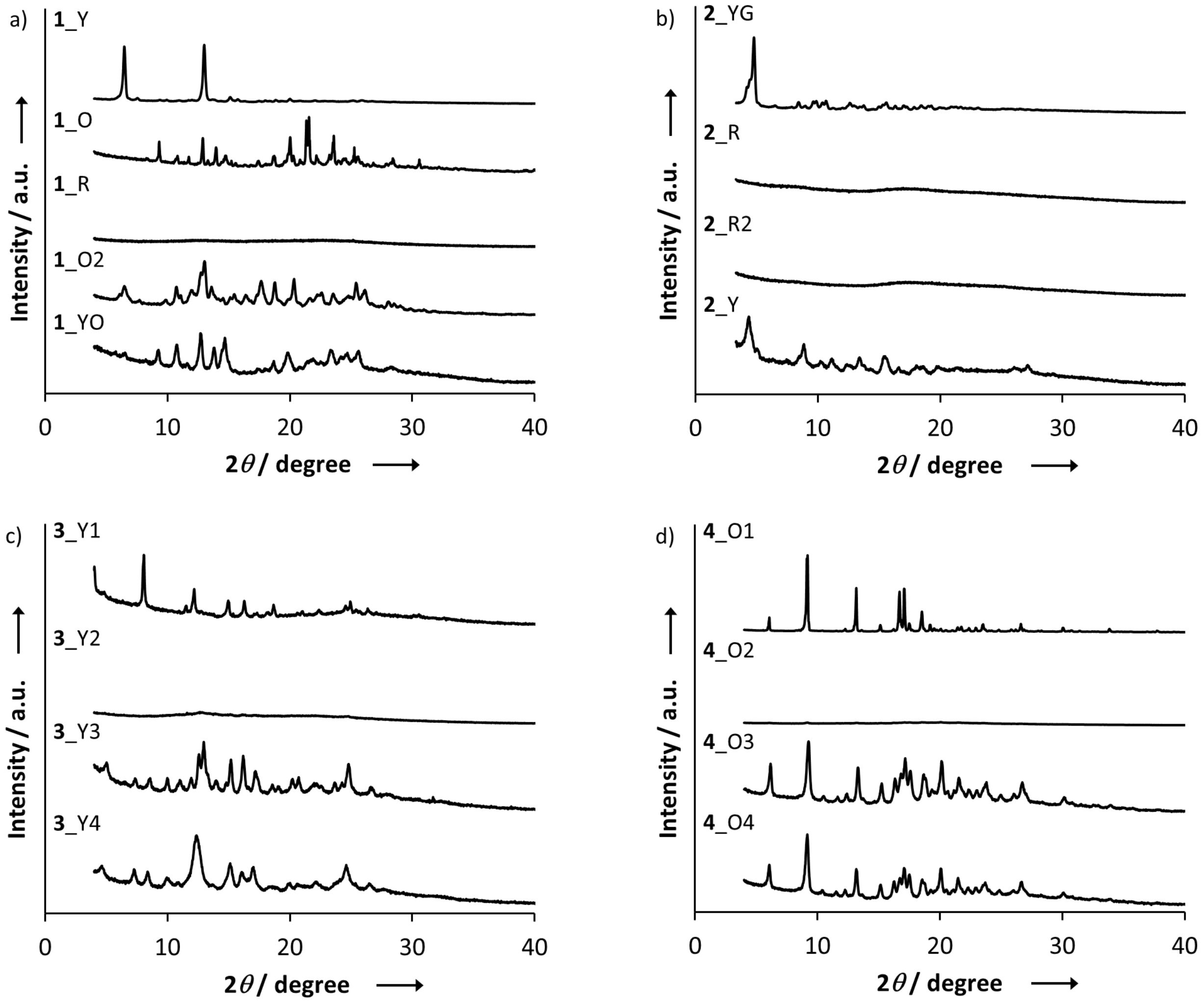

Figure S5. PXRD patterns of the solid samples **1**–**4**.

Differential Scanning Calorimetry (DSC) Analysis

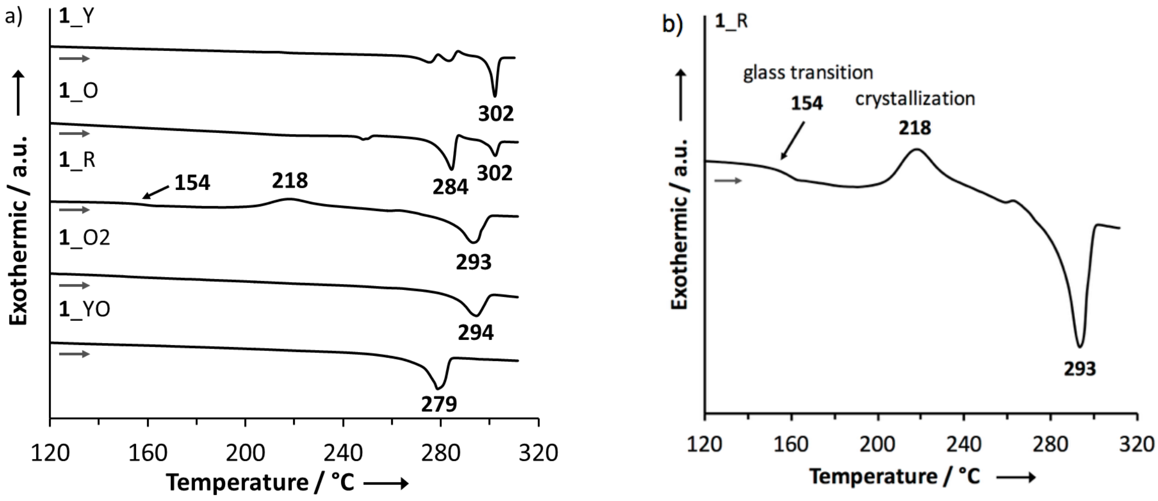

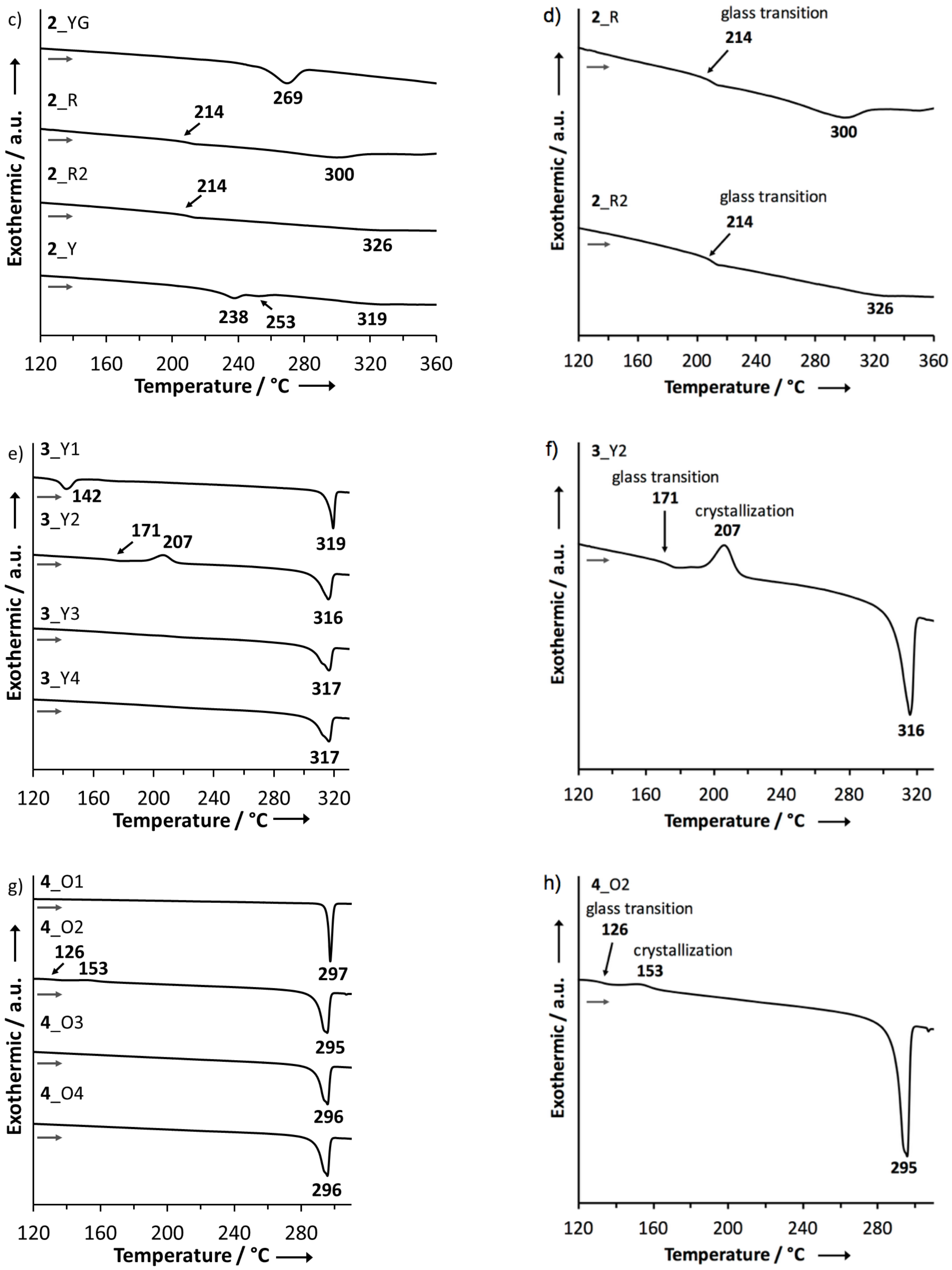

Figure S6. DSC curves of the solid samples **1**–**4**.

Single Crystal X-ray Crystallographic Data

The X-ray diffraction data of the single crystal of **1**_O were collected on a Rigaku R-AXIS RAPID diffractometer with graphite monochromated CuKα radiation (λ = 1.54187 Å) to a $2\theta_{max}$ value of 136.5° at 123 K. The crystal structure was solved by direct methods (SIR92)[S7] and expanded using Fourier techniques. The non-hydrogen atoms were refined anisotropically, and hydrogen atoms were refined using the riding model. The crystal data are summarized in Table S3. CCDC-1452024 contains the supplementary crystallographic data for **1**_O, which are available free of charge from the Cambridge Crystallographic Data Center (CCDC) via www.ccdc.cam.ac.uk/data_request/cif.

Table S3. Summary of crystallographic data of **1**_O.

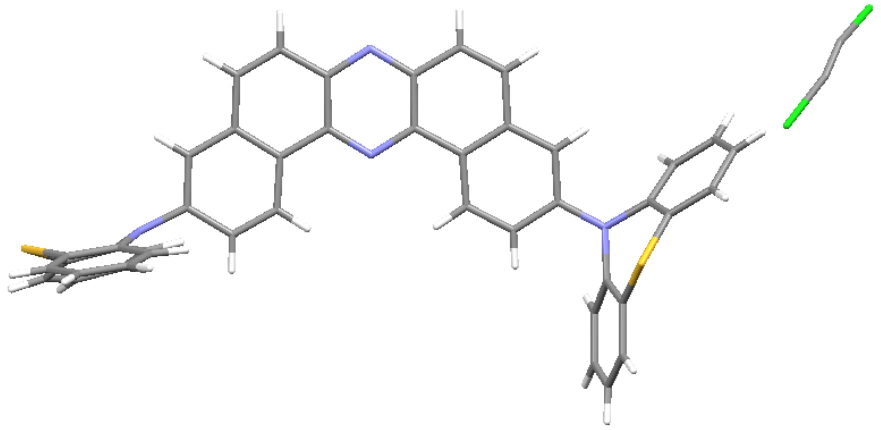

Empirical Formula	$C_{45}H_{26}ClN_4S_2$	
Formula Weight	722.30	
Crystal System	monoclinic	
Space Group	C2/c (#15)	
Unit cell dimensions	a = 30.0933(6) Å	α = 90 °
	b = 8.4390(2) Å	β = 95.5660(7) °
	c = 26.6268(5) Å	γ = 90 °
V	6730.2(3) Å^3	
Z	8	
Density (calculated)	1.426 g/cm^3	
Absorption coefficient	24.879 cm^{-1}	
R_1 [I>2σ(I)]	0.1215	
wR_2 (all data)	0.4004	
Crystal size	0.300 × 0.100 × 0.100 mm	
Goodness-of-fit on F^2	1.473	
Reflections collected/unique	43374/6159 [R(int) = 0.0648]	

a) b)

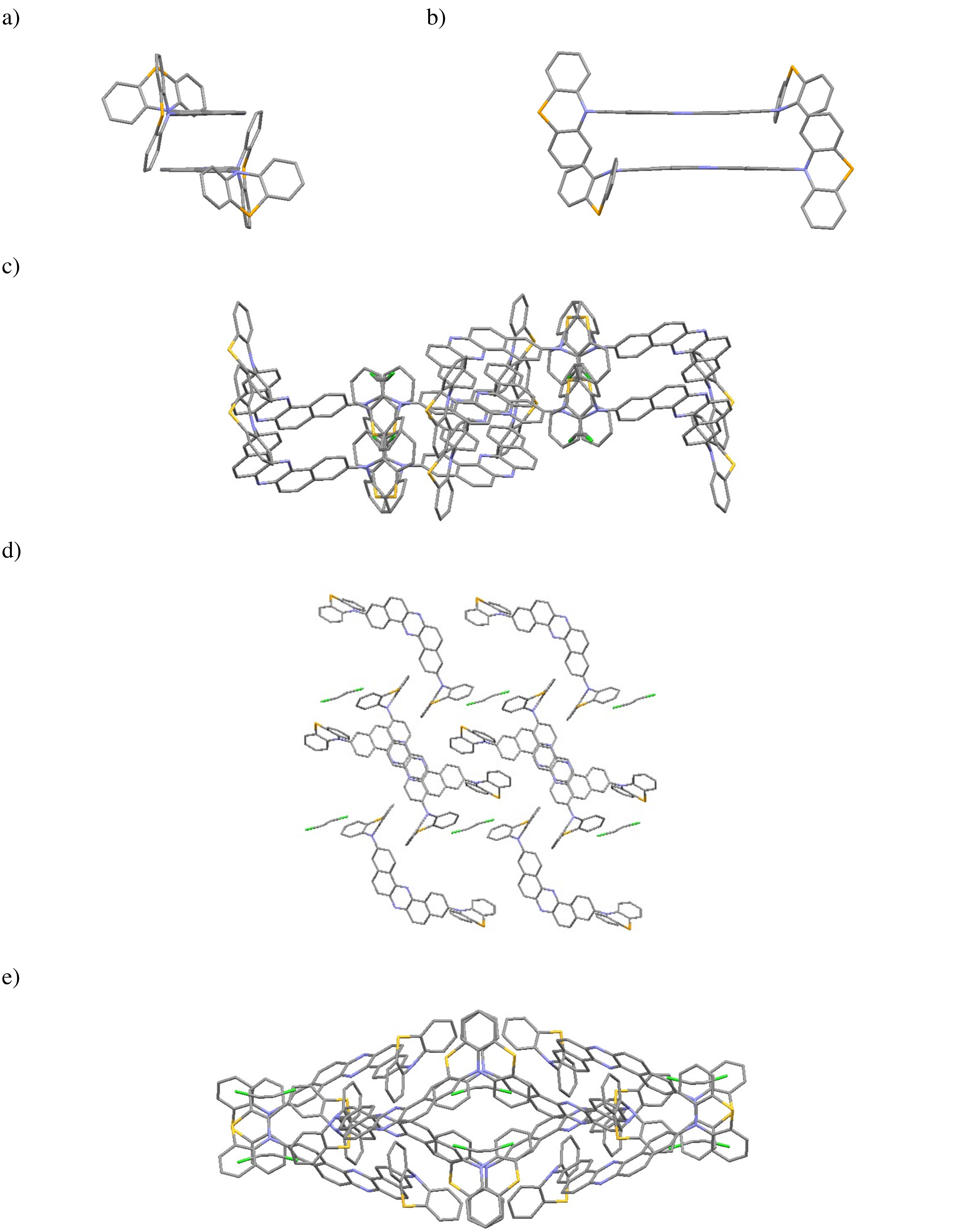

c)

d)

e)

Figure S7. Packing structures of **1**_O. a) Side and b) front view of a pair of two molecules; A view seen along the c) *a* axis, d) *b* axis and e) *c* axis.

UV-vis Absorption and Emission Spectra

Solvents were purged with N_2 for 30 min before use. Steady-stae UV-vis absorption and emission spectra were measured at room temperature using the solutions (1.0×10^{-5} M).

Table S4. Summary of UV-vis absorption and emission properties of solutions of **1**–**4**.

Compound	Solvent	Absorption		Emission		
		λ_{abs} (nm)	ε ($M^{-1}cm^{-1}$)	λ_{ex} (nm)	λ_{em} (nm)	Φ_{FL}
**1**	cyclohexane	296, 390, 413	69,900, 13,900, 22,000	300	543	0.06
**1**	toluene	298, 394, 416	58,800, 11,600, 19,300	300	657	0.07
**1**	THF	297, 394,-416	63,200, 12,800, 20,500	300	ND	<0.01
**1**	CH_2Cl_2	297, 395, 417	53,200, 9,000, 16,800	300	ND	<0.01
**1**	DMF	297, 396, 418	62,000, 12,200, 19,800	300	ND	<0.01
**2**	cyclohexane	297, 332, 391, 413, 451	65,100, 25,300, 12,500, 21,200, 12,400	300	575	0.11
**2**	toluene	298, 334, 394, 416, 451	60,600, 26,000, 12,400, 20,100, 13,300	300	682	0.04
**2**	THF	297, 335, 393, 416, 452	57,400, 27,800, 10,300, 17,500, 15,300	300	ND	<0.01
**3**	cyclohexane	296, 391, 413	94,100, 12,700, 18,600	300	484	0.16
**3**	toluene	297, 395, 416	91,600, 13,200, 19,000	300	552	0.11
**3**	THF	296, 394, 416	98,500, 14,400, 20,400	300	661	0.07
**3**	CH_2Cl_2	296, 395, 416	97,600, 14,000, 19,800	300	699	0.05
**3**	DMF	297, 396, 418	94,600, 14,500, 20,500	300	ND	<0.01
**4**	cyclohexane	275, 319, 337, 373, 444, 471	57,800, 48,900, 35,700, 26,000, 29,800, 55,500	300	492	0.56
**4**	toluene	321, 341, 374, 473	46,400, 35,000, 24,300, 42,900	300	512	0.44
**4**	THF	277, 319, 341, 474	53,800, 42,400, 33,100, 37,700	300	552	0.59
**4**	CH_2Cl_2	278, 321, 343, 476	54,000, 42,800, 33,800, 36,400	300	576	0.64
**4**	DMF	278, 319, 343, 477	55,200, 41,200, 34,100, 36,200	300	626	0.34

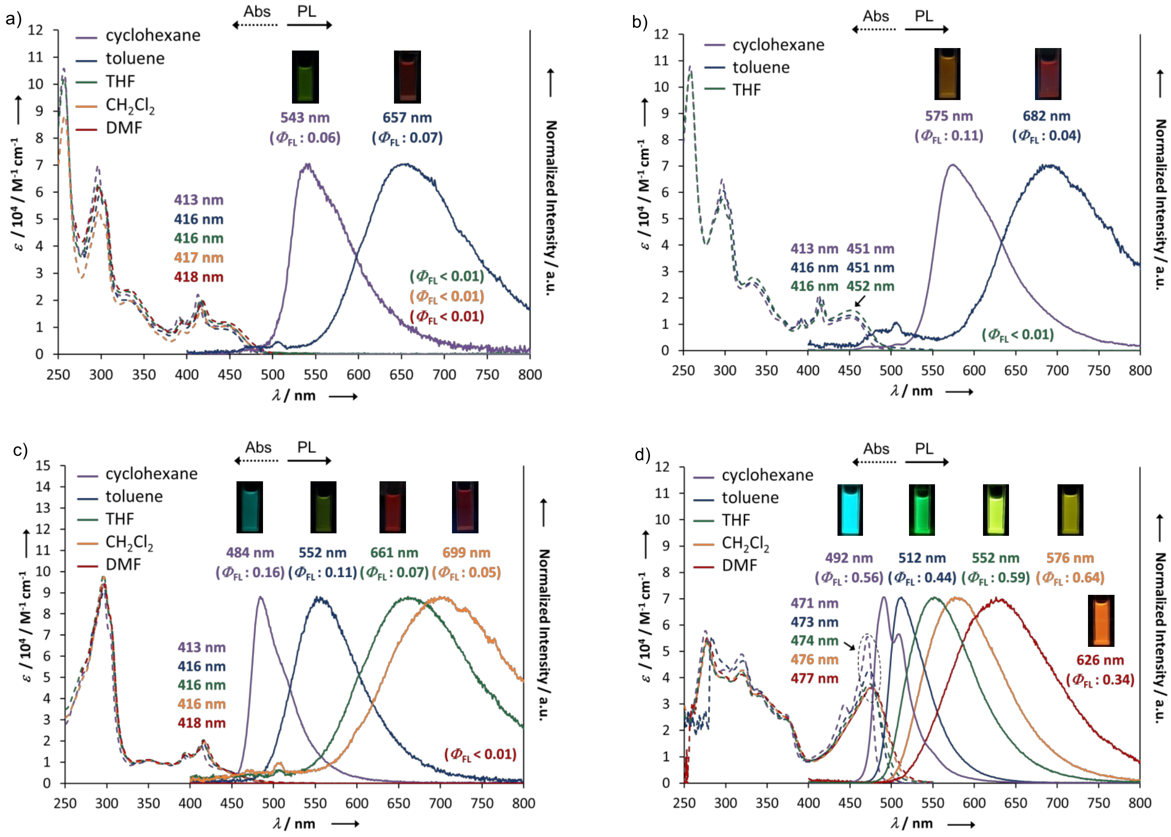

Figure S8. UV-vis absorption and emission spectra of solutions of **1**–**4**.

Concentration Effect on Emission Properties

Cyclohexane was purged with N_2 for 30 min before use. Emission spectra of **2** were measured at room temperature using the cyclohexane.

Table S5. The summary of the concentration effect on the emission properties of **2**

Concentration (M)	λ_{ex} (nm)	λ_{em} (nm)	Φ_{FL}
1 x 10^{-5}	300	575	0.11
1 x 10^{-4}	300	575	0.11
1 x 10^{-3}	300	577	0.10

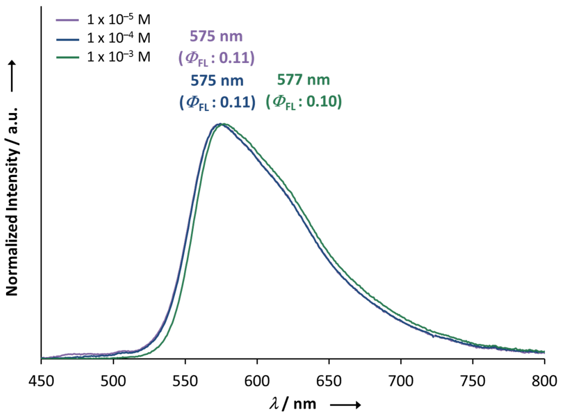

Figure S9. Emission spectra of cyclohexane solutions of **2** ranging from 1 × 10^{-5} to 1 × 10^{-3} M.

Diffuse Reflection Spectra

Table S6. Summary of diffuse reflection spectra of solids **1**.

Sample	λ_{onset} (nm)
**1**_Y	516
**1**_O	533
**1**_R	587
**1**_O2	573
**1**_YO	526

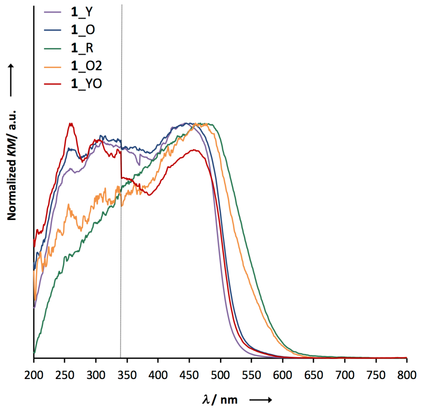

Figure S10. Diffuse reflection spectra of solids **1**. The dotted line indicates switching of the lamp.

Theoretical Calculations

All the molecules were geometrically optimized by the DFT method at the B3LYP/6-31+G(d,p) level of theory using Gaussian 09 package.[S8] The relative molecular energies of conformers **1** (Fig. 2) were calculated from the sum of electronic and thermal free energies, which were obtained from frequency analysis of the optimized structures of the conformers at the B3LYP/6-31+G(d,p) level. The excitation energies of the geometrically optimized molecules were calculated with the TD-DFT method at the same level of theory. Cartesian coordinates of the initial and optimized structures of **1** are listed below (Tables S7–14).

Table S7. Cartesian coordinates of the initial structure of axial-axial conformer (*syn*) of **1**.

atom	*x*	*y*	*z*
S	-1.94383	1.584678	-7.53526
S	-1.94383	1.584678	7.535259
N	-0.1227	-4.22501	0
N	0.212969	-1.42433	0
N	0.51404	0.577215	-6.18312
N	0.51404	0.577215	6.183122
C	-0.04003	-3.53497	-1.14688
C	-0.12537	-4.24203	-2.39632
C	-0.04128	-3.57549	-3.57989
C	0.136751	-2.14347	-3.63083
C	0.228934	-1.47497	-4.86787
C	0.402873	-0.09012	-4.93905
C	0.455619	0.637426	-3.72001
C	0.367217	-0.00922	-2.50195
C	0.216229	-1.40759	-2.41743
C	0.130924	-2.10807	-1.14968
C	0.130924	-2.10807	1.149681
C	0.216229	-1.40759	2.41743
C	0.367217	-0.00922	2.501955
C	0.455619	0.637426	3.720007
C	0.402873	-0.09012	4.93905
C	0.228934	-1.47497	4.86787
C	0.136751	-2.14347	3.630835
C	-0.04128	-3.57549	3.579887
C	-0.12537	-4.24203	2.396319
C	-0.04003	-3.53497	1.146883
C	0.230813	-0.10078	-7.41052

C	1.090529	-1.08326	-7.91612
C	0.778046	-1.74805	-9.10253
C	-0.37861	-1.40853	-9.81242
C	-1.21077	-0.38966	-9.34713
C	-0.90994	0.261783	-8.14519
C	-0.64448	2.616342	-6.87297
C	-0.71458	4.010944	-6.97032
C	0.319223	4.796103	-6.45833
C	1.444016	4.189658	-5.88928
C	1.525383	2.798653	-5.81371
C	0.466793	2.004107	-6.27011
C	0.466793	2.004107	6.270107
C	1.525383	2.798653	5.813715
C	1.444016	4.189658	5.889283
C	0.319223	4.796103	6.458333
C	-0.71458	4.010944	6.970321
C	-0.64448	2.616342	6.872969
C	-0.90994	0.261783	8.145194
C	-1.21077	-0.38966	9.347135
C	-0.37861	-1.40853	9.812416
C	0.778046	-1.74805	9.102527
C	1.090529	-1.08326	7.916118
C	0.230813	-0.10078	7.410521
H	-0.25825	-5.31842	-2.35198
H	-0.10761	-4.1189	-4.51891
H	0.148807	-2.06563	-5.77179
H	0.550637	1.71517	-3.73503
H	0.407039	0.564279	-1.58265
H	0.407039	0.564279	1.582646
H	0.550637	1.71517	3.735033
H	0.148807	-2.06563	5.771794
H	-0.10761	-4.1189	4.518909
H	-0.25825	-5.31842	2.351978
H	1.99214	-1.32761	-7.36342
H	1.442454	-2.52021	-9.47817
H	-0.61957	-1.91777	-10.7406
H	-2.08687	-0.09133	-9.91493
H	-1.57105	4.473344	-7.45151

H	0.257513	5.877925	-6.52721
H	2.262575	4.797772	-5.51659
H	2.399547	2.315833	-5.38845
H	2.399547	2.315833	5.388447
H	2.262575	4.797772	5.516592
H	0.257513	5.877925	6.527207
H	-1.57105	4.473344	7.451513
H	-2.08687	-0.09133	9.914932
H	-0.61957	-1.91777	10.7406
H	1.442454	-2.52021	9.478167
H	1.99214	-1.32761	7.363424

Table S8. Cartesian coordinates of the optimized structure of axial-axial conformer (*syn*) of **1**.

atom	*x*	*y*	*z*
S	-1.58163	1.943157	7.538075
S	-1.58163	1.943157	-7.53808
N	4.223625	0.126701	0
N	1.42338	-0.21275	0
N	-0.57769	-0.51476	6.183422
N	-0.57769	-0.51476	-6.18342
C	3.533652	0.043191	1.146888
C	4.240666	0.129339	2.396299
C	3.57428	0.044618	3.579896
C	2.142484	-0.13528	3.630935
C	1.47416	-0.22788	4.867974
C	0.089429	-0.40318	4.939235
C	-0.63809	-0.4571	3.720222
C	0.008449	-0.36817	2.502132
C	1.406655	-0.21579	2.417517
C	2.107027	-0.12976	1.14973
C	2.107027	-0.12976	-1.14973
C	1.406655	-0.21579	-2.41752
C	0.008449	-0.36817	-2.50213
C	-0.63809	-0.4571	-3.72022
C	0.089429	-0.40318	-4.93924
C	1.47416	-0.22788	-4.86797
C	2.142484	-0.13528	-3.63094
C	3.57428	0.044618	-3.5799

C	4.240666	0.129339	-2.3963
C	3.533652	0.043191	-1.14689
C	0.101092	-0.23346	7.410837
C	1.082838	-1.09475	7.915254
C	1.74832	-0.78422	9.101797
C	1.41028	0.372068	9.812993
C	0.392322	1.205943	9.348768
C	-0.25996	0.906866	8.146848
C	-2.61518	0.645702	6.874872
C	-4.00967	0.717706	6.972522
C	-4.79635	-0.3148	6.460255
C	-4.19153	-1.44005	5.890405
C	-2.80064	-1.52316	5.814318
C	-2.0045	-0.46584	6.27095
C	-2.0045	-0.46584	-6.27095
C	-2.80064	-1.52316	-5.81432
C	-4.19153	-1.44005	-5.89041
C	-4.79635	-0.3148	-6.46026
C	-4.00967	0.717706	-6.97252
C	-2.61518	0.645702	-6.87487
C	-0.25996	0.906866	-8.14685
C	0.392322	1.205943	-9.34877
C	1.41028	0.372068	-9.81299
C	1.74832	-0.78422	-9.1018
C	1.082838	-1.09475	-7.91525
C	0.101092	-0.23346	-7.41084
H	5.316891	0.26352	2.351932
H	4.117594	0.111661	4.518924
H	2.064651	-0.14632	5.77188
H	-1.71576	-0.55279	3.735208
H	-0.56506	-0.40887	1.582869
H	-0.56506	-0.40887	-1.58287
H	-1.71576	-0.55279	-3.73521
H	2.064651	-0.14632	-5.77188
H	4.117594	0.111661	-4.51892
H	5.316891	0.26352	-2.35193
H	1.326221	-1.996	7.361522
H	2.519802	-1.44994	9.476494

H	1.920077	0.611519	10.74126
H	0.095277	2.081922	9.917428
H	-4.47076	1.574534	7.45433
H	-5.87807	-0.2517	6.529433
H	-4.80073	-2.25771	5.517514
H	-2.31926	-2.39787	5.388522
H	-2.31926	-2.39787	-5.38852
H	-4.80073	-2.25771	-5.51751
H	-5.87807	-0.2517	-6.52943
H	-4.47076	1.574534	-7.45433
H	0.095277	2.081922	-9.91743
H	1.920077	0.611519	-10.7413
H	2.519802	-1.44994	-9.47649
H	1.326221	-1.996	-7.36152

Table S9. Cartesian coordinates of the initial structure of axial-axial conformer (*anti*) of **1**.

atom	*x*	*y*	*z*
S	-1.72669	7.778551	-1.34219
S	1.726692	-7.77855	-1.34219
N	0	0	4.214025
N	0	0	1.393306
N	0.662083	6.144943	-0.63405
N	-0.66208	-6.14494	-0.63405
C	0.109898	1.141667	3.518918
C	0.229265	2.385941	4.230318
C	0.347672	3.563382	3.55794
C	0.359448	3.613081	2.114904
C	0.492252	4.842499	1.439075
C	0.508747	4.91121	0.0432
C	0.35511	3.700494	-0.68412
C	0.225081	2.490091	-0.03048
C	0.23363	2.405617	1.375836
C	0.110634	1.144269	2.081972
C	-0.11063	-1.14427	2.081972
C	-0.23363	-2.40562	1.375836
C	-0.22508	-2.49009	-0.03048
C	-0.35511	-3.70049	-0.68412
C	-0.50875	-4.91121	0.0432

C	-0.49225	-4.8425	1.439075
C	-0.35945	-3.61308	2.114904
C	-0.34767	-3.56338	3.55794
C	-0.22927	-2.38594	4.230318
C	-0.1099	-1.14167	3.518918
C	0.456721	6.253753	-2.04524
C	1.355013	5.691441	-2.96048
C	1.117347	5.79218	-4.33185
C	0	6.491315	-4.80024
C	-0.86796	7.107199	-3.89762
C	-0.64349	6.985836	-2.52138
C	-0.47904	8.252838	-0.15509
C	-0.55952	9.474693	0.523234
C	0.436247	9.83069	1.433682
C	1.535046	8.988649	1.635336
C	1.627366	7.781013	0.942349
C	0.604357	7.387905	0.07098
C	-0.45672	-6.25375	-2.04524
C	-1.35501	-5.69144	-2.96048
C	-1.11735	-5.79218	-4.33185
C	0	-6.49132	-4.80024
C	0.867959	-7.1072	-3.89762
C	0.643485	-6.98584	-2.52138
C	0.47904	-8.25284	-0.15509
C	0.559524	-9.47469	0.523234
C	-0.43625	-9.83069	1.433682
C	-1.53505	-8.98865	1.635336
C	-1.62737	-7.78101	0.942349
C	-0.60436	-7.38791	0.07098
H	0.221415	2.342971	5.314911
H	0.437903	4.49822	4.105129
H	0.570309	5.743604	2.034323
H	0.324243	3.71676	-1.76554
H	0.107327	1.577425	-0.60385
H	-0.10733	-1.57743	-0.60385
H	-0.32424	-3.71676	-1.76554
H	-0.57031	-5.7436	2.034323
H	-0.4379	-4.49822	4.105129

H	-0.22142	-2.34297	5.314911
H	2.227193	5.165142	-2.58575
H	1.810298	5.337108	-5.03289
H	-0.18211	6.579496	-5.86698
H	-1.71344	7.687405	-4.25468
H	-1.39346	10.14251	0.329597
H	0.366614	10.77511	1.964815
H	2.325525	9.277271	2.321351
H	2.481835	7.125949	1.079763
H	-2.22719	-5.16514	-2.58575
H	-1.8103	-5.33711	-5.03289
H	0.182107	-6.5795	-5.86698
H	1.713439	-7.68741	-4.25468
H	1.393456	-10.1425	0.329597
H	-0.36661	-10.7751	1.964815
H	-2.32553	-9.27727	2.321351
H	-2.48184	-7.12595	1.079763

Table S10. Cartesian coordinates of the optimized structure of axial-axial conformer (*anti*) of **1**.

atom	*x*	*y*	*z*
S	-1.72669	7.778551	-1.34219
S	1.726692	-7.77855	-1.34219
N	0	0	4.214025
N	0	0	1.393306
N	0.662083	6.144943	-0.63405
N	-0.66208	-6.14494	-0.63405
C	0.109898	1.141667	3.518918
C	0.229265	2.385941	4.230318
C	0.347672	3.563382	3.55794
C	0.359448	3.613081	2.114904
C	0.492252	4.842499	1.439075
C	0.508747	4.91121	0.0432
C	0.35511	3.700494	-0.68412
C	0.225081	2.490091	-0.03048
C	0.23363	2.405617	1.375836
C	0.110634	1.144269	2.081972
C	-0.11063	-1.14427	2.081972
C	-0.23363	-2.40562	1.375836

C	-0.22508	-2.49009	-0.03048
C	-0.35511	-3.70049	-0.68412
C	-0.50875	-4.91121	0.0432
C	-0.49225	-4.8425	1.439075
C	-0.35945	-3.61308	2.114904
C	-0.34767	-3.56338	3.55794
C	-0.22927	-2.38594	4.230318
C	-0.1099	-1.14167	3.518918
C	0.456721	6.253753	-2.04524
C	1.355013	5.691441	-2.96048
C	1.117347	5.79218	-4.33185
C	0	6.491315	-4.80024
C	-0.86796	7.107199	-3.89762
C	-0.64349	6.985836	-2.52138
C	-0.47904	8.252838	-0.15509
C	-0.55952	9.474693	0.523234
C	0.436247	9.83069	1.433682
C	1.535046	8.988649	1.635336
C	1.627366	7.781013	0.942349
C	0.604357	7.387905	0.07098
C	-0.45672	-6.25375	-2.04524
C	-1.35501	-5.69144	-2.96048
C	-1.11735	-5.79218	-4.33185
C	0	-6.49132	-4.80024
C	0.867959	-7.1072	-3.89762
C	0.643485	-6.98584	-2.52138
C	0.47904	-8.25284	-0.15509
C	0.559524	-9.47469	0.523234
C	-0.43625	-9.83069	1.433682
C	-1.53505	-8.98865	1.635336
C	-1.62737	-7.78101	0.942349
C	-0.60436	-7.38791	0.07098
H	0.221415	2.342971	5.314911
H	0.437903	4.49822	4.105129
H	0.570309	5.743604	2.034323
H	0.324243	3.71676	-1.76554
H	0.107327	1.577425	-0.60385
H	-0.10733	-1.57743	-0.60385

H	-0.32424	-3.71676	-1.76554
H	-0.57031	-5.7436	2.034323
H	-0.4379	-4.49822	4.105129
H	-0.22142	-2.34297	5.314911
H	2.227193	5.165142	-2.58575
H	1.810298	5.337108	-5.03289
H	-0.18211	6.579496	-5.86698
H	-1.71344	7.687405	-4.25468
H	-1.39346	10.14251	0.329597
H	0.366614	10.77511	1.964815
H	2.325525	9.277271	2.321351
H	2.481835	7.125949	1.079763
H	-2.22719	-5.16514	-2.58575
H	-1.8103	-5.33711	-5.03289
H	0.182107	-6.5795	-5.86698
H	1.713439	-7.68741	-4.25468
H	1.393456	-10.1425	0.329597
H	-0.36661	-10.7751	1.964815
H	-2.32553	-9.27727	2.321351
H	-2.48184	-7.12595	1.079763

Table S11. Cartesian coordinates of the initial structure of equatorial-axial conformer of **1**.

atom	*x*	*y*	*z*
S	-7.654	-1.77057	-1.78935
S	9.202876	-0.83293	-0.09511
N	-0.10524	4.253314	-0.26271
N	-0.02688	1.44769	0.029132
N	-6.14016	-0.70099	0.536777
N	6.202286	-0.39874	-0.0259
C	-1.22905	3.534164	-0.14659
C	-2.49734	4.211304	-0.17387
C	-3.65846	3.51485	-0.05452
C	-3.67084	2.08022	0.105815
C	-4.88615	1.384342	0.235065
C	-4.92062	-0.00456	0.395561
C	-3.68315	-0.70246	0.392407
C	-2.4857	-0.02798	0.266117
C	-2.43877	1.372596	0.129395

C	-1.19436	2.103908	0.004326
C	1.100397	2.161887	-0.089
C	2.386516	1.482995	-0.06846
C	2.486517	0.08324	0.067319
C	3.721823	-0.53982	0.082526
C	4.9043	0.22351	-0.03677
C	4.825014	1.599186	-0.17039
C	3.575048	2.255691	-0.19019
C	3.488134	3.686641	-0.33285
C	2.287732	4.325471	-0.35539
C	1.061163	3.586875	-0.23527
C	-7.39931	-0.04942	0.344283
C	-7.8598	0.932619	1.228839
C	-9.08269	1.563432	1.002079
C	-9.87375	1.189228	-0.08728
C	-9.4514	0.169549	-0.93948
C	-8.21457	-0.44681	-0.7274
C	-6.87803	-2.77135	-0.52833
C	-6.95944	-4.16649	-0.56732
C	-6.3556	-4.92515	0.434849
C	-5.7087	-4.29122	1.498827
C	-5.64915	-2.89902	1.552581
C	-6.19917	-2.13016	0.52039
C	6.684717	-0.96813	-1.23479
C	5.835116	-1.21541	-2.32504
C	6.329653	-1.76393	-3.50899
C	7.675319	-2.10602	-3.62559
C	8.529039	-1.87537	-2.54643
C	8.049531	-1.2889	-1.37424
C	8.146479	-1.04719	1.323497
C	8.712435	-1.41947	2.543724
C	7.940173	-1.44759	3.70537
C	6.588083	-1.12039	3.627022
C	6.006371	-0.78733	2.40314
C	6.773274	-0.74619	1.227645
H	-2.48435	5.29024	-0.29295
H	-4.61272	4.035449	-0.077
H	-5.80583	1.954667	0.194652

H	-3.67075	-1.78144	0.475292
H	-1.55318	-0.58186	0.263853
H	1.573571	-0.4945	0.158016
H	3.793757	-1.61863	0.185106
H	5.740039	2.177238	-0.26176
H	4.413117	4.249891	-0.42412
H	2.212719	5.403018	-0.4636
H	-7.2465	1.204078	2.082492
H	-9.42314	2.336336	1.684869
H	-10.831	1.672466	-0.25968
H	-10.0793	-0.15606	-1.76359
H	-7.49927	-4.65052	-1.37584
H	-6.41221	-6.0091	0.396942
H	-5.26279	-4.87908	2.29571
H	-5.1653	-2.39691	2.384696
H	4.782891	-0.96851	-2.25354
H	5.647098	-1.93157	-4.33746
H	8.060788	-2.5438	-4.54149
H	9.583273	-2.12917	-2.61469
H	9.770139	-1.66524	2.579794
H	8.393014	-1.71853	4.654383
H	5.968261	-1.13184	4.519254
H	4.950786	-0.54762	2.365357

Table S12. Cartesian coordinates of the optimized structure of equatorial-axial conformer of **1**.

atom	*x*	*y*	*z*
S	-7.55176	-1.74085	-1.80972
S	9.197853	-0.87637	-0.09145
N	-0.12697	4.251893	-0.28345
N	-0.03682	1.451584	0.032917
N	-6.14304	-0.71499	0.607164
N	6.203205	-0.37342	-0.0282
C	-1.24834	3.531665	-0.15496
C	-2.51937	4.205242	-0.18141
C	-3.67819	3.505116	-0.04822
C	-3.68416	2.071031	0.125965
C	-4.89769	1.371488	0.269859
C	-4.9253	-0.01613	0.442303

C	-3.68609	-0.71105	0.43976
C	-2.49044	-0.03307	0.299275
C	-2.44955	1.367251	0.148575
C	-1.20718	2.102161	0.008723
C	1.087825	2.167707	-0.09702
C	2.377613	1.493678	-0.076
C	2.48464	0.094271	0.071856
C	3.723699	-0.52337	0.088345
C	4.902432	0.244254	-0.04147
C	4.816932	1.618324	-0.18755
C	3.562801	2.2702	-0.20924
C	3.469843	3.700425	-0.36514
C	2.265968	4.33543	-0.38878
C	1.042822	3.59217	-0.256
C	-7.39913	-0.06928	0.374156
C	-7.90103	0.890966	1.26033
C	-9.11552	1.524988	0.994708
C	-9.85448	1.17596	-0.14071
C	-9.39027	0.17782	-0.99826
C	-8.16158	-0.44294	-0.7444
C	-6.81745	-2.76386	-0.54262
C	-6.87923	-4.16005	-0.62081
C	-6.312	-4.93812	0.389277
C	-5.72204	-4.32483	1.499488
C	-5.68077	-2.93284	1.589402
C	-6.19385	-2.14432	0.552835
C	6.676889	-0.95972	-1.23293
C	5.825595	-1.18604	-2.32802
C	6.310213	-1.75178	-3.5095
C	7.64776	-2.13044	-3.61898
C	8.503049	-1.91737	-2.53587
C	8.034896	-1.31407	-1.36627
C	8.135272	-1.05306	1.325939
C	8.693219	-1.42479	2.551317
C	7.923366	-1.41644	3.716372
C	6.580108	-1.05094	3.636777
C	6.00477	-0.7184	2.408246
C	6.769063	-0.71832	1.228824

H	-2.51056	5.282778	-0.31055
H	-4.63355	4.023029	-0.07018
H	-5.81955	1.93811	0.232224
H	-3.66929	-1.78883	0.534124
H	-1.5564	-0.5836	0.297383
H	1.574903	-0.48665	0.171133
H	3.800178	-1.60067	0.200259
H	5.729975	2.197954	-0.28705
H	4.39141	4.26716	-0.46561
H	2.186002	5.411339	-0.50663
H	-7.32483	1.142503	2.145161
H	-9.48976	2.280133	1.679092
H	-10.8042	1.660897	-0.34484
H	-9.97975	-0.1287	-1.857
H	-7.3765	-4.62948	-1.46414
H	-6.3541	-6.02091	0.321364
H	-5.30611	-4.9286	2.300174
H	-5.23951	-2.44488	2.45277
H	4.780998	-0.90867	-2.2647
H	5.627541	-1.90187	-4.34057
H	8.025261	-2.58199	-4.53093
H	9.550127	-2.19898	-2.59883
H	9.743361	-1.69946	2.588402
H	8.370676	-1.68807	4.667245
H	5.963281	-1.0299	4.530301
H	4.957597	-0.44614	2.372039

Table S13. Cartesian coordinates of the initial structure of equatorial-equatorial conformer of **1**.

atom	*x*	*y*	*z*
N	0.06101	4.307628	0
N	0.017664	1.490045	0
C	0.062068	4.327549	-2.3953
C	0.05258	3.65402	-3.57761
C	0.030659	2.212679	-3.62621
C	0.021447	1.525146	-4.86065
C	0.000237	0.141051	-4.90403
C	-0.01224	-0.59888	-3.70111
C	-0.00338	0.054197	-2.48009

C	0.01807	1.463438	-2.41688
C	0.028289	2.177248	-1.14852
C	0.050408	3.613245	-1.14732
C	0.062068	4.327549	2.395303
C	0.05258	3.65402	3.577607
C	0.030659	2.212679	3.626206
C	0.021447	1.525146	4.860647
C	0.000237	0.141051	4.904026
C	-0.01224	-0.59888	3.701114
C	-0.00338	0.054197	2.480089
C	0.01807	1.463438	2.416878
C	0.028289	2.177248	1.148522
C	0.050408	3.613245	1.147325
H	0.078799	5.411603	-2.35056
H	0.061632	4.19853	-4.51783
H	0.031143	2.083783	-5.79193
H	-0.02847	-1.68358	-3.74469
H	-0.01262	-0.50714	-1.5529
H	0.078799	5.411603	2.350558
H	0.061632	4.19853	4.517826
H	0.031143	2.083783	5.791929
H	-0.02847	-1.68358	3.744694
H	-0.01262	-0.50714	1.5529
C	1.223791	-1.01512	-6.68884
C	-1.25199	-0.97598	-6.69431
C	2.362706	-1.13165	-5.87408
C	-2.39779	-1.05627	-5.88481
C	3.566868	-1.61763	-6.38875
H	2.316222	-0.83142	-4.83494
C	2.531643	-1.9179	-8.53872
C	-3.61448	-1.5035	-6.40511
H	-2.34659	-0.75766	-4.84543
C	-2.57953	-1.83645	-8.55038
C	3.657622	-2.02343	-7.71959
H	4.431006	-1.68437	-5.73452
H	2.579286	-2.22238	-9.58014
C	-3.71203	-1.90612	-7.73644
H	-4.48332	-1.54263	-5.75488

H	-2.63211	-2.13914	-9.59209
H	4.587934	-2.41378	-8.11974
H	-4.65247	-2.26657	-8.14094
N	-0.00758	-0.51332	-6.18561
C	-1.25199	-0.97598	6.694313
C	1.223791	-1.01512	6.68884
C	-2.39779	-1.05627	5.884809
C	2.362706	-1.13165	5.874083
C	-3.61448	-1.5035	6.405111
H	-2.34659	-0.75766	4.845431
C	-2.57953	-1.83645	8.550378
C	3.566868	-1.61763	6.388745
H	2.316222	-0.83142	4.834936
C	2.531643	-1.9179	8.538719
C	-3.71203	-1.90612	7.73644
H	-4.48332	-1.54263	5.754884
H	-2.63211	-2.13914	9.592086
C	3.657622	-2.02343	7.719592
H	4.431006	-1.68437	5.734516
H	2.579286	-2.22238	9.580142
H	-4.65247	-2.26657	8.140937
H	4.587934	-2.41378	8.119741
N	-0.00758	-0.51332	6.185606
C	1.335854	-1.39386	-8.04208
C	-1.36991	-1.35088	-8.04816
S	-0.00996	-1.08682	-9.16615
C	-1.36991	-1.35088	8.048162
C	1.335854	-1.39386	8.042083
S	-0.00996	-1.08682	9.166151

Table S14. Cartesian coordinates of the optimized structure of equatorial-equatorial conformer of **1**.

atom	*x*	*y*	*z*
N	-4.30808	0.002601	0
N	-1.49014	0.000735	0
C	-4.32797	0.002661	2.395281
C	-3.65443	0.002272	3.577618
C	-2.21291	0.001331	3.626233
C	-1.52531	0.000959	4.860656

C	-0.14107	0.000036	4.904038
C	0.599002	-0.00054	3.701152
C	-0.05413	-0.00017	2.48011
C	-1.46354	0.000766	2.416894
C	-2.17743	0.001196	1.148524
C	-3.61358	0.002148	1.14731
C	-4.32797	0.002661	-2.39528
C	-3.65443	0.002272	-3.57762
C	-2.21291	0.001331	-3.62623
C	-1.52531	0.000959	-4.86066
C	-0.14107	0.000036	-4.90404
C	0.599002	-0.00054	-3.70115
C	-0.05413	-0.00017	-2.48011
C	-1.46354	0.000766	-2.41689
C	-2.17743	0.001196	-1.14852
C	-3.61358	0.002148	-1.14731
H	-5.41215	0.003379	2.350557
H	-4.19899	0.002673	4.51785
H	-2.08403	0.001395	5.791947
H	1.683815	-0.00126	3.744801
H	0.507311	-0.00059	1.552941
H	-5.41215	0.003379	-2.35056
H	-4.19899	0.002673	-4.51785
H	-2.08403	0.001395	-5.79195
H	1.683815	-0.00126	-3.7448
H	0.507311	-0.00059	-1.55294
C	0.994729	-1.23866	6.691645
C	0.996418	1.237482	6.691458
C	1.092367	-2.38126	5.879536
C	1.095604	2.379835	5.879188
C	1.557896	-3.59226	6.39708
H	0.793079	-2.33216	4.840242
C	1.875235	-2.55724	8.544694
C	1.5628	3.590274	6.39655
H	0.796239	2.331	4.839906
C	1.878743	2.555129	8.544308
C	1.961838	-3.68676	7.728222
H	1.610103	-4.45894	5.744906

H	2.178775	-2.60755	9.586264
C	1.966886	3.684409	7.727673
H	1.616186	4.456792	5.744251
H	2.182358	2.605182	9.58587
H	2.336332	-4.62259	8.130644
H	2.342664	4.619784	8.129957
N	0.513385	-0.0003	6.1856
C	0.996418	1.237482	-6.69146
C	0.994729	-1.23866	-6.69165
C	1.095604	2.379835	-5.87919
C	1.092367	-2.38126	-5.87954
C	1.5628	3.590274	-6.39655
H	0.796239	2.331	-4.83991
C	1.878743	2.555129	-8.54431
C	1.557896	-3.59226	-6.39708
H	0.793079	-2.33216	-4.84024
C	1.875235	-2.55724	-8.54469
C	1.966886	3.684409	-7.72767
H	1.616186	4.456792	-5.74425
H	2.182358	2.605182	-9.58587
C	1.961838	-3.68676	-7.72822
H	1.610103	-4.45894	-5.74491
H	2.178775	-2.60755	-9.58626
H	2.342664	4.619784	-8.12996
H	2.336332	-4.62259	-8.13064
N	0.513385	-0.0003	-6.1856
C	1.371825	-1.35386	8.045097
C	1.373679	1.352371	8.044892
S	1.088027	-0.00047	9.166195
C	1.373679	1.352371	-8.04489
C	1.371825	-1.35386	-8.0451
S	1.088027	-0.00047	-9.1662

Table S15. Selected excitation energies and oscillator strength (*f*) of the conformers of **1**.[a]

	Axial-Axial (*syn*)	Axial-Axial (*anti*)	Equatorial-Axial	Equatorial-Equatorial
λ [nm] (*f*)	439.04 (0.5653)	439.84 (0.5688)	538.22 (0.0001)	596.35 (0)
transition mode	H→L (95%)	H→L (95%)	H→L (99%)	H→L (99%)
λ [nm] (*f*)	354.80 (0.1493)	355.13 (0.1599)	449.23 (0.3059)	389.79 (0.3807)
transition mode	H–2→L (48%)	H–2→L (46%)	H–1→L (97%)	H–4→L (90%)
λ [nm] (*f*)	339.81 (0.6522)	340.28 (0.6491)	348.29 (0.5908)	377.05 (0.0214)
transition mode	H–1→L+1 (37%)	H–1→L+1 (36%)	H–1→L+1 (66%)	H–5→L (94%)
λ [nm] (*f*)	338.43 (0.1207)	338.79 (0.1211)	305.77 (0.1559)	317.19 (0.0216)
transition mode	H–3→L (50%)	H–3→L (50%)	H–1→L+5 (22%)	H–6→L (50%)
λ [nm] (*f*)				315.23 (0.0255)
transition mode				H–11→L (57%)

[a] The coefficient percentages of orbitals involved in the transitions are shown in parentheses.

Time-resolved photoluminescence analysis

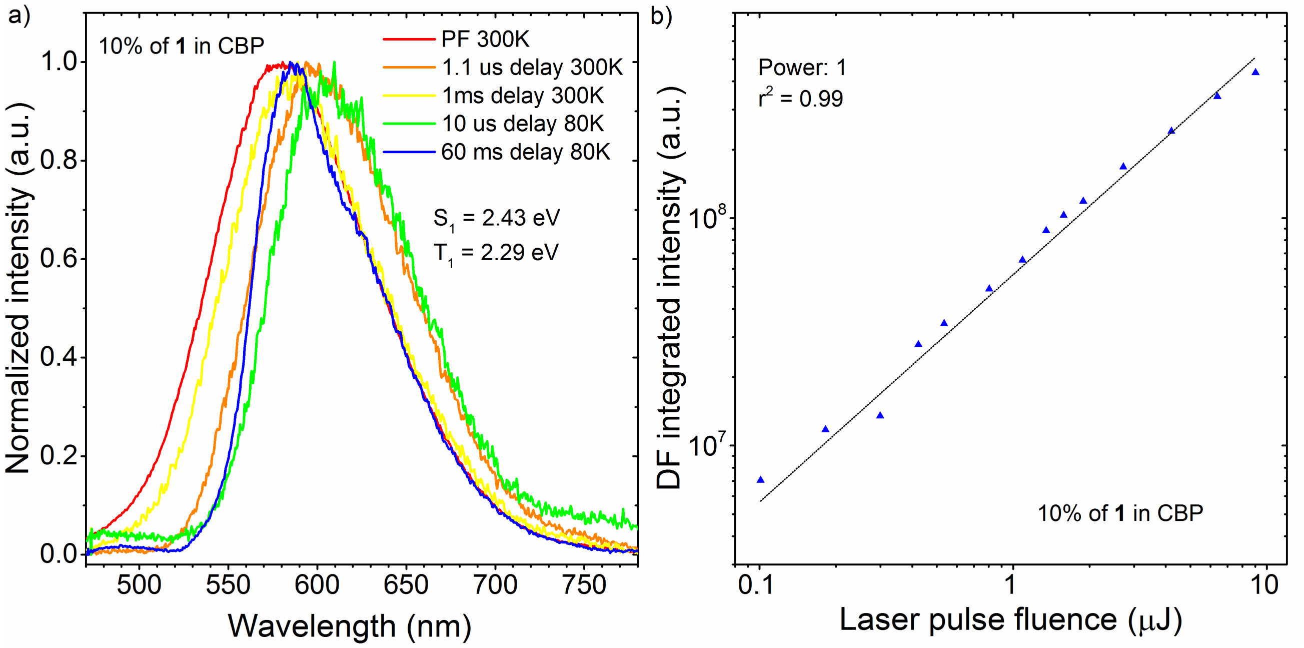

Figure S11. Emission spectra recorded at different delay time for 10% **1**: CBP blend. Power dependence of delayed fluorescence from 10% **1**: CBP blend. Recorded at 300K.

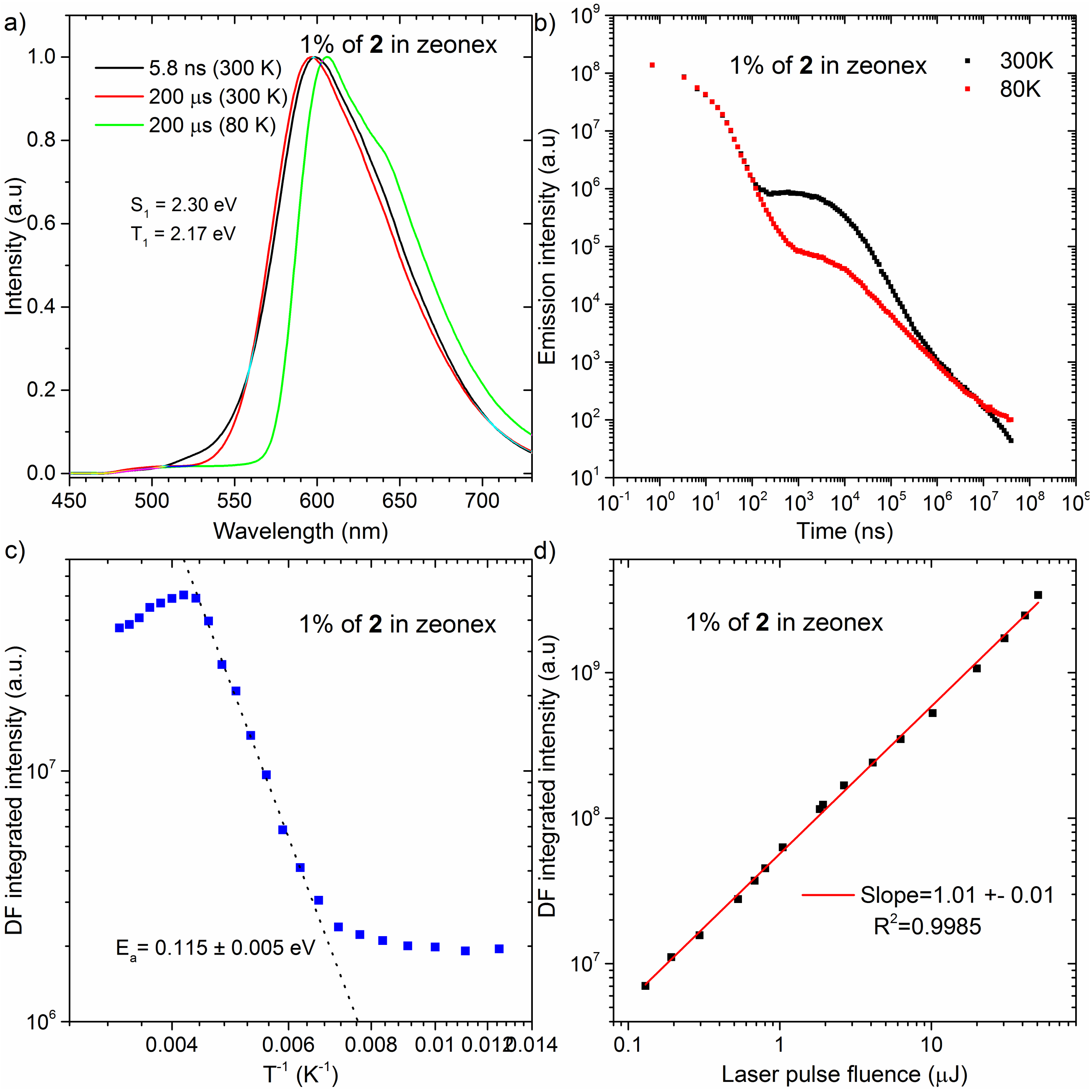

Figure S12. (a) PF, DF and PH spectra of 1 wt% **2**:zeonex® blended film. (b) Transient decays of 1 wt% **2**:zeonex® blended film at 300K and 80K. (c) Temperature dependence of delayed fluorescence of 1 wt% **2**:zeonex® blended film. (d) Power dependence of delayed fluorescence of 1 wt% **2**:zeonex® blended film at 300K.

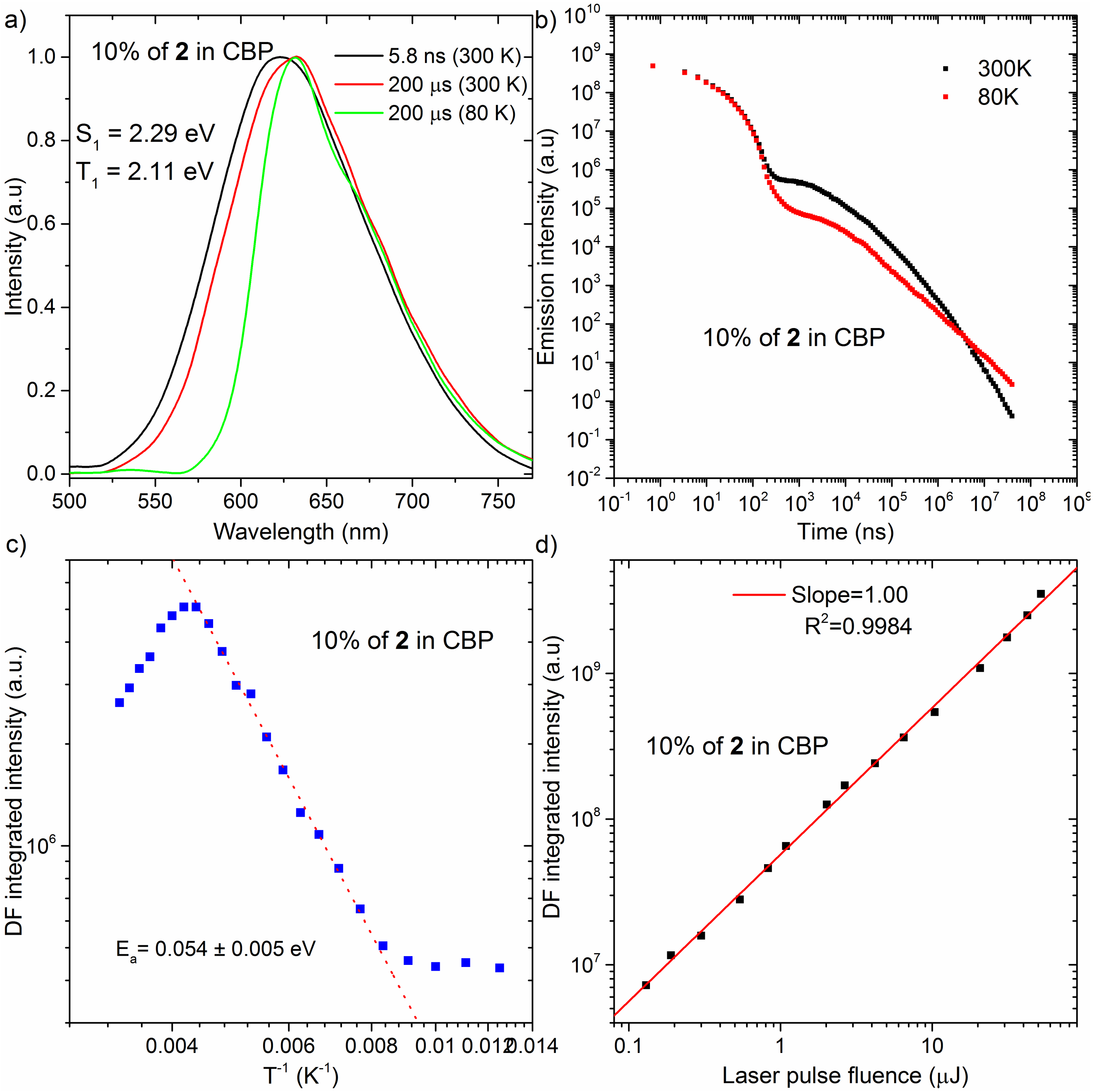

Figure S13. (a) PF, DF and PH spectra of 10 wt% **2**:CBP blended film. (b) Transient decays of 10 wt% **2**:CBP blended film at 300K and 80K. (c) Temperature dependence of delayed fluorescence of 10 wt% **2**:CBP blended film. (d) Power dependence of delayed fluorescence of 10 wt% **2**:CBP blended film at 300K.

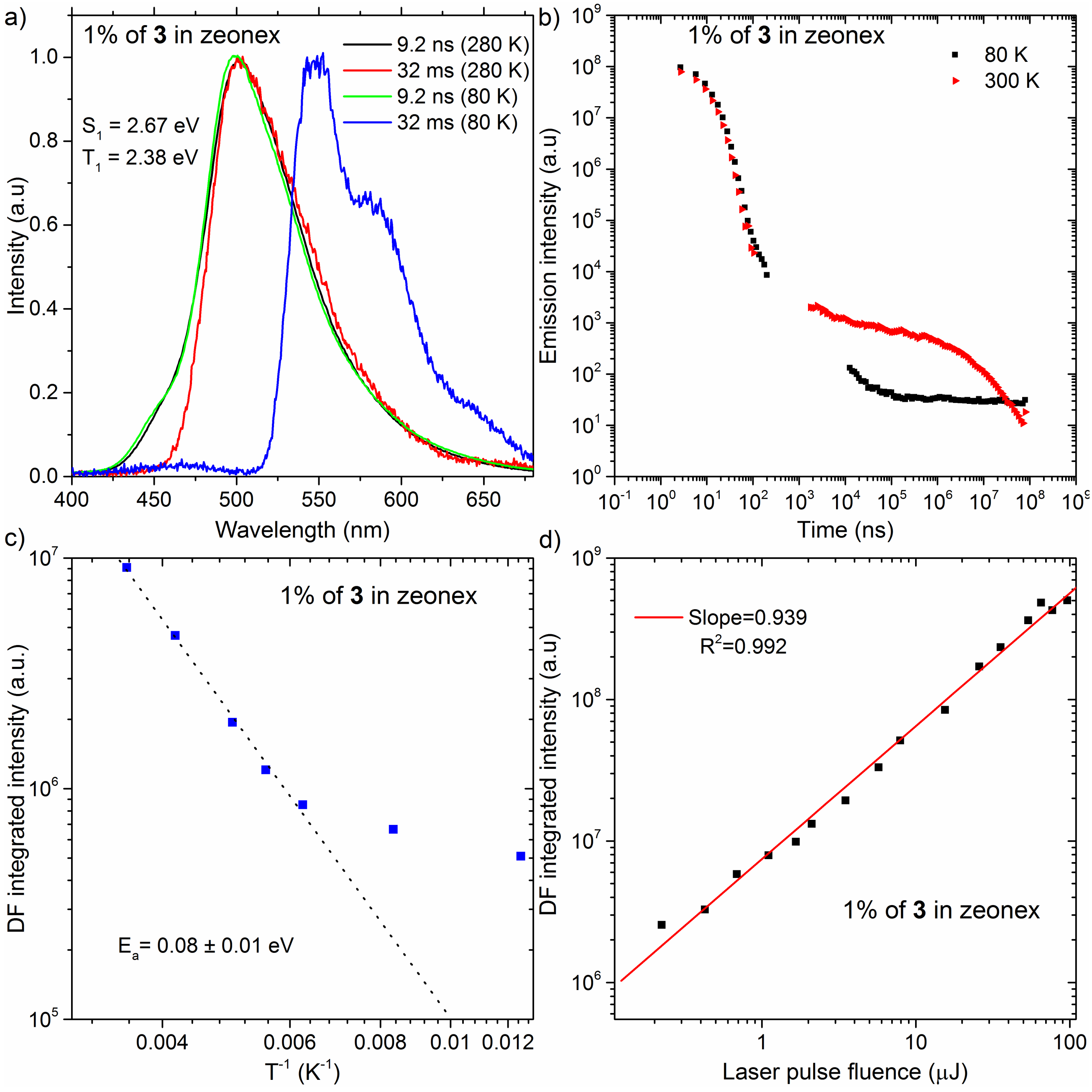

Figure S14. (a) PF, DF and PH spectra of 1 wt% **3**:zeonex® blended film. (b) Transient decays of 1 wt% **3**:zeonex® blended film at 300K and 80K. (c) Temperature dependence of delayed fluorescence of 1 wt% **3**:zeonex® blended film. (d) Power dependence of delayed fluorescence of 1 wt% **3**:zeonex® blended film at 300K.

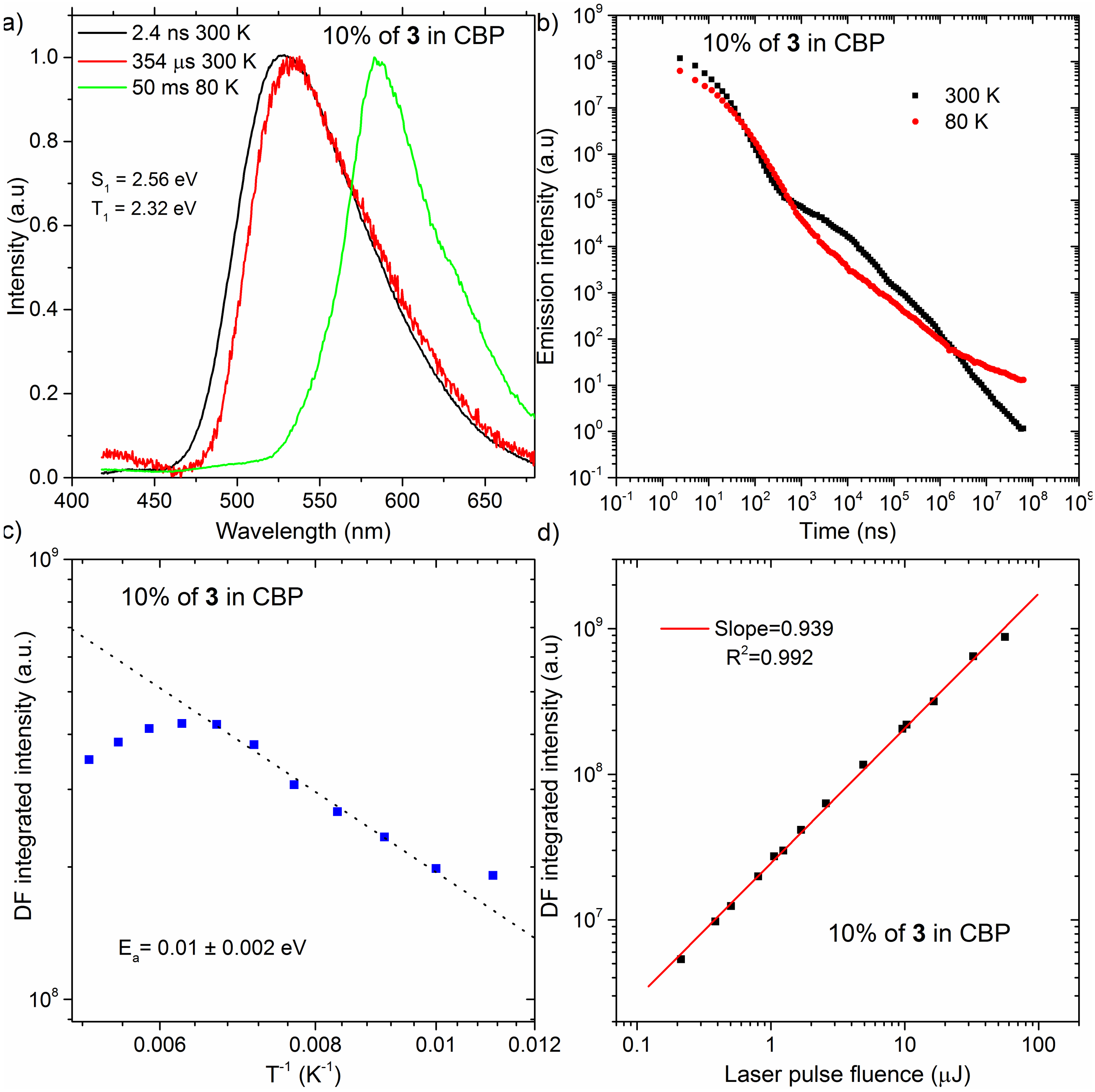

Figure S15. (a) PF, DF and PH spectra of 10 wt% **3**:CBP blended film. (b) Transient decays of 10 wt% **3**:CBP blended film at 300K and 80K. (c) Temperature dependence of delayed fluorescence of 10 wt% **3**:CBP blended film. (d) Power dependence of delayed fluorescence of 10 wt% **3**:CBP blended film at 300K.

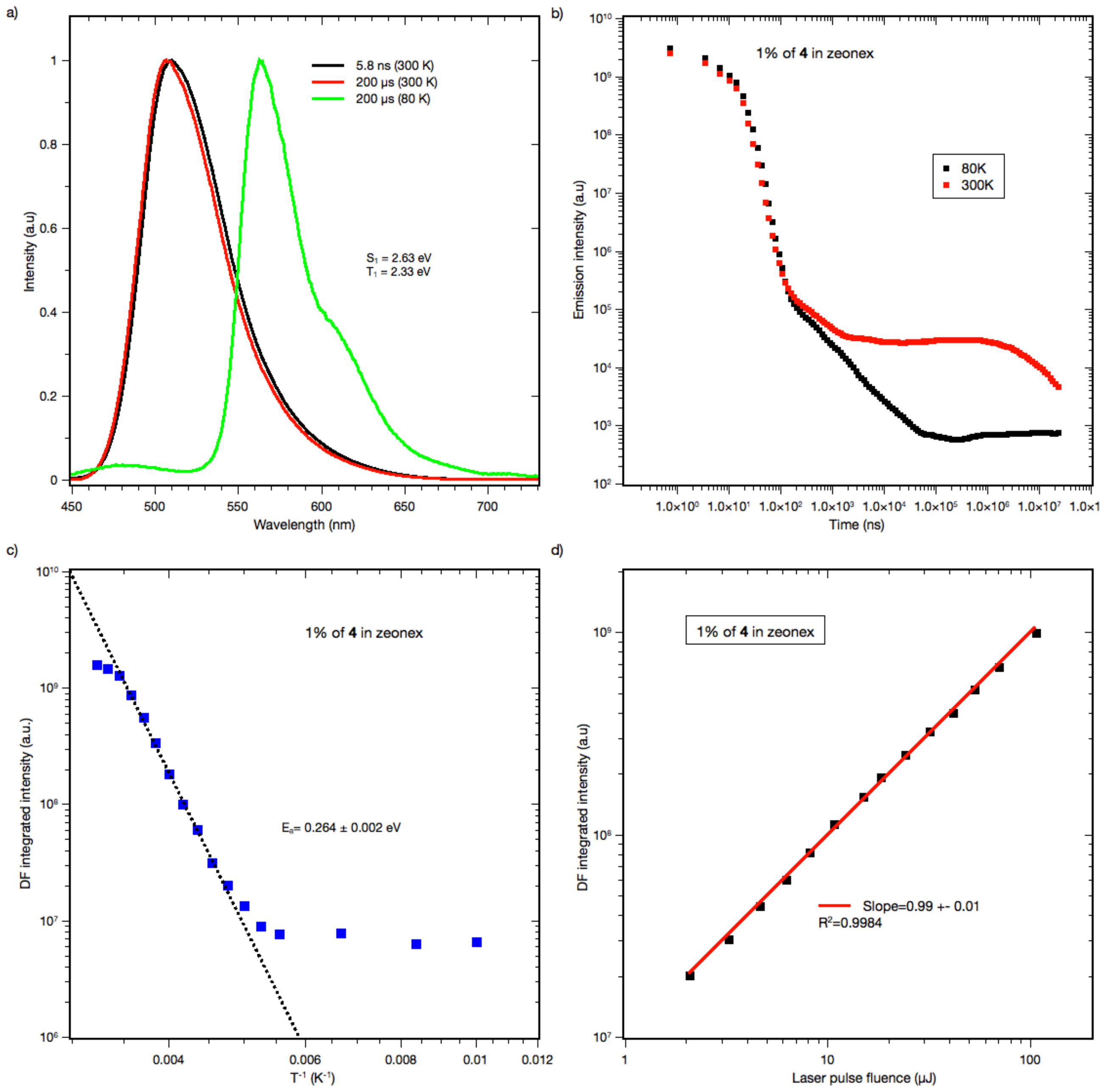

Figure S16. (a) PF, DF and PH spectra of 1 wt% **4**:zeonex® blended film. (b) Transient decays of 1 wt% **4**:zeonex® blended film at 300K and 80K. (c) Temperature dependence of delayed fluorescence of 1 wt% **4**:zeonex® blended film. (d) Power dependence of delayed fluorescence of 1 wt% **4**:zeonex® blended film at 300K.

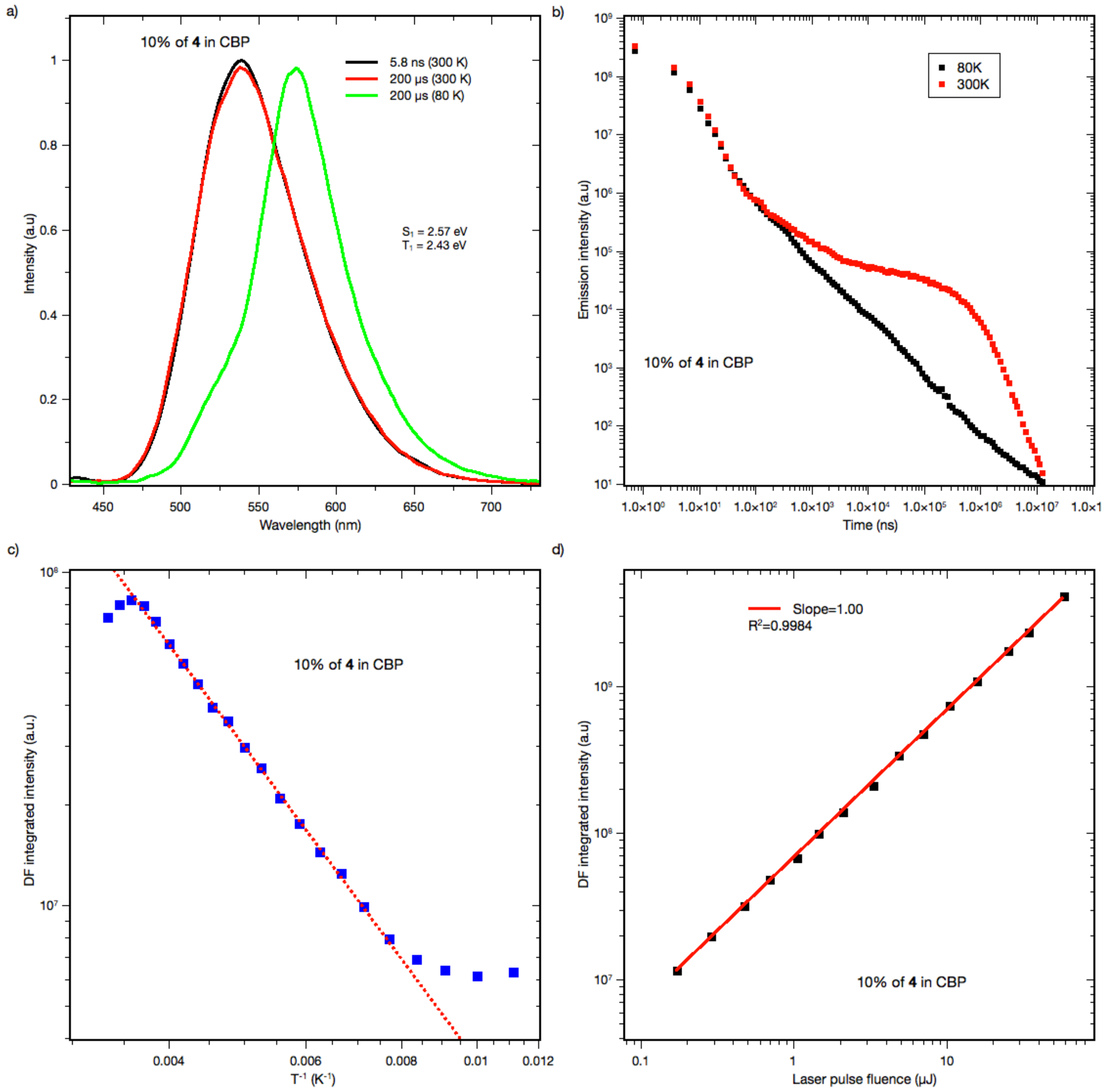

Figure S17. (a) PF, DF and PH spectra of 10 wt% **4**:CBP blended film. (b) Transient decays of 10 wt% **4**:CBP blended film at 300K and 80K. (c) Temperature dependence of delayed fluorescence of 10 wt% **4**:CBP blended film. (d) Power dependence of delayed fluorescence of 10 wt% **4**:CBP blended film at 300K.

Thermogravimetric Analysis (TGA)

The TGA profiles of solid samples **1**–**4** were recorded under the N_2 gas flow (200 mL/min), starting from 40 ˚C to 800 ˚C at the ramp rate of 10 °C/min.

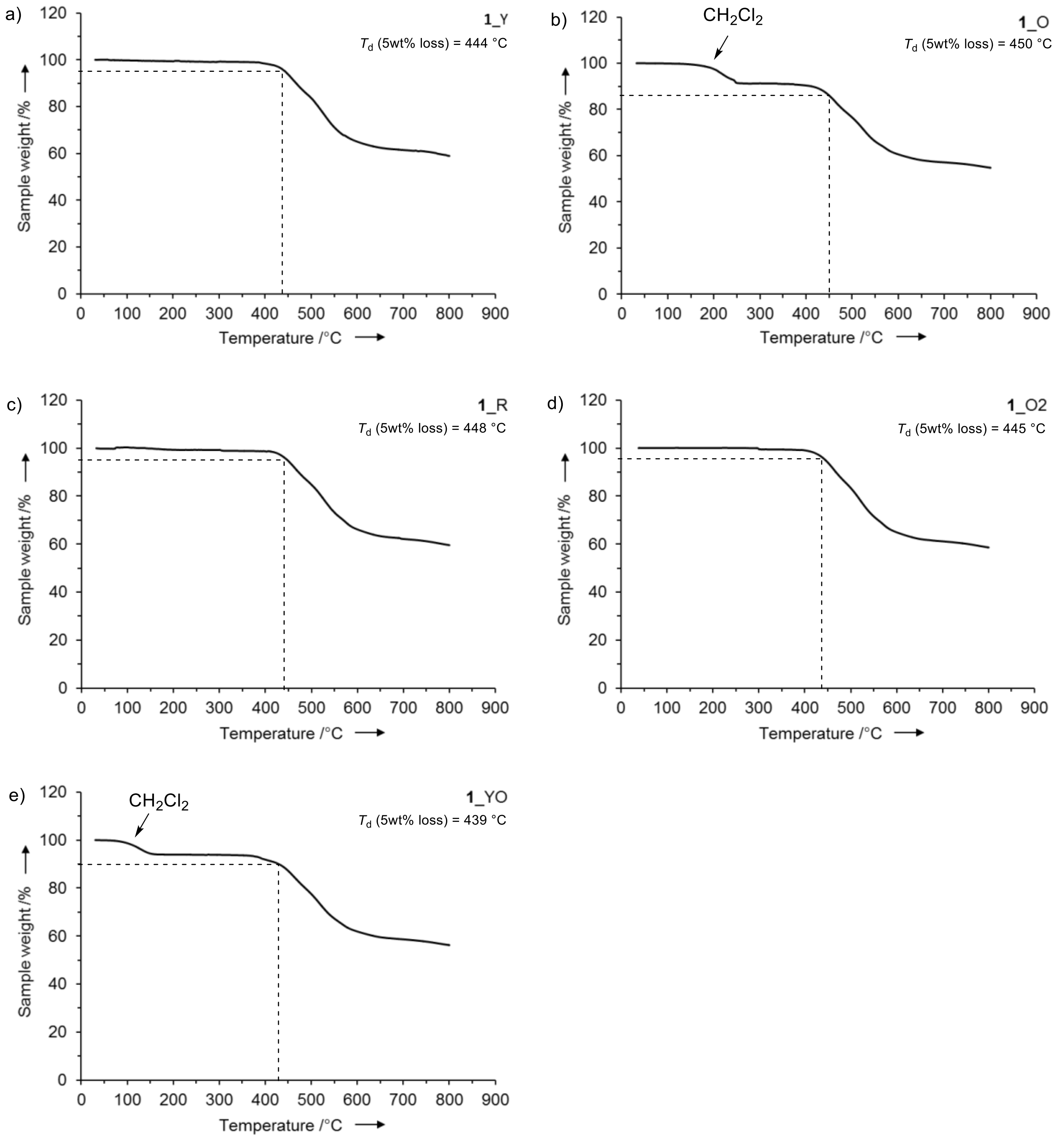

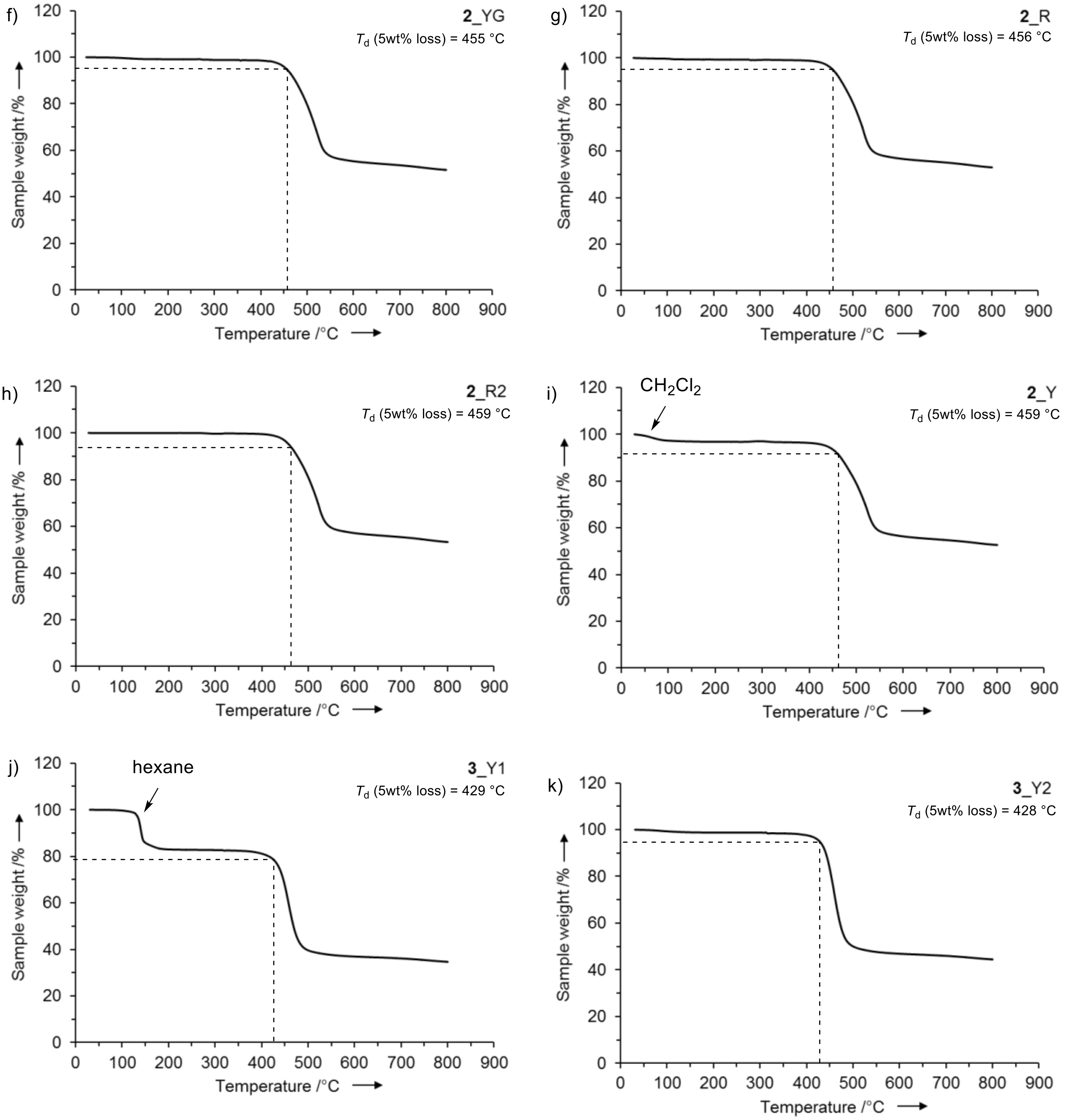

f)
2_YG
T_d (5wt% loss) = 455 °C
g)
2_R
T_d (5wt% loss) = 456 °C
h)
2_R2
T_d (5wt% loss) = 459 °C
i)
CH_2Cl_2
2_Y
T_d (5wt% loss) = 459 °C
j)
hexane
3_Y1
T_d (5wt% loss) = 429 °C
k)
3_Y2
T_d (5wt% loss) = 428 °C
Sample weight /%
Temperature /°C

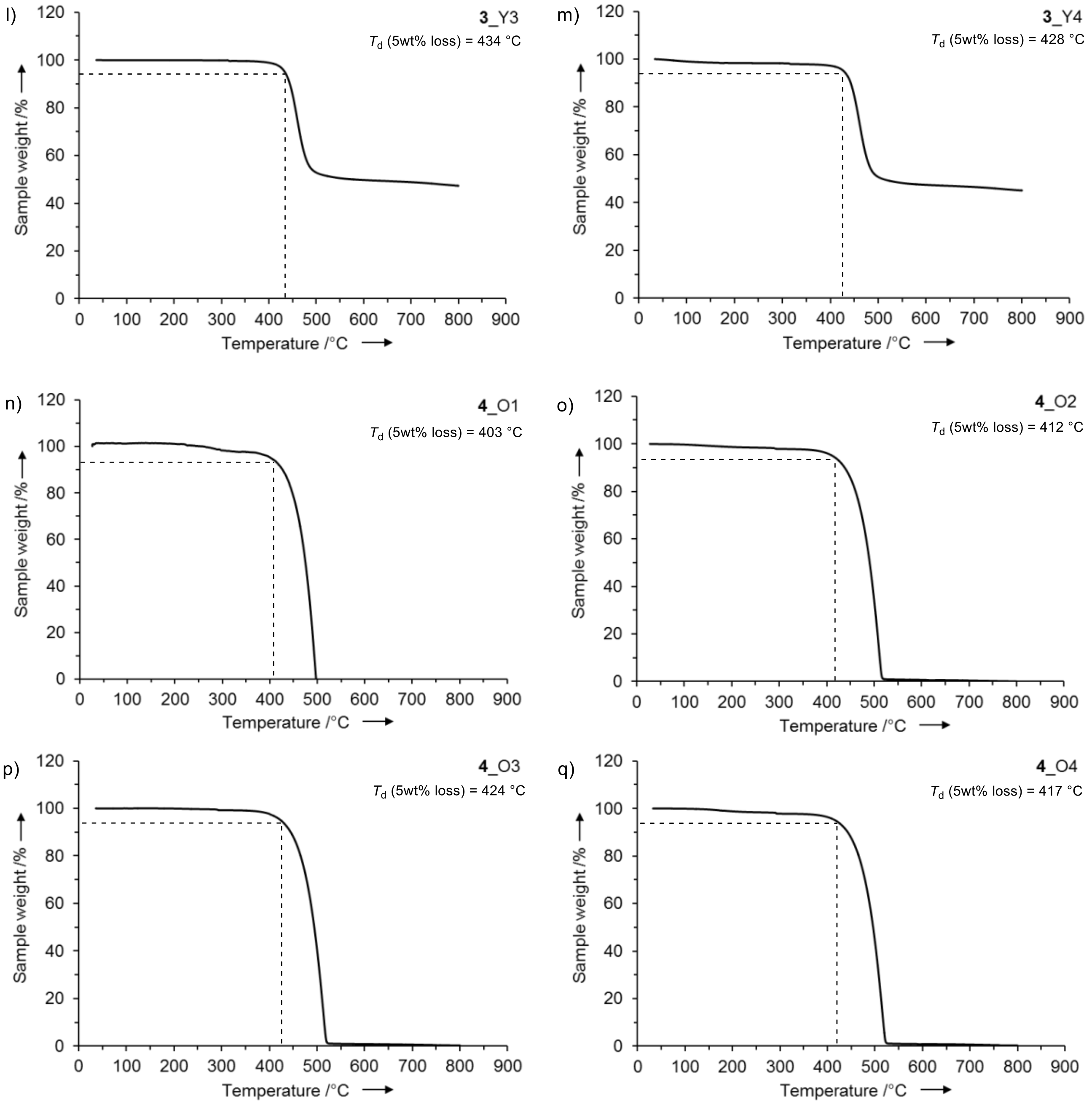

Figure S18. TGA profiles of **1–4**.

Cyclic Voltammograms

CV experiments were conducted at room temperature using degassed CH_2Cl_2 solutions of **1**–**4** (concentration: 1.0×10^{-3} M) containing 0.1 M n-Bu_4PF_6 as a supporting electrolyte in a cell equipped with a Pt as the working electrode (scanning rate: 100 mV/s). A Pt and Ag wires were applied as the counter and the reference electrode, respectively. The HOMO/LUMO energy levels were calculated by the following equation using the onset potentials corrected against the Fc/Fc^+ (Fc = ferrocene) couple: $E_{HOMO} = -(5.1 + {}^{ox}E_{onset})$ [eV]; $E_{LUMO} = -(5.1 + {}^{red}E_{onset}$ vs. $Fc/Fc^+)$ [eV].[S9–S12]

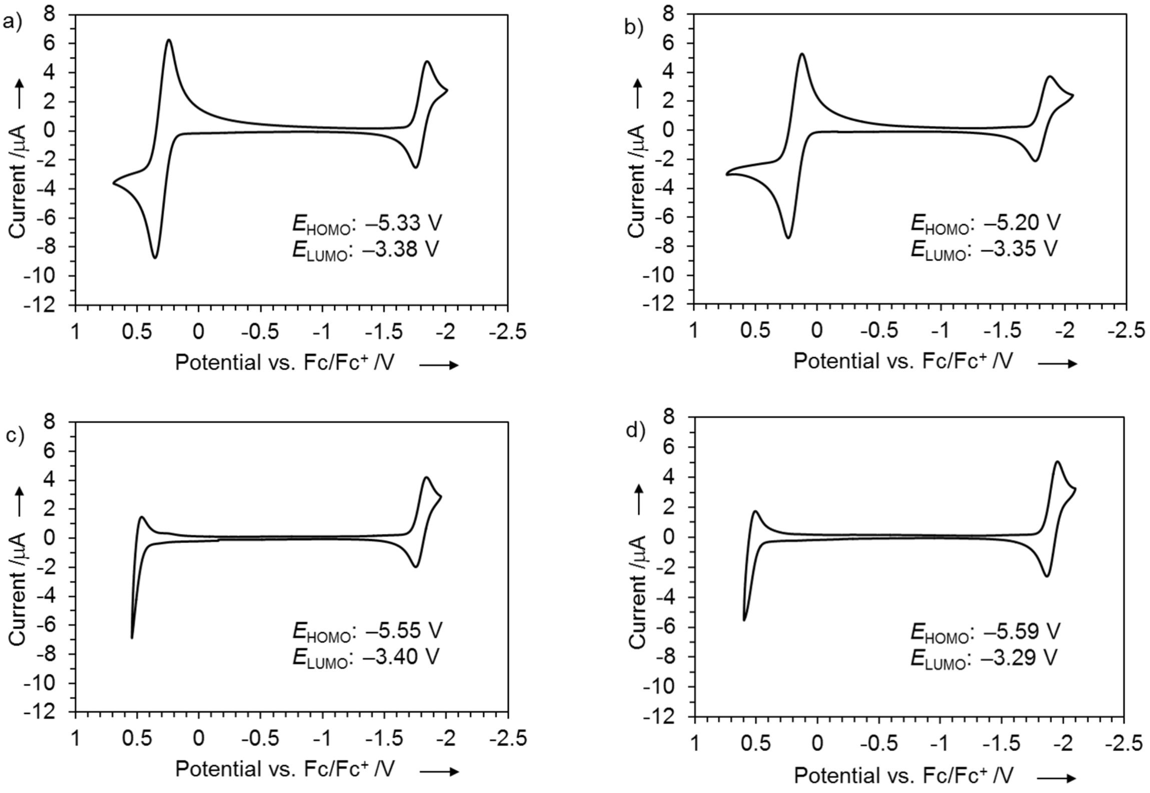

Figure S19. Cyclic voltammograms of a) **1**, b) **2**, c) **3**, and d) **4**.

NMR Charts

^{1}H NMR: (400 MHz, $CDCl_3$)

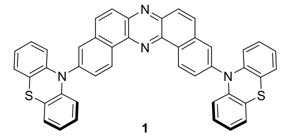

1

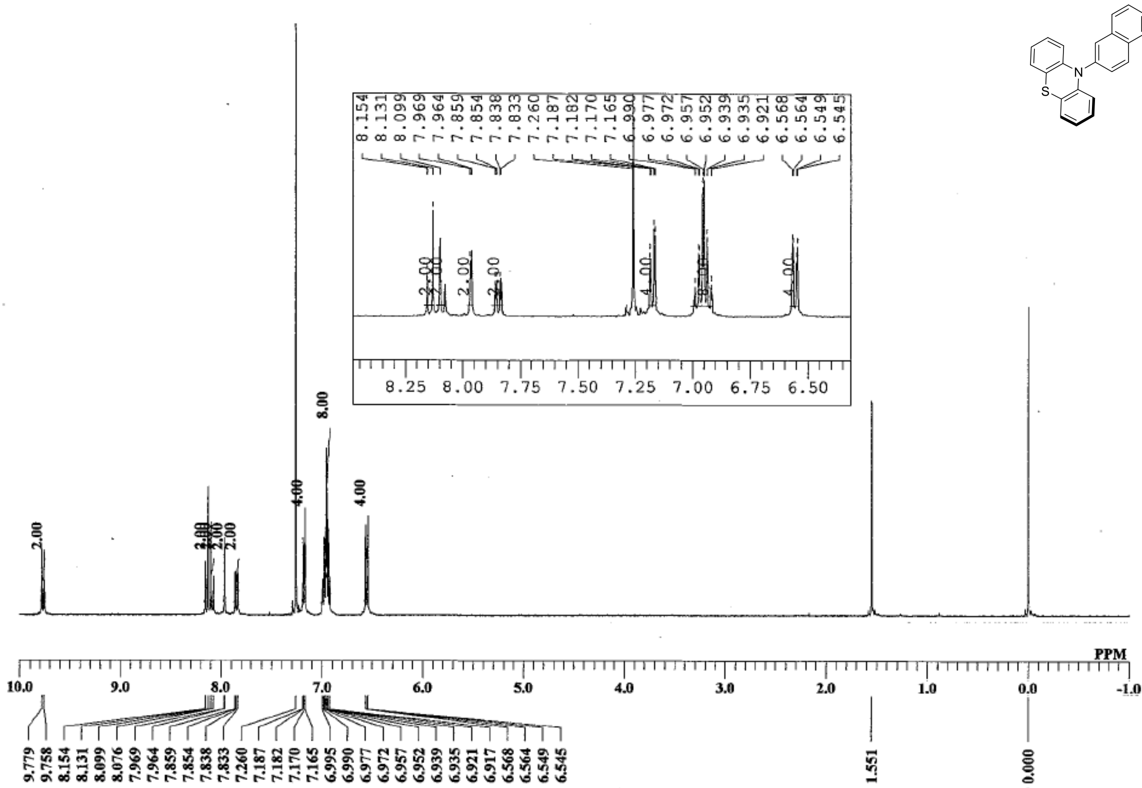

^{13}C NMR: (100 MHz, $CDCl_3$)

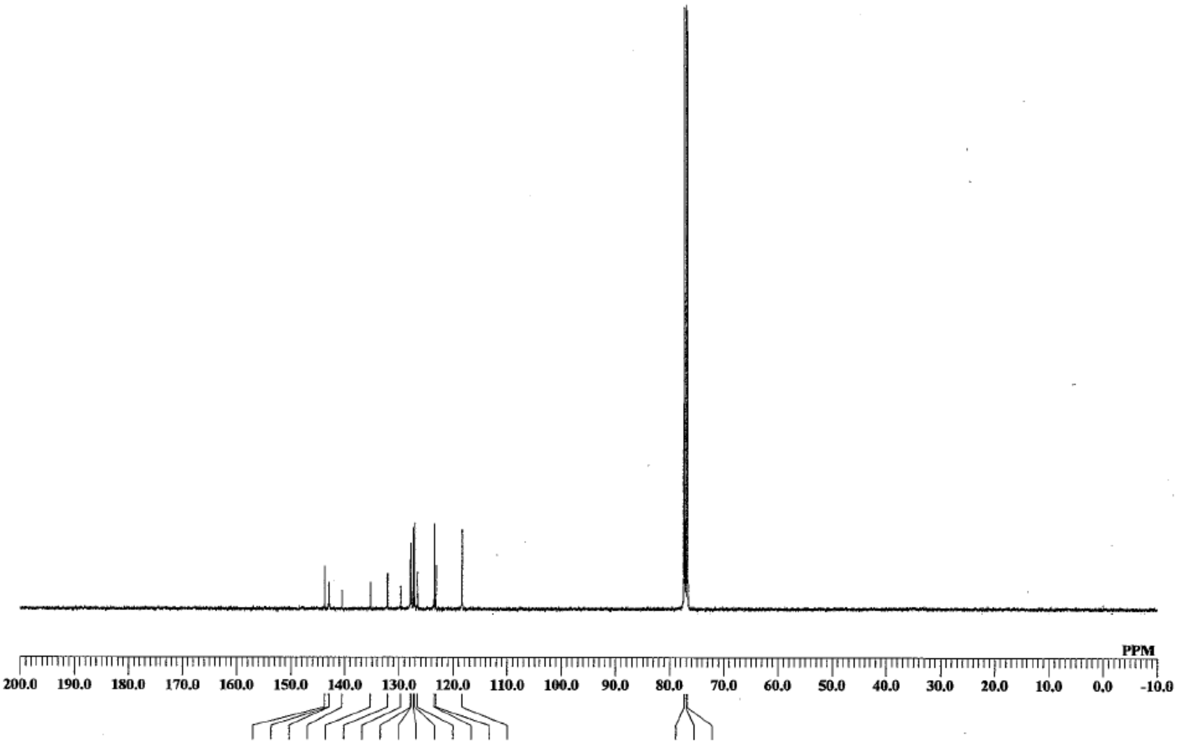

^{1}H NMR: (400 MHz, $CDCl_3$)

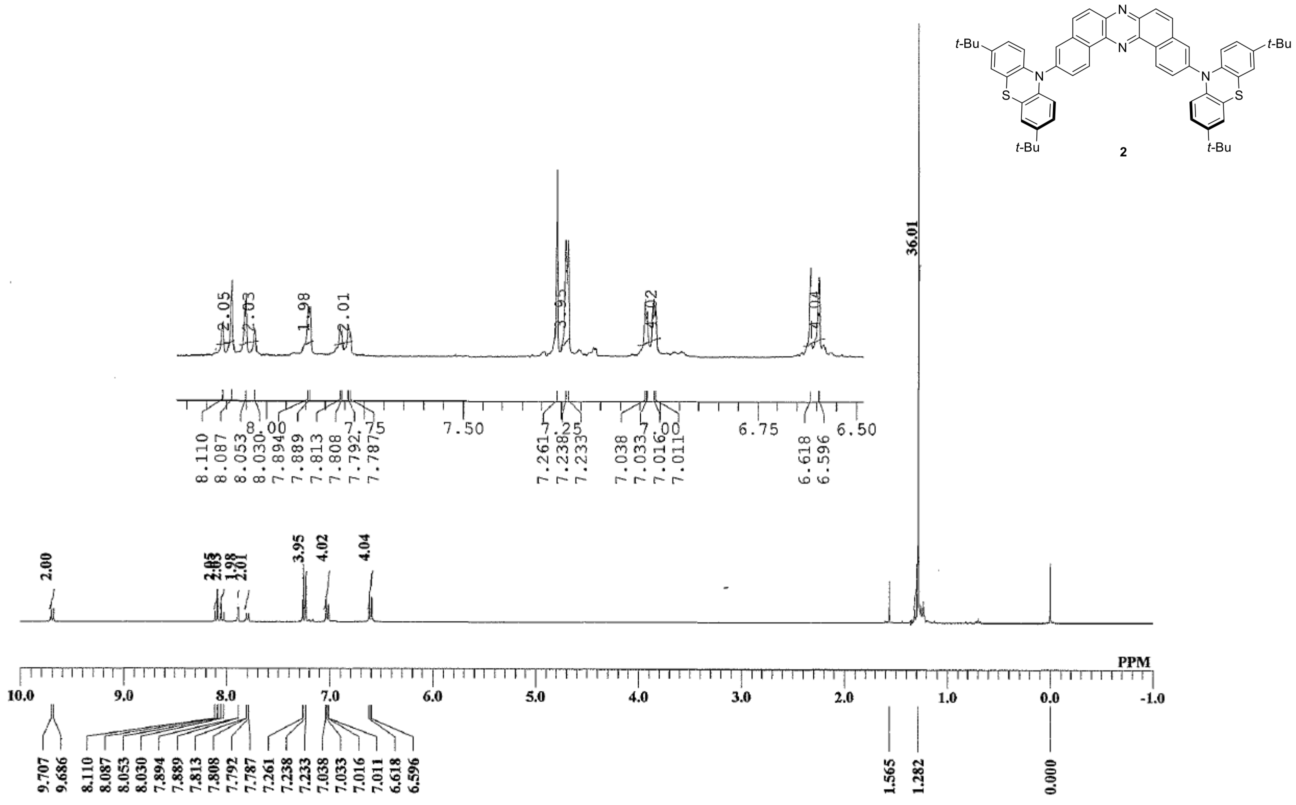

^{13}C NMR: (100 MHz, $CDCl_3$)

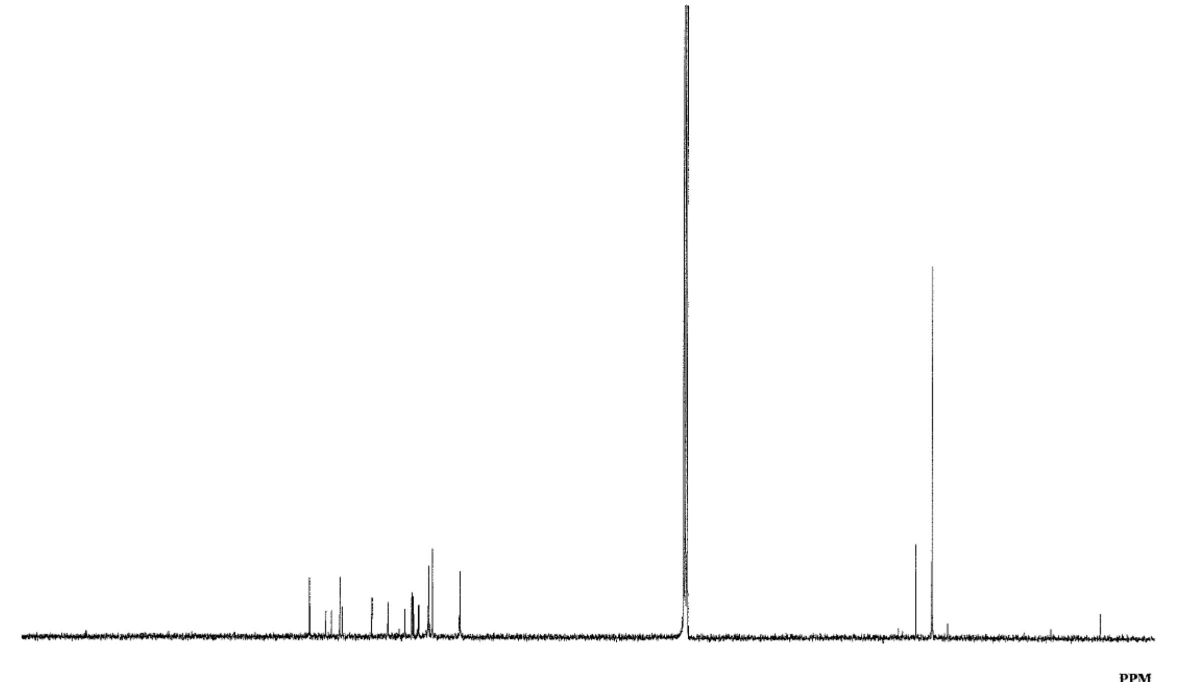

^{1}H NMR: (400 MHz, $CDCl_3$)

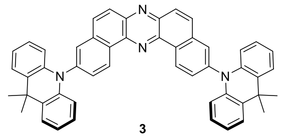

3

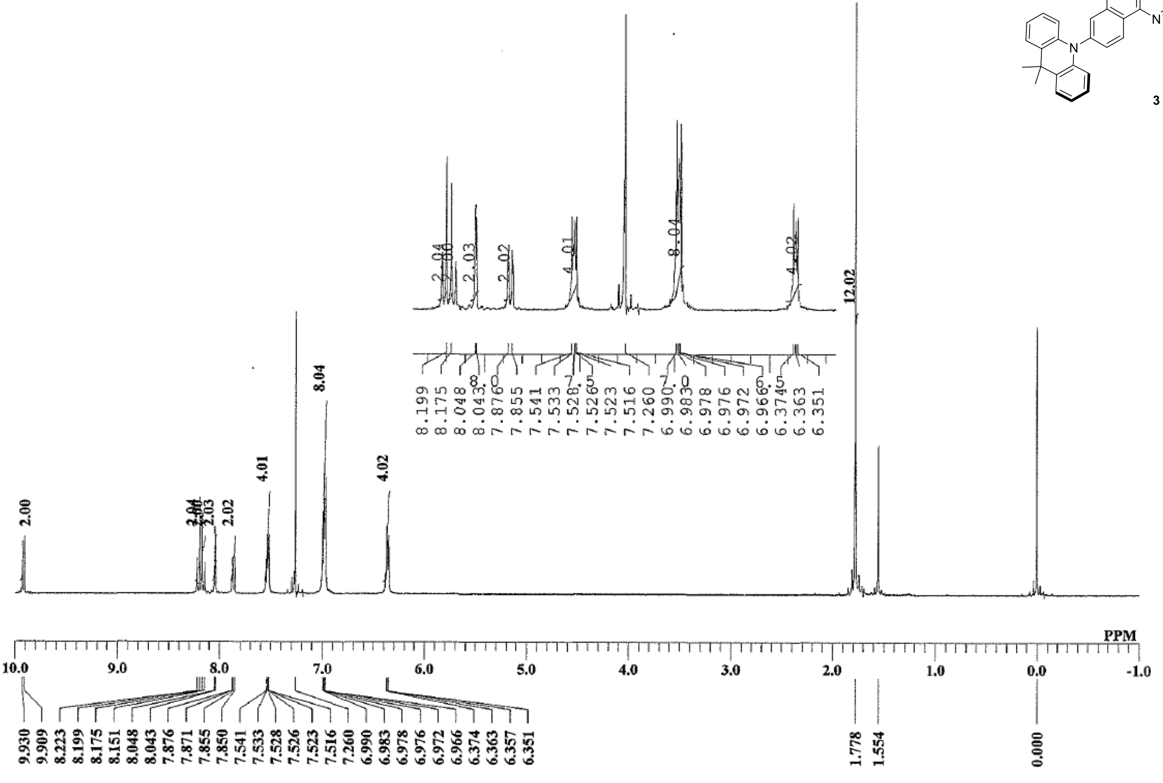

^{13}C NMR: (100 MHz, $CDCl_3$)

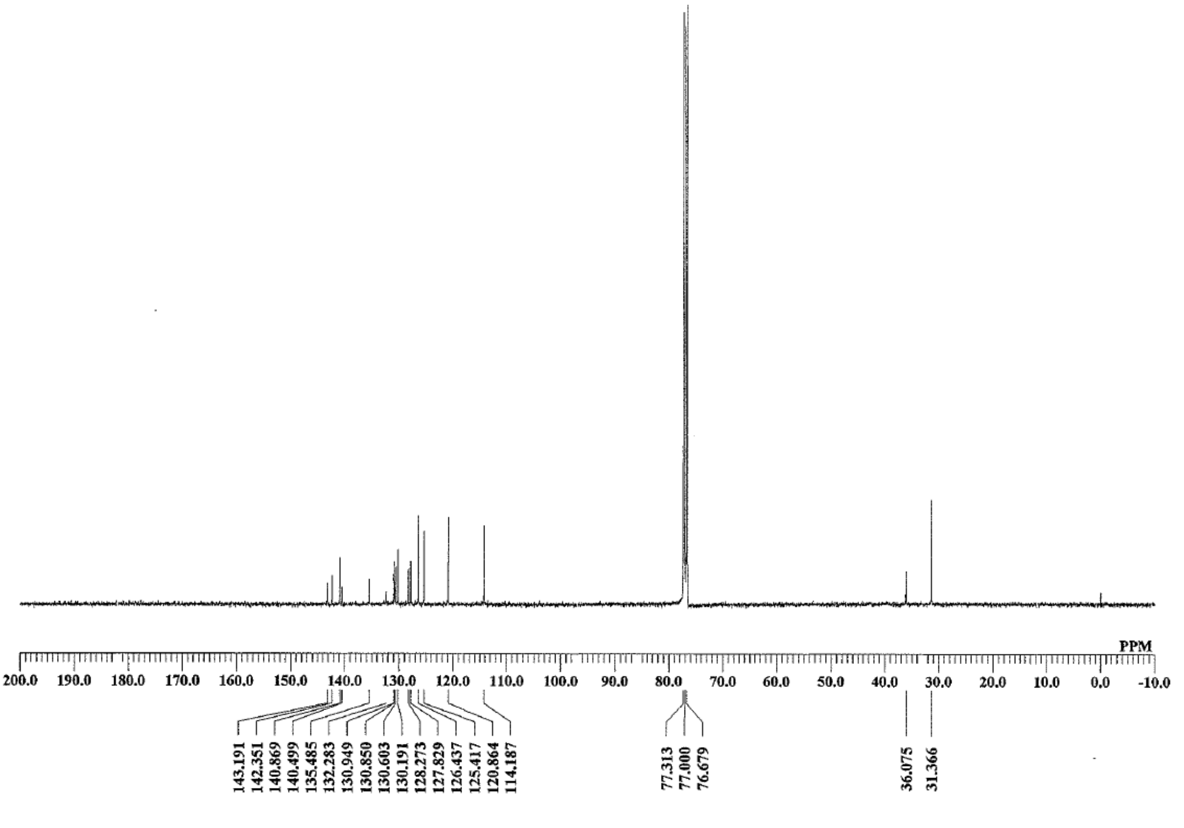

^{1}H NMR: (400 MHz, $CDCl_3$)

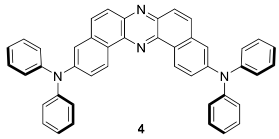

4

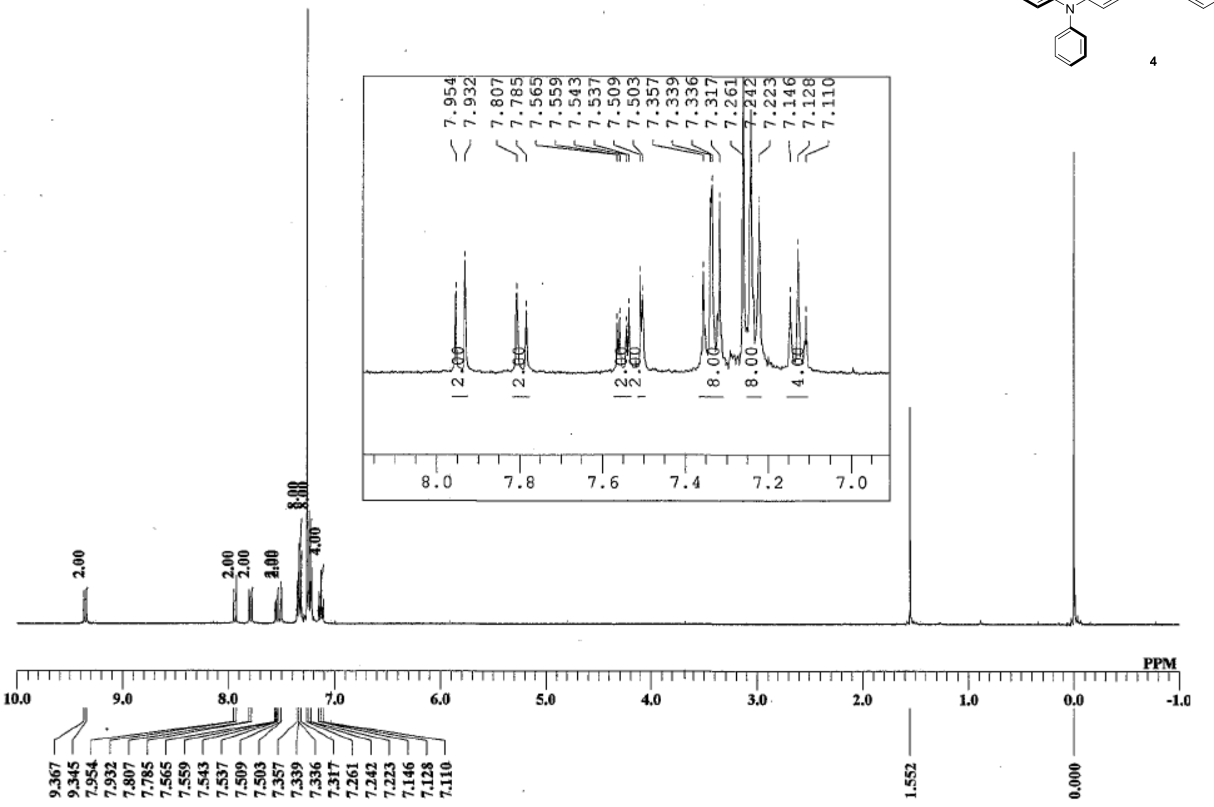

^{13}C NMR: (100 MHz, $CDCl_3$)

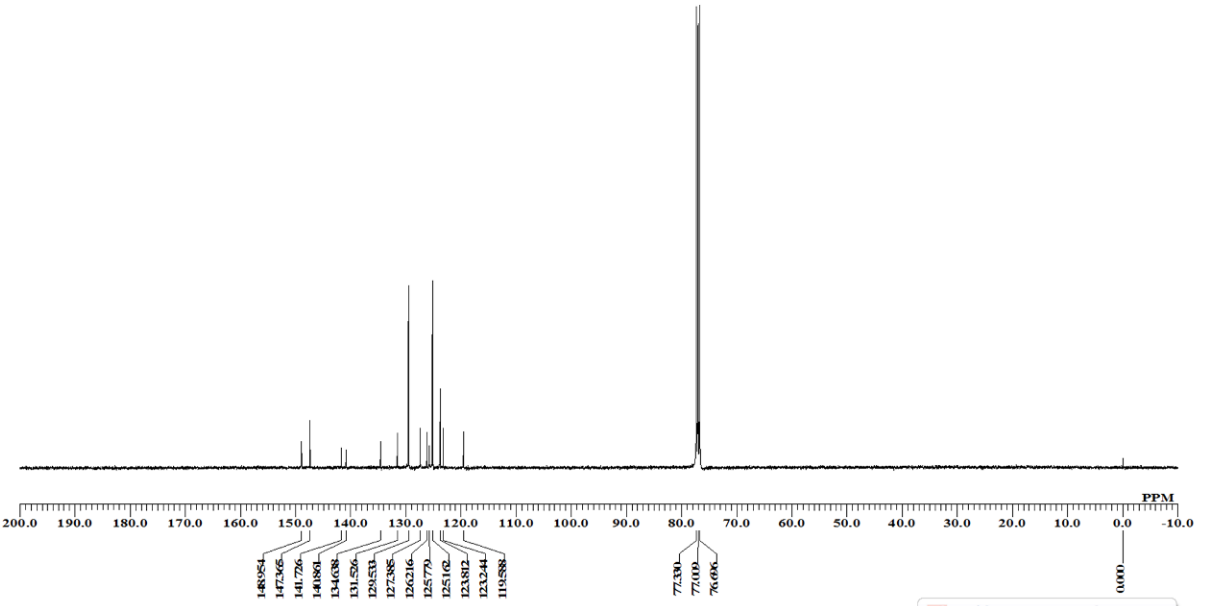

References

(S1) Yan, P.; Millard, A. C.; Wei, M.; Loew, L. M. *J. Am. Chem. Soc.* **2006**, *128*, 11030–11031.

(S2) Vilches-Herrera, M.; Miranda-Sepúlveda, J.; Rebolledo-Fuentes, M.; Fierro, A.; Lühr, S.; Iturriaga-Vasquez, P.; Cassels, B. K.; Reyes-Parada, M.; *Bioorg. Med. Chem.* **2009**, *17*, 2452–2460.

(S3) Kormos, A.; Móczár, I.; Sveiczer, A.; Baranyai, P.; Párkányi, L.; Tóth, K.; Huszthy, P. *Tetrahedron* **2012**, *68*, 7063–7069.

(S4) Andrew, T. L.; Swager, T. M. *J. Org. Chem.* **2011**, *76*, 2976–2993.

(S5) Takeda, Y.; Okazaki, M.; Minakata, S. *Chem. Commun.* **2014**, *50*, 10291–10294.

(S6) Ono, K.; Joho, M.; Saito, K.; Tomura, M.; Matsushita, Y.; Naka, S.; Okada, H.; Onnagawa, H. *Eur. J. Inorg. Chem.* **2006**, 3676–3683.

(S7) Altomare, A.; Cascarano, G.; Giacovazzo, C.; Guagliardi, A.; Burla, M.; Polidori, G.; Camalli, M. *J. Appl. Cryst.* **1994**, 27, 435–436.

(S8) Gaussian 09, Revision A.02, Frisch, M. J.; Trucks, G. W.; Schlegel, H. B.; Scuseria, G. E.; Robb, M. A.; Cheeseman, J. R.; Scalmani, G.; Barone, V.; Mennucci, B.; Petersson, G. A.; Nakatsuji, H.; Caricato, M.; Li, X.; Hratchian, H. P.; Izmaylov, A. F.; Bloino, J.; Zheng, G.; Sonnenberg, J. L.; Hada, M.; Ehara, M.; Toyota, K.; Fukuda, R.; Hasegawa, J.; Ishida, M.; Nakajima, T.; Honda, Y.; Kitao, O.; Nakai, H.; Vreven, T.; Montgomery Jr., J. A.; Peralta, J. E.; Ogliaro, F.; Bearpark, M.; Heyd, J. J.; Brothers, E.; Kudin, K. N.; Staroverov, V. N.; Kobayashi, R.; Normand, J.; Raghavachari, K.; Rendell, A.; Burant, J. C.; Iyengar, S. S.; Tomasi, J.; Cossi, M.; Rega, N.; Millam, J. M.; Klene, M.; Knox, J. E.; Cross, J. B.; Bakken, V.; Adamo, C.; Jaramillo, J.; Gomperts, R.; Stratmann, R. E.; Yazyev, O.; Austin, A. J.; Cammi, R.; Pomelli, C.; Ochterski, J. W.; Martin, R. L.; Morokuma, K.; Zakrzewski, V. G.; Voth, G. A.; Salvador, P.; Dannenberg, J. J.; Dapprich, S.; Daniels, A. D.; Farkas, O.; Foresman, J. B.; Ortiz, J. V.; Cioslowski, J.; Fox, D. J. Gaussian, Inc., Wallingford CT, 2009.

(S9) Rybakiewicz, R.; Gawrys, P.; Tsikritzis, D.; Emmanouil, K.; Kennou, S.; Zagorska, M.; Pron, A. *Electrochim. Acta* **2013**, *96*, 13–17.

(S10) Trasatti, S. *Pure Appl. Chem.* **1986**, *58*, 955–966.

(S11) Data, P.; Motyka, R.; Lapkowski, M.; Suwinski, J.; Monkman, A. P. *J. Phys. Chem. C* **2015**, *119*, 20188– 20200.

(S12) Bredas, J.-L. *Mater. Horiz.* **2014**, *1*, 17–19.
